# Comparative efficacy and safety of PD-1/PD-L1 inhibitors in triple negative breast cancer: a systematic review and network meta-analysis of randomized controlled trials

**DOI:** 10.1186/s12935-023-02941-7

**Published:** 2023-05-11

**Authors:** Ibrahim Elmakaty, Ruba Abdo, Ahmed Elsabagh, Abdelrahman Elsayed, Mohammed Imad Malki

**Affiliations:** 1 College of Medicine, QU Health, Qatar University, P. O. Box: 2713, Doha, Qatar; 2 Pathology Unit, Department of Basic Medical Sciences, College of Medicine, QU Health, Qatar University, Doha, Qatar

**Keywords:** PD-1/PD-L1 inhibitors, Immune checkpoint inhibitors, Overall survival, Progression-free survival, Pathological complete response, Adverse events, Efficacy, Safety, Systematic review, Network meta-analysis

## Abstract

**Background:**

Triple-Negative Breast Cancer (TNBC) is a lethal subtype of breast cancer with limited treatment options. The purpose of this Network Meta-Analysis (NMA) is to compare the efficacy and safety of inhibitors of programmed cell death 1 (PD-1) and programmed cell death ligand 1 (PD-L1) in treating TNBC.

**Methods:**

Our search strategy was used in six databases: PubMed, Cochrane Library, Cumulative Index to Nursing and Allied Health Literature database, Embase, Scopus, and Web of Science up to November 2nd, 2022, as well as a thorough search in the most used trial registries. We included phase II and III randomized controlled trials that looked at the efficacy of PD-1/PD-L1 inhibitors in the treatment of TNBC and reported either Overall Survival (OS), Progression-Free Survival (PFS), or pathological Complete Response (pCR). The risk of bias was assessed utilizing Cochrane's risk of bias 2 tool, and the statistical analysis was performed using a frequentist contrast-based method for NMA by employing standard pairwise meta-analysis applying random effects model.

**Results:**

12 trials (5324 patients) were included in our NMA including seven phase III trials. Pembrolizumab in a neoadjuvant setting achieved a pooled OS of 0.82 (95% Confidence Interval (CI) 0.65 to 1.03), a PFS of 0.82 (95% CI 0.71 to 0.94) and a pCR 2.79 (95% CI 1.07 to 7.24) compared to Atezolizumab’s OS of 0.92 (95% CI 0.74 to 1.15), PFS of 0.82 (95% CI 0.69 to 0.97), and pCR of 1.94 (95% CI 0.86 to 4.37). Atezolizumab had less grade ≥ 3 adverse events (OR 1.48, 95% CI 0.90 to 2.42) than Pembrolizumab (OR 1.90, 95% CI 1.08 to 3.33) in the neoadjuvant setting.

**Conclusions:**

PD-1/PD-L1 inhibitors exhibited varying efficacy in terms of OS, PFS, and pCR. They were associated with an increase in immune-related adverse effects. When used early in the course of TNBC, PD-1/PD-L1 inhibitors exert their maximum benefit. Durvalumab as a maintenance treatment instead of chemotherapy has shown promising outcomes. Future studies should focus on PD-L1 expression status and TNBC subtypes, since these factors may contribute to the design of individualized TNBC therapy regimens.

*Systematic review registration* PROSPERO Identifier: CRD42022380712.

**Supplementary Information:**

The online version contains supplementary material available at 10.1186/s12935-023-02941-7.

## Background

Breast cancer remains a major health burden, causing considerable morbidity and mortality worldwide [1]. It has surpassed lung cancer as the most frequently diagnosed malignancy overall and ranks the fifth leading cause of cancer-related mortality, with an estimated 2.3 million new cases (11.7% of all cancers), and 685,000 deaths in 2020 [2]. The incidence rate has been increasing at an alarming rate over the past years, especially in transitioning countries, and it is predicted that by 2040, this burden will grow further by over 40% to about 3 million new cases and 1 million deaths every year [2, 3]. Triple-Negative Breast Cancer (TNBC) is a particularly aggressive subtype that accounts for approximately 15–20% of all cases and is characterized by a lack of expression of both estrogen and progesterone receptors as well as human epidermal growth factor receptor 2 [4]. The high molecular heterogeneity, great metastatic potential, and limited therapeutic options have all contributed to TNBC having a relatively poor prognosis with a 5-year overall survival rate of 77% [5, 6]. Due to the absence of well-defined molecular targets, TNBC therapy predominantly relies on the administration of Taxane and Anthracycline-based regimens in both the neoadjuvant and the adjuvant settings [4, 6, 7]. More favorable response rates are shown to be achieved when using a combination rather than single-agent chemotherapy [8, 9]. Although this can be effective initially, chemotherapy is often accompanied by resistance, relapse, and high toxicity [10, 11]. Additionally, survival rates in those who develop metastatic disease have not changed over the past 20 years [9]. The median Overall Survival (OS) for those patients with the current treatment option is 16 months and the median Progression-Free Survival (PFS) is 5.6 months [12]. These results underscore the urgent need for more effective and less toxic therapies.

The introduction of immunotherapy has revolutionized the field of oncology over the past decade and has been successfully incorporated into the standard treatment paradigm of many malignancies including non-small cell lung cancer and renal cell cancer [13, 14]. Whilst breast cancer has traditionally been considered immunogenically quiescent, several lines of evidence have demonstrated TNBC to be highly immunogenic and feature a microenvironment that is enriched with stromal Tumor Infiltrating Lymphocytes (TILs) with a relatively high tumor mutational burden as opposed to other subtypes [15, 16]. The high levels of inhibitory checkpoint molecules expressed on the TILs led to the successful implementation of Immune Checkpoint Inhibitors (ICI) in TNBC treatment, particularly inhibitors of the Programmed Cell Death 1 (PD-1) and the Programmed Cell Death Ligand 1 (PD-L1) which have shown great promise in the field’s clinical trials [15]. The PD‑L1/PD-1 signaling pathway exerts a critical role in forming an adaptive immune resistance mechanism that mediates tumor invasion and metastasis [17]. Blocking this pathway would therefore restore the antitumor immune responses by reducing the inhibition of innate immunity and reactivating tumor-specific cytotoxic T cells [18].

Atezolizumab, an anti-PD-L1 antibody was the first Food and Drug Administration (FDA) approved ICI given along with nab-paclitaxel for patients with unresectable locally advanced or metastatic TNBC whose tumors express PD-L1 [19]. This accelerated approval was based on the results of the Impassion130 trial. Unfortunately, the designated confirmatory trial, IMpassion131 neither met the primary endpoint of PFS superiority nor achieved statistically significant overall OS leading to the withdrawal of this combination as an indication for treatment [12]. Alternatively, FDA granted approval to pembrolizumab, a PD-1 inhibitor to be used in combination with chemotherapy for patients with high-risk, early-stage TNBC, as well as those with locally recurrent unresectable or metastatic TNBC whose tumors have a PD-L1 Combined Positive Score (CPS) of ≥ 10 [12]. Nonetheless, there remain several additional clinical trials that have assessed the role of anti‑PD‑L1/PD‑1 agents in TNBC treatment with inconsistent results. The objective of this Network Meta-Analysis (NMA) is to evaluate the efficacy and safety of these agents, as well as compare them in order to determine the optimal therapeutic regimen for patients with TNBC.

## Methods

### Protocol and registration

This systematic review and meta-analysis is reported following the Preferred Reporting Items for Systematic Reviews and Meta-Analyses (PRISMA) extension for NMA Additional file 1: (Table S1) [20]. The NMA protocol was carried in accordance with a protocol that had been registered in the International Prospective Register of Systematic Reviews (PROSPERO) online database (PROSPERO Identifier: CRD42022380712).

### Search strategy

We developed our search strategy in the PubMed database using Medical Subject Headings (MeSH) that included the terms (“Immune Checkpoint Inhibitors”[MeSH] OR “programmed cell death 1 receptor/antagonists and inhibitors”[MeSH]) AND “Triple Negative Breast Neoplasms”[MeSH] AND “Randomized Controlled Trial”[Publication Type] with multiple keywords build around them. There was no date or language restriction applied to our strategy. The developed search strategy was then transferred from PubMed to five other databases by the Polyglot translator [21], namely Cochrane Library, Cumulative Index to Nursing and Allied Health Literature database, Embase, Scopus, and Web of Science. All databases were searched from the inception date until the 2nd of November 2022. The yielded studies were then exported to EndNote X7, where duplicates were identified and excluded. The remaining articles were uploaded to the Rayyan platform for screening [22]. In addition, we searched popular clinical trial registries such as ClinicalTrials.gov, EU Clinical Trials Register, International Standard Randomised Controlled Trial Number registry, International Clinical Trials Registry Platform, and breastcancertrials.org for Gery literature (unpublished trials) to ensure the comprehensiveness of our search strategy. Additional file 1 contains the complete strategy for each database and trial registries.

### Eligibility criteria

We included trials that met the following criteria: (1) usage of FDA-approved PD-1/PD-L1 inhibitors, (2) phase II or III RCTs, (3) for the management of confirmed TNBC, (4) compared against a different Immune Checkpoint Inhibitors (ICIs), multiple agents’ chemotherapy regimen, single agent chemotherapy regimen or placebo (5) reported Hazard Ratios (HR) for OS, PFS or numbers of pathological Complete Response (pCR) in each both arms of the trial. We excluded review articles, non-randomized trials, quasi-randomized trials, meta-analyses and observational studies, as well as studies on animal models. We also excluded trials using non-FDA-approved immune checkpoint inhibitors.

### Study selection and screening

The records obtained from applying the search strategy were evaluated on the Rayyan platform [22]. Titles and abstracts were screened independently by two reviewers either IE/RA or AhE/AbE with any disagreements were resolved by consensus among the entire team (IE, RA, AhE, AbE and MIM). The full texts of studies that were deemed potentially eligible were then retrieved and double-screened independently (IE/RA or AhE/AbE), with discrepancies dealt with through discussion with the whole team (IE, RA, AhE, AbE and MIM).

### Data extraction

We extracted information from each eligible study on the first author, publication date, phase, total number of patients included, and number of patients in each arm, as well as patient demographics (median age, cancer stage), treatment given in each arm, duration of treatment, follow-up time and percentage of patients with positive PD-L1 expression at baseline defined by CPS ≥ 1. We also extracted HR values and their 95% Confidence Intervals (CI) for OS and PFS from each study, as well as the number of patients who achieved pCR in both arms. We collected data on the occurrence of common Adverse Events (AEs) in patients from each study arm. When duplicate publications were discovered, only the most recent and complete reports of RCTs were included. Two reviewers extracted all data (IE/RA or AhE/AbE), which was then summarized, discussed by the team, and compiled into an online Microsoft Excel spreadsheet accessible to all authors.

### Risk of bias assessment

To assess the risk of bias, version 2 of the Cochrane Risk-Of-Bias (RoB2) assessment tool for randomized trials was used [23]. This was done independently by the reviewers (IE/RA or AhE/AbE) with disagreement being resolved by discussion and input from a third author (MIM). The RoB2 assessment tool includes five distinct domains with multiple signaling questions to aid in assessing the risk of bias. The five domains in this tool appraise bias arising from the following: randomization process, deviations from intended interventions, missing outcome data, measurement of the outcome and selection of the reported result. Accordingly, the signaling questions provided by the ROB2 tool were answered, and the two other reviewers evaluating the trial used those answers to categorize the current domain as “low risk of bias,” “some concerns,” or “high risk of bias.” The reviewer's judgment in each domain resulted in an overall risk-of-bias conclusion for the trial under consideration. The study was deemed to have a “low risk of bias” if it was judged to have a low risk of bias in all domains included in the tool, “some concerns” if it raised some concerns in at least one domain, or “high risk of bias” if it was judged to have a high risk of bias in at least one or some concerns for multiple domains, significantly lowering confidence in the result. This data for all studies was compiled in the tool's template excel sheet, which was made available to all reviewers.

### Outcomes

As our aim is to evaluate the efficacy and safety of ICIs, we selected four different outcomes in this NMA. The first two are OS, which is defined as the time from randomization to death from any cause, and PFS, which is defined as the time from randomization to the first documented disease progression per Response Evaluation Criteria in Solid Tumors version 1.1. The HR and its 95% CI comparing the two arms of the trials in Intention-To-Treat (ITT) populations were used to generate our final effect sizes in this NMA. The third outcome is pCR, which is defined as the absence of invasive tumors in the breast and regional nodes at the time of definitive surgery (ypT0/is pN0). Finally, to assess the safety of PD-1/PD-L1 inhibitors, we estimated the likelihood of developing AEs in each arm of the ITT populations by using the number of patients who had AEs in all grades and grade 3 or higher. Both pCR and AEs were calculated using Odds Ratios (OR) and their 95% CI based on the number of reported events in each of the trial arms.

### Data analysis

Our NMA used standard pairwise meta-analysis implemented in multivariate meta-analysis models using a frequentist contrast-based approach [24]. If there is no evidence of importance in transitivity, a random-effects frequentist NMA has to be performed. These models assume that direct and indirect evidence are consistent. The network meta-analysis' net evidence is a weighted average of direct and indirect evidence. For OS and PFS, we calculated the mean log HR and its standard error and entered it into the model [25], while for pCR and AEs, we entered the number of events in each arm. When the same intervention was used in both arms of an RCT, it was assumed that the effect of that intervention was cancelled out, thus we assumed that all trials used the same comparator chemotherapy, which is necessary because even within the same trial, different chemotherapy regimens were used as controls. The assumption of transitivity was tested by comparing the distribution of study and population characteristics that may act as effect modifiers across the various pairwise comparisons. If transitivity issues were present, we returned to data extraction to verify the stage of TNBC, and the type of chemotherapy regimen used. In the case of indirect evidence, inconsistency between direct and indirect evidence was investigated locally through the use of symmetrical node-splitting [26]. However, we found no head-to-head comparisons of PD-1/PD-L1 inhibitors. Visual inspection of comparison-adjusted funnel plots for NMA was used to assess publication bias [27]. Studies were expected to form an inverted funnel centred at zero in the absence of small-study effects. The Surface Under the Cumulative Ranking Curve (SUCRA) value, which represents the re-scaled mean ranking, was also calculated and summarized [28]. Where quantitative synthesis is deemed invalid due to a small number of studies using the same intervention, narrative synthesis was used to report the findings in the results section, with estimates from the original studies. For all comparisons, we adopted the network suite in Stata to perform analyses and graphs, Stata version 16 (College Station, TX, USA) [29].

### Subgroup analysis

In the event of significant heterogeneity, we conducted a sensitivity analysis, removing each study and comparing its effect. In terms of the outcome of AEs, we investigated the impact of reported symptoms on AEs to check which side effects are likely to produce this effect. We performed a sensitivity analysis for NMA using the Generalized Pairwise Modelling (GPM) framework to investigate the effect of the models used [30]. The GPM framework was used to generate mixed treatment effects against a common comparator. The common comparator for all outcomes was chemotherapy. Other than transitivity, this framework requires no additional assumptions [30]. In this sensitivity analysis, the Inverse Variance Heterogeneity model was used to pool the meta-analytical estimates [31]. The H index was used to assess statistical heterogeneity across pooled direct effects, while the weighted pooled H index ($\overline{H }$) was used to examine inconsistency across the network and assess transitivity [30]. The smallest value that H and $\overline{H }$ can take is 1, and $\overline{H }$ <3 was thought to represent minimal inconsistency [32]. MetaXL version 5.3 was used for the GPM framework analyses (EpiGear Int Pty Ltd.; Brisbane, Australia). The results of those sensitivity analyses will be presented in the Additional file 1.

## Results

### Study selection

**Fig. 1:**
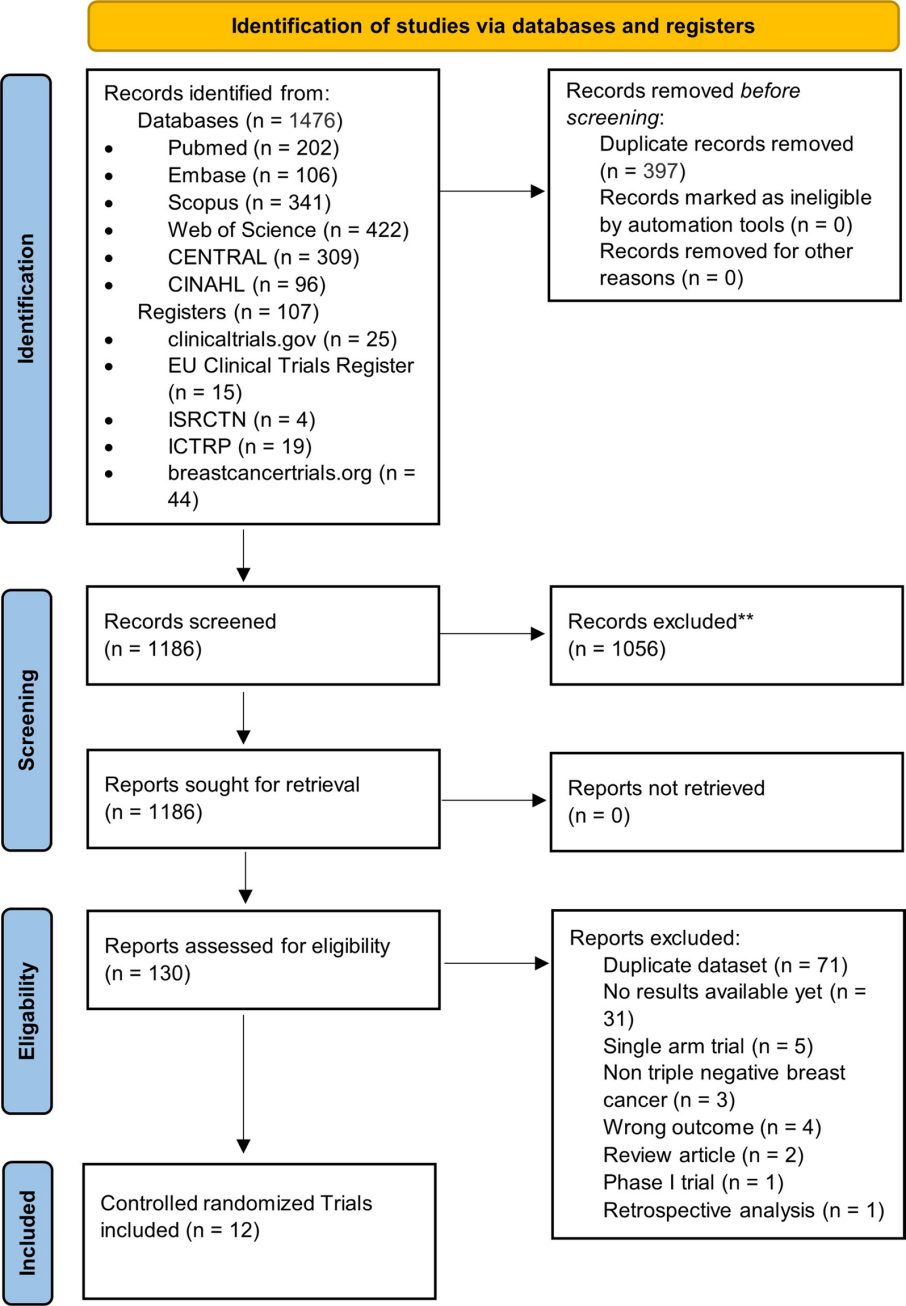
PRISMA flowchart showing the number of studies at each stage of conducting this NMA

Figure 1 illustrates the PRISMA flow diagram of the study selection process. Our extensive database and trial registry search yielded 1583 results. 397 duplicates were automatically removed through EndNote. A total of 1186 potentially relevant articles were identified, of which 1056 were excluded after the initial review of their titles and abstracts. The full text of the remaining 130 articles was assessed for eligibility. Of those, 71 were found to be duplicate patient records, and only the most recent and inclusive records were kept. Another 31 RCTs were excluded due to a paucity of outcome measures at the time of the search. Other 16 records were similarly removed for a variety of reasons depicted in Fig. 1. Eventually, 12 studies were eligible for inclusion in our NMA [33–44]. Additional file 1: Table S2 includes all the additional information on the omitted record citations as well as full reasoning.

### Study characteristics and data collection

Table 1 summarizes the characteristics of the included RCTs. All 12 trials included were two-arm trials that reported results from 5324 patients with median ages ranging from 48 to 59.1 years. There were seven phase III trials and five phase II trials. Six studies looked at the effect of PD-1/PD-L1 inhibitors on unresectable, invasive, or metastatic (advanced) TNBC [33, 35–37, 40, 43], four looked at non-metastatic/early-stage TNBC [39, 41, 42, 44], and two looked at treated metastatic TNBC for maintenance therapy [34, 38]. Atezolizumab (n = 5 trials) was the most commonly studied ICI [33, 36, 39, 40], followed by Pembrolizumab (n = 4 trials) [34, 35, 41, 42], Durvalumab (n = 2 trials) [37, 38], and Nivolumab (n = 1 trial) [43]. Six trials used multiple-agent chemotherapy regimens in combination with PD-1/PD-L1 inhibitors [36, 37, 39, 41, 42, 44], and four used mono-chemotherapy regimens with PD-1/PD-L1 inhibitors, including two Taxane-based [33, 40], one Platinum-based [43], and one Investigator's choice chemotherapy [35]. The other two trials compared PD-1/PD-L1 inhibitors alone to chemotherapy for maintenance therapy in patients with previously treated metastatic TNBC [34, 38]. There were some minor differences in the duration of PD-1/PD-L1 inhibitors used between studies. With the exception of one trial [44], PD-1/PD-L1 inhibitors were used for four to eight cycles with a follow-up time of more than 12 months. The PD-L1 expression in TNBC tissue samples varied significantly between the included RCTs, ranging from 39 to 87% (see Table 1). Table 1 is to be inserted here.

**Table 1:** Characteristics of included trials

Trial	NCT	First author	Year	Phase	No. of TNBC patients	Median age	Cancer stage	ICI used	Treatment arm	Control arm	Dose of ICI used	Duration of ICI used	Median follow-up time	PD-L1 expression positivity
SAFIR02-BREAST [38]	NCT02299999	Bachelot	2021	II	82	56 years	Metastatic treated TNBC for maintenance	Durvalumab	Durvalumab	Continuation of induction Chemotherapy	10 mg/kg q2wk	7 cycles	19.7 months	52%
KEYNOTE-355 [35]	NCT02819518	Cortes	2020	III	847	53.5 years	Untreated locally recurrent inoperable or metastatic TNBC	Pembrolizumab	Pembrolizumab + Investigator’s choice chemotherapy	Placebo + Investigator’s choice chemotherapy	200 mg q3wk	Up to 35 administrations or disease progression	26 months	75%
Garrido-Castro [43]	NCT03414684	Garrido-Castro	2022	II	78	59.1 years	Metastatic TNBC	Nivolumab	Nivolumab + Carboplatin	Carboplatin	360 mg IV q3wk	Not mentioned	23.5 months	39%
NeoTRIPaPDL1 [36]	NCT02620280	Gianni	2022	III	280	50 years	Invasive TNBC	Atezolizumab	Atezolizumab + Carboplatin + Nab-paclitaxel	Carboplatin + Nab-paclitaxel	1200 mg IV q3wk	8 cycles	On-going	56%
GeparNuevo [37]	NCT02685059	Loibl	2022	II	174	49.5 years	Primary, non-metastatic invasive TNBC	Durvalumab	Durvalumab + Nab-paclitaxel then epirubicin/cyclophosphamide	Placebo + Nab-paclitaxel then epirubicin/cyclophosphamide	First dose 0.75 g then 1.5 g q4wk	6 cycles	43.7 months	87%
IMpassion131 [33]	NCT03125902	Miles	2021	III	651	54 years	Unresectable locally or metastatic TNBC	Atezolizumab	Atezolizumab + Paclitaxel	Placebo + Paclitaxel	840 mg day 1,15 q4wk	6 cycles	14.5 months	45%
IMpassion031 [39]	NCT03197935	Mittendorf	2020	III	333	51 years	Early-stage TNBC	Atezolizumab	Atezolizumab + Nab-paclitaxel/Doxorubicin/Cyclophosphamide	Placebo + Nab-paclitaxel + Doxorubicin/Cyclophosphamide	840 mg IV q2wk	6 cycles	20 months	46%
IMpassion130 [40]	NCT02425891	Schmid	2022	III	902	55 years	Advanced TNBC	Atezolizumab	Atezolizumab + Nab-paclitaxel	Placebo + Nab-paclitaxel	840 mg day 1,15 q4wk	6 cycles	12.9 months	41%
Nci 10,013 [44]	NCT02883062	Ademuyiwa	2021	II	67	52 years	Stage II-III TNBC	Atezolizumab	Carboplatin + Paclitaxel	Atezolizumab + Carboplatin + Paclitaxel	1200 mg q3wk	4 cycles	6 months	Not done yet
KEYNOTE-522 [41]	NCT03036488	Schmid	2020	III	1174	48–49 years	Primary, non-metastatic TNBC (Stage II and III)	Pembrolizumab	Pembrolizumab + Paclitaxel/Carboplatin + Doxorubicin OR Epirubicin/Cyclophosphamide	Placebo + Paclitaxel/Carboplatin + Doxorubicin OR Epirubicin/Cyclophosphamide	200 mg IV q3wk	Neoadjuvant up to 8 cycles	15.5 months	84%
KEYNOTE-119 [34]	NCT02555657	Winer	2021	III	622	52 years	Metastatic treated TNBC	Pembrolizumab	Pembrolizumab	Investigator’s choice mono-chemotherapy	200 mg IV q3wk	Up to 35 administrations	31.5 months	65%
I-SPY2 Trial [42]	NCT01042379	Nanda	2020	II	114	50 years	Stage II-III TNBC	Pembrolizumab	Pembrolizumab + Paclitaxel + Doxorubicin/Cyclophosphamide	Paclitaxel + Doxorubicin/Cyclophosphamide	200 mg IV q3wk	4 cycles	3 yrs	Not done yet

*NCT* National Clinical Trial; *TNBC* Triple Negative Breast Cancer; *ICI* Immune Checkpoint Inhibitor; *PD-L1*, Programmed Cell Death Ligand 1; q2wk. once every 2 weeks; q3wk. once every 3 weeks; q4wk. once every 4 weeks

### Risk of bias assessment

**Fig. 2:**
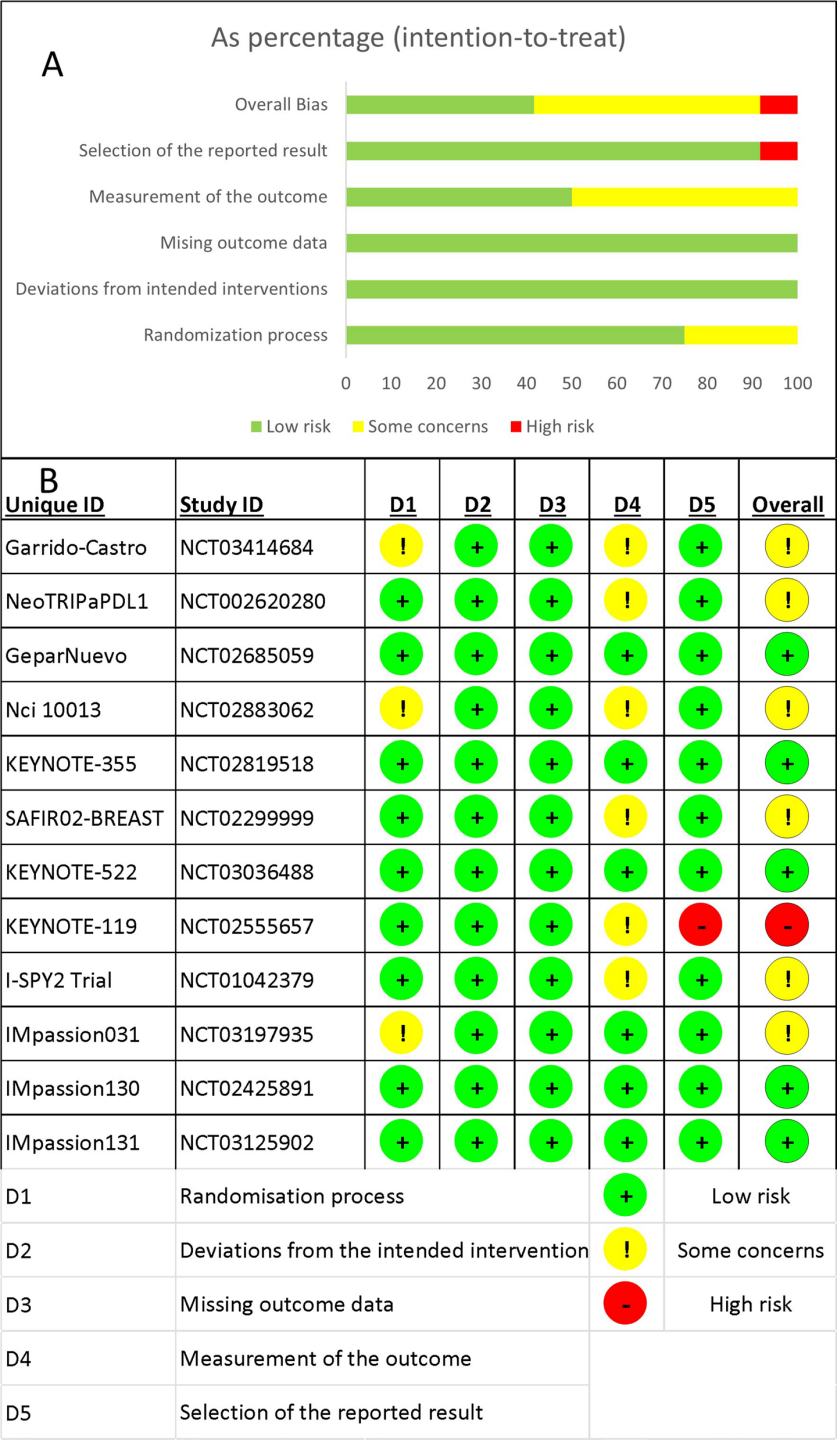
The results of the risk of bias assessment. **A** Stacked bar chart showing a summary of the risk of bias assessment overall and in each domain. **B** The detailed answers for all studies in each domain

Overall, five RCTs had a low risk of bias [33, 35, 37, 40, 41], six had some concerns [36, 38, 39, 42–44], and only one had a high risk of bias [34]. When following the intended protocol and performing ITT analysis, all included trials were of high quality. Five of the six trials that raised concerns were due to the trial being non-blinded [36, 38, 42–44], which could affect the assessment of the outcome of interest. One study found a significant difference in one of the baseline parameters [39], while the high-risk study failed to report one of the secondary outcomes in the main text [34]. Figure 2 depicts the overall risk of bias across all domains (Fig. 2A), as well as the reviewers' judgment within each domain for all included trials (Fig. 2B).

### Overall survival

**Fig. 3:**
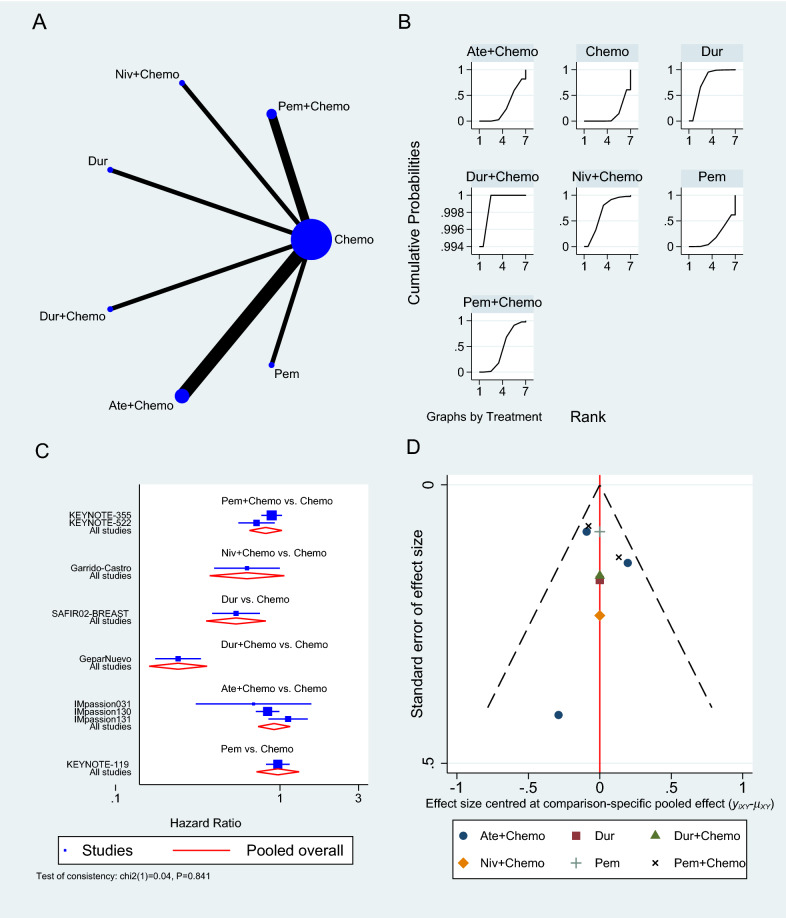
Overall survival network meta-analysis results. **A** Schematic diagram showing the network map for the treatments included in the analysis. **B** Rankogram showing the ranking probabilities for the effectiveness of each treatment. **C** Forest plot showing each trial effect size and confidence interval as well as the pooled effect size. **D** Bias-adjusted funnel plot showing each treatment separately

The OS was reported in nine RCTs [33–35, 37–41, 43], three of which used Atezolizumab [33, 39, 40], two used Pembrolizumab [35, 41], and one used either Durvalumab or Nivolumab as a neoadjuvant to chemotherapy (Fig. 3A) [37, 43]. Pembrolizumab in a neoadjuvant setting had a pooled HR of 0.82 (95% CI 0.65 to 1.03, SUCRA = 46%, n = 2 trials, 1449 patients), which was comparable to Atezolizumab’s HR of 0.92 (95% CI 0.74 to 1.15, SUCRA = 28%, n = 3 studies, 1886 patients), demonstrating a prolonged but insignificant OS in PD-1/PD-L1 inhibitors arms (see SUCRA Additional file 1: Table S3). GeparNuevo using Durvalumab had the only significant reported prolonged OS in PD-1/PD-L1 inhibitors in neoadjuvant settings (HR 0.24, 95% CI 0.08 to 0.72) [37]. Durvalumab also improved OS when used as a monotherapy for maintenance therapy in patients with metastatic TNBC (SAFIR02-BREAST trial, HR 0.54, 95% CI 0.30 to 0.97) [38]. This outcome's results were consistent among the studies. The rest of the analysis is shown in Fig. 3. GPM sensitivity analysis also revealed no significant differences (Additional file 1: Figure S1).

### Progression-free survival

**Fig. 4:**
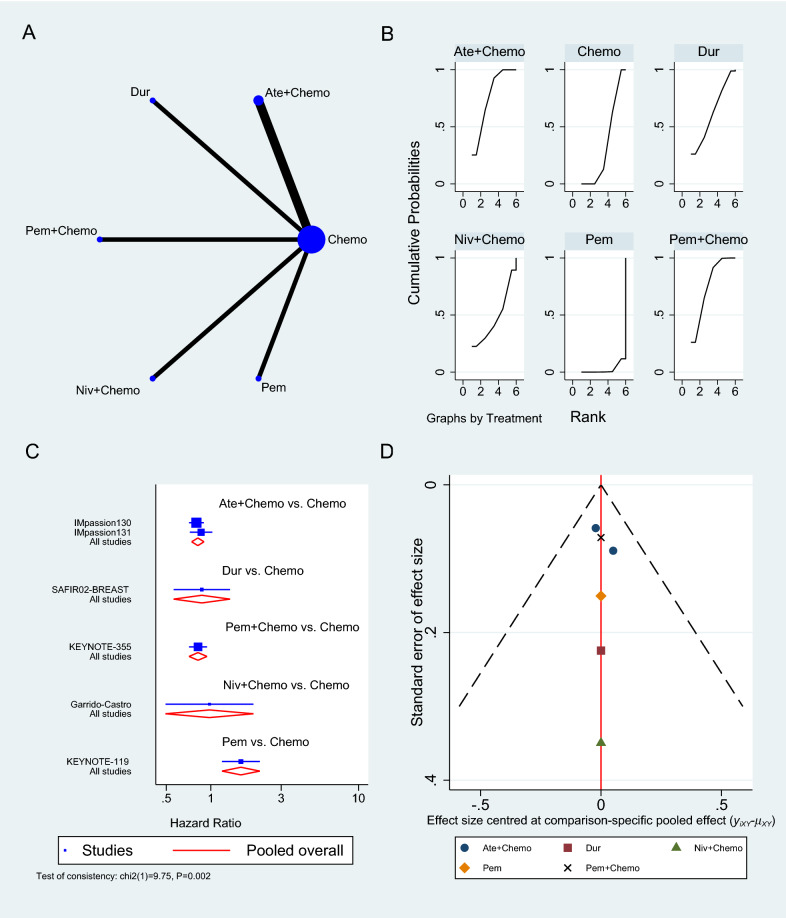
Progression-free survival network meta-analysis results. **A** Schematic diagram showing the network map for the treatments included in the analysis. **B** Rankogram showing the ranking probabilities for the effectiveness of each treatment. **C** Forest plot showing each trial effect size and confidence interval as well as the pooled effect size. **D** Bias-adjusted funnel plot showing each treatment separately

Only six RCTs reported PFS [33–35, 38, 40, 43], two of which used Atezolizumab in neoadjuvant sitting [33, 40], as shown in Fig. 4A. In a neoadjuvant setting along with chemotherapy, Atezolizumab achieved a pooled PFS HR of 0.82 (95% CI 0.69 to 0.97, SUCRA = 76.5%, 1553 patients) (see complete SUCRA values in Additional file 1: Table S4), whereas Pembrolizumab can also prolong PFS as reported in KEYNOTE-355 trial when combined with chemotherapy (HR 0.82, 95% CI 0.71 to 0.94) [35]. In the SAFIR02-BREAST trial, Durvalumab had similar PFS to single-agent chemotherapy (HR 0.87, 95% CI 0.54 to 1.42, 82 patients) [38], whereas Pembrolizumab alone was associated with significantly worse PFS than chemotherapy in KEYNOTE-119 trial (HR 1.60, 95% CI 1.33 to 19.2, 622 patients) [34]. The rest of the analysis is shown in Fig. 4, and the GPM sensitivity analysis is illustrated in the Additional file 1: (Figure S2).

### Pathologic complete response

**Fig. 5:**
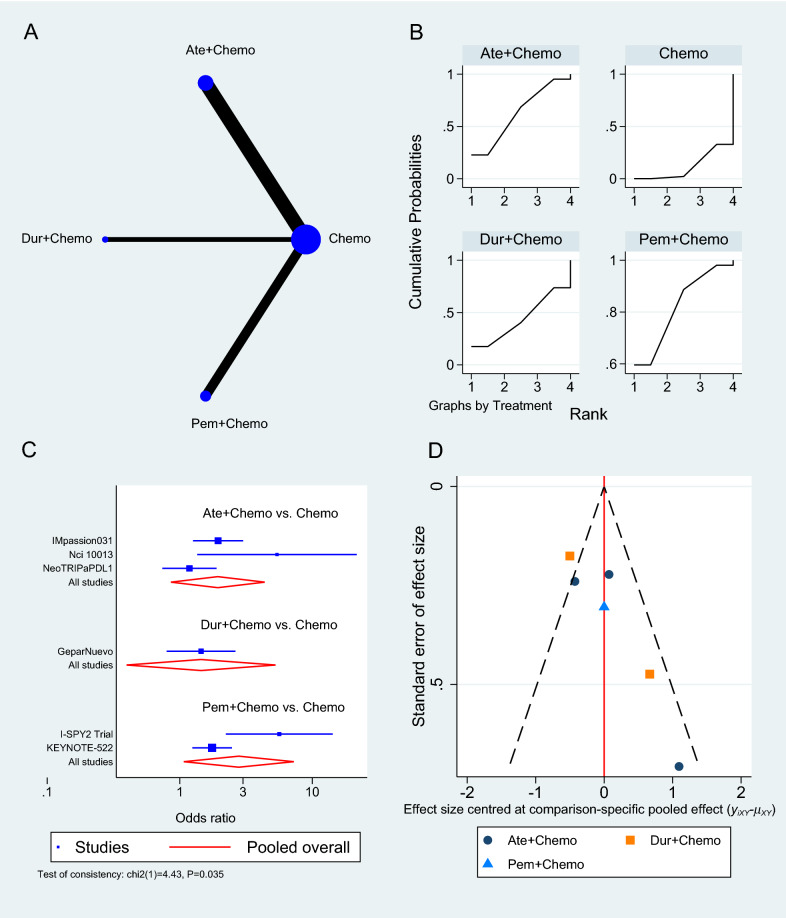
Pathologic complete response network meta-analysis results. **A** Schematic diagram showing the network map for the treatments included in the analysis. **B** Rankogram showing the ranking probabilities for the effectiveness of each treatment. **C** Forest plot showing each trial effect size and confidence interval as well as the pooled effect size. **D** Bias-adjusted funnel plot showing each treatment separately

The number of patients who achieved a complete response was reported in six trials [36, 39, 41, 42, 44]: three on Atezolizumab [36, 39, 44], two on Pembrolizumab [41, 42], and one on Durvalumab [37], all in the neoadjuvant setting to chemotherapy. Pembrolizumab in combination with chemotherapy significantly increased the odds of achieving pCR compared to chemotherapy alone (OR 2.79, 95% CI 1.07 to 7.24, SUCRA = 82.1%, 2 studies, 709 patients), whereas Atezolizumab showed an insignificant increase in pCR (OR 1.94, 95% CI 0.86 to 4.37, SUCRA = 62.3, 3 studies, 674 patients) (complete SUCRA values in Additional file 1: Table S5). In the GeparNuevo trial, the calculated OR of achieving pCR with Durvalumab and chemotherapy was 1.45 (95% CI 0.80 to 2.63) [37]. Figure 5 summarizes the results of the pCR analysis, and the GPM sensitivity analysis is presented in the Additional file 1: Figure S3.

### Adverse events

**Fig. 6:**
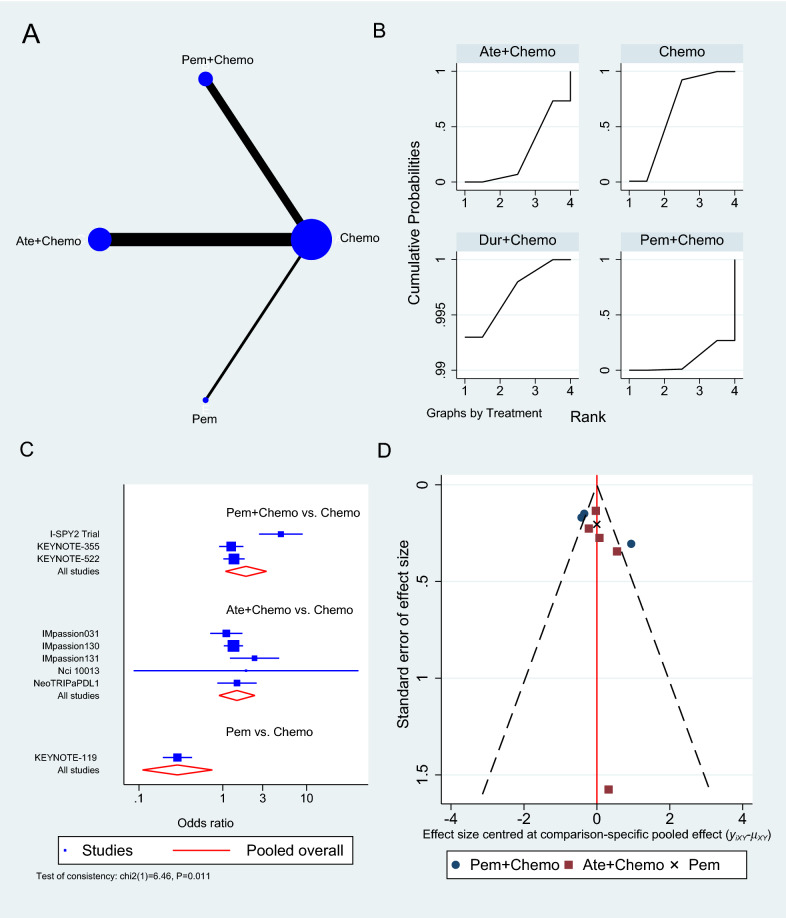
Grade ≥ 3 adverse events network meta-analysis results. **A** Schematic diagram showing the network map for the treatments included in the analysis. **B** Rankogram showing the ranking probabilities for the least odds of causing adverse events for each treatment. **C** Forest plot showing each trial effect size and confidence interval as well as the pooled effect size. **D** Bias-adjusted funnel plot showing each treatment separately

At the time of analysis, nine trials had AEs grade ≥ 3 results reported [33–36, 39–42, 44], the majority of which was the effect of Atezolizumab combined with chemotherapy versus chemotherapy alone (n = 5 studies) [33, 36, 39, 40, 44], followed by Pembrolizumab with chemotherapy (n = 3 studies) (Fig. 6A) [35, 41, 42]. The pooled OR of Atezolizumab addition to chemotherapy causing AEs grade 3 or more compared to chemotherapy alone was 1.48 (95% CI 0.90 to 2.42, 5 studies, 2325 patients), whereas Pembrolizumab with chemotherapy showed a slightly greater risk of causing AEs grade ≥ 3 (OR 1.90, 95% CI 1.08 to 3.33, 3 studies, 2263 patients) (Fig. 6C). Atezolizumab and Pembrolizumab achieved SUCRA values of 26.7% and 9.3% respectively compared to 64.3% for chemotherapy (Additional file 1: Table S6). When compared to single-agent chemotherapy, the KEYNOTE-119 trial showed a significant reduction in AEs grade ≥ 3 when using Pembrolizumab alone in maintenance therapy (OR 0.29, 95% CI 0.19 to 0.43) [34].

In the sensitivity analysis investigating the subtype of the reported AEs, neoadjuvant Pembrolizumab to chemotherapy showed an increase in the odds of developing adrenal insufficiency (OR 26.24, 95% CI 3.50 to 197.86, Additional file 1: Figure S4), diarrhea (OR 1.47, 95% CI 1.14 to 1.88, Additional file 1: Figure S5), hyperthyroidism (OR 5.22, 95% CI 2.44 to 11.15, Additional file 1: Figure S6), hypothyroidism (OR 5.23, 95% CI 3.35 to 8.16, Additional file 1: Figure S7), infusion reaction (OR 1.64, 95% CI 1.13 to 2.37, Additional file 1: Figure S8) and pneumonitis (OR 5.94, 95% CI 1.29 to 27.27, Additional file 1: Figure S9). On the other hand, Atezolizumab in the neoadjuvant settings increased the odds of developing hyperthyroidism (OR 10.91, 95% CI 1.98 to 60.15, Additional file 1: Figure S6), hypothyroidism (OR 3.77, 95% CI 2.52 to 5.63, Additional file 1: Figure S7) and pneumonitis (OR 2.73, 95% CI 1.41 to 5.31, Additional file 1: Figure S9) compared to chemotherapy alone. The remaining results of the sensitivity analysis according to the type of AE developed and GPM are outlined in the Additional file 1: (Figure S10 to Figure S17).

## Discussion

### Principle findings and existing literature

TNBC is an aggressive form of breast cancer that is often associated with poor patient outcomes, largely due to the limited treatment options available [6]. Intensive research efforts have therefore attempted to improve the efficiency of standard-of-care chemotherapy by incorporating immunotherapeutic agents, particularly ICIs, which have emerged as a novel breakthrough in cancer treatment in the past recent years [15]. The present network meta-analysis aimed to compare the published data on the efficacy and safety of ICIs in treating TNBC. Our results showed that antiPD-1/PD-L1 therapies can be used as a neoadjuvant to chemotherapy in the first-line treatment or alone in previously treated TNBC. Multiple RCTs that were conducted on this topic have demonstrated a greater benefit of adding ICIs to chemotherapy in terms of OS, PFS, and pCR [45–49]. As a result, existing meta-analyses evaluating those trials were successful in achieving statistical and clinical significance. For example, Zhang et al. group reported that PD-1/PD-L1 inhibitors in combination with chemotherapy improved pCR (OR 1.59, 95% CI 1.28 to 1.98), event-free survival (HR 0.66, 95% CI 0.48 to 0.91, p = 0.01), and overall survival (HR 0.72, 95% CI 0.52 to 0.99) in TNBC patients compared to chemotherapy alone [45]. Moreover, Li et al. studied the pCR of ICIs in neoadjuvant setting in TNBC and reported that the OR significantly increased in their four included study meta-analysis (OR 2.14, 95% CI 1.37–3.35, P < 0.001) and a better event-free survival (HR 0.66, 95% CI 0.48 to 0.89, P = 0.007) [49], while similar values for pCR were reported by Rizzo et al. (OR 1.95, 95% CI 1.27 to 2.99) and Xin et al. (OR 1.91, 95% CI 1.32 to 2.78) [46, 48]. Villacampa et al. reported that patients with PD-L1-positive tumors had a significantly better PFS with ICIs (HR 0.67, 95% CI 0.58 to 0.79) and a trend towards better OS (HR 0.79, 95% CI 0.60 to 1.03), while no benefit was observed in patients with PD-L1-negative tumors [47]. This is in contrast to Zhang et al. who found that the pCR rate was almost identical in the PD-L1-positive and negative groups [45]. However, many have reported high heterogeneity in effect estimates, indicating major systematic differences between the included RCTs [45–49]. Although this heterogeneity has been attributed to many factors including patient population, TNBC stage, PD-L1 levels, randomization process, and type of chemotherapy regimen, these meta-analyses have failed to acknowledge the performance differences and the distinct immunologic mechanisms by which ICIs act. Contrary to our study, they have combined all agents into a large group and regarded them as one entity, assuming they have similar efficacy and safety.

### Efficacy of PD-1/PD-L1 inhibitors

In our NMA, only two trials out of nine reported statistical significance in terms of OS, both of which used Durvalumab, one as a neoadjuvant (GeparNuevo phase II trial, HR 0.24, 95% CI 0.08 to 0.72, 174 patients) and the other as maintenance (SAFIR02-BREAST trial, HR 0.54, 95% CI 0.30 to 0.97) [37, 38]. Six of the remaining seven trials reported longer, yet statistically insignificant survival. This could be attributed to the small sample size or the lack of follow-up, yet the possibility of Durvalumab having superior efficacy remains, highlighting the need for an additional large phase III RCTs investigating Durvalumab efficacy and safety in TNBC. Five of the seven trials that used neoadjuvant PD-1/PD-L1 inhibitors and reported OS were invasive or metastatic (advanced), with only GeparNuevo achieving a significant reduction in OS HR (0.24, 95% CI 0.08 to 0.72). The remaining two neoadjuvant trials (IMpassion031 trial, HR 0.69, 95% CI 0.25 to 1.87) and (KEYNOTE-522 trial, HR 0.72, 95% CI 0.51 to 1.02) were on non-metastatic or advanced and did not show any improvement in OS.

In general, PFS prolongation followed a positive trend similar to OS when ICIs were used. The IMpassion130 trial demonstrated a significant improvement in PFS with Atezolizumab (HR 0.80, 95% CI 0.69 to 0.92) [40], as opposed to the confirmatory trial Impassion131which failed to achieve statistical significance with Atezolizumab despite extending PFS (HR 0.86, 95% CI 0.70 to 1.05) [33]. An FDA review of the discordant findings between these two trials, including chemotherapy regimens, study design, conduct and population found no single component that could be responsible for this discrepancy, as a result, the reason for this is unclear at present. It is also worth mentioning that the only two trials that reported statistical significance, KEYNOTE-355 and IMpassion130, are the ones with the largest population sample, which may have accounted for their outcome.

Alternatively, the KEYNOTE-355 trial found that Pembrolizumab is effective in prolonging PFS in the neoadjuvant setting (HR 0.82, 95% CI 0.69 to 0.97) [35], while Nivolumab appears to be less effective in improving survival PFS (HR 0.98, 95% CI 0.51 to 1.88). Both KEYNOTE-522 and IMpassion031 trials found that using ICI in the neoadjuvant setting improved disease-free survival [39, 41]. ICIs use as maintenance therapy instead of chemotherapy in treated metastatic TNBC has also shown promising results in terms of prolonging survival using Durvalumab in the SAFIR02-BREAST trial, in contrast to Pembrolizumab that showed no significant improvement in the Keynote-119 trial (PFS HR 1.6, 95% CI 1.33 to 1.92) [34, 38]. Nonetheless, the Keynote-119 trial demonstrated a significant reduction in AEs grade ≥ 3, negating one of chemotherapy's worst attributes [34]. Furthermore, ICIs have also been shown to improve the chances of achieving pCR in TNBC patients when compared to chemotherapy alone. According to our NMA, neoadjuvant Pembrolizumab resulted in the highest pCR (OR 2.79, 95% CI 1.07 to 7.24), followed by Atezolizumab (OR 1.94, 95% CI 0.86 to 4.37, 3 studies, 674 patients), and Durvalumab, which had the lowest pCR (1.45, 95% CI 0.80 to 2.63). However, among the six trials that reported pCR, NeoTRIPaPDL1 and GeparNuevo were the only two RCTs that did not report significant improvement in pCR [36, 37]. This can be explained by the advanced TNBC stage both studies were conducted upon, implying that using ICIs at an earlier stage of TNBC disease progression will more likely benefit patients and improve their survival. This is supported by the fact that early-stage TNBC has a greater tumor immune microenvironment than advanced TNBC, which increases the effectiveness of ICIs with the additional stimulation to the immune response provided by chemotherapy treatment [46]. Another possibility for the negative NeoTRIPaPDL1 results could be due to the insufficient immune induction effect of the chemotherapy regimens used in the study design [46].

### Safety of PD-1/PD-L1 inhibitors

In regard to safety, ICIs appear to be associated with a significant toxicity burden, especially in the form of immune-related AEs [50]. Our NMA showed that Pembrolizumab generally has a worse safety profile than Atezolizumab, causing more grade ≥ 3 AEs (OR 1.90, 95% CI 1.08 to 3.33). Despite the fact that both drugs increased the risk of hyperthyroidism, hypothyroidism, and pneumonitis, Pembrolizumab caused a significant increase in adrenal insufficiency, diarrhea, and infusion reaction, making Atezolizumab a safer option. These AEs are likely to be related to drugs’ mechanism of action. The ability of ICIs to reinvigorate exhausted T-cells in an attempt to kill the tumor may destroy the immune tolerance balance and result in autoimmune and inflammatory responses in normal tissue [51, 52]. However, the reason why certain people or specific organs are more susceptible than others is still incompletely understood [51]. Proposed hypotheses include hereditary predisposition, environmental factors and expression of shared antigens between tumors and affected tissue [51]. Whilst most of these immune-related AEs are usually manageable and reversible, some may require long-term intervention, such as endocrinopathies [50]. Of note, close monitoring of patients and early detection of any AEs is of utmost importance to ensure patients can benefit from adding PD-1/PD-L1 inhibitors to their chemotherapy regimen. Careful follow-up care is also warranted to prevent potential later onset immune-related AEs that may present after cessation of ICIs [50].

### Enhancing the benefit of PD-1/PD-L1 inhibitors

It is crucial to note that the response to ICIs as well as to the combination of other agents differs significantly among patients, highlighting the importance of predictive biomarkers [53]. A multitude of promising novel biomarkers has recently gained considerable attention including the CD274 gene and TILs, but to date, PD-L1 status remains the only biomarker approved to guide patient selection in TNBC [53–55]. We considered PD-L1 positivity as CPS ≥ 1 in Table 1, yet the threshold for PD-L1 positivity and at what level ICIs become more effective remains a topic of scientific debate. Analysis of the present NMA showed that IMpassion031, Keynote-522, and GeparNuevo trials have all demonstrated PD-1/PD-L1 inhibitors to improve efficacy regardless of PD-L1 status in patients with early-stage TNBC [33, 41]. Conversely, IMpassion130 and Keynote-355 demonstrated improved efficacy in metastatic TNBC but not in early-stage TNBC [35, 40]. Following the outcomes of the recently published IMpassion130 and KEYNOTE-355 trials, this biomarker was validated as a predictor of response to PD-1/PD-L1 inhibitors in metastatic breast cancer [48]. Even though data from a previous meta-analysis found no correlation between pCR rates and PD-L1 expression, further investigation revealed pCR rates to be higher in PD-L1-positive patients [46]. Notably, the lack of a standardized approach for PD-L1 detection in TNBC has led to inconsistent PD-L1 prevalence, thereby hampering the precise guiding of immunotherapy [45, 54]. Another significant challenge is that TNBC is composed of numerous heterogeneous subtypes. Biomarker research on IMpassion130 samples revealed that PD-L1 is expressed higher in basal-like immune-activated subtype (75%) and immune-inflamed tumors (63%) TNBC subtypes [56, 57]. Another exploratory study found an improved advantage in PFS in TNBC patients with immune-inflamed tumors, basal-like immune-activated and basal-like immunosuppressed subtypes, in addition to the prolonged OS in inflamed tumors and basal-like immune-activated subtypes [47, 56, 57]. Certainly, the identification of predictive biomarkers of efficacy will greatly aid in optimizing personalized regimens for TNBC patients, as well as predicting the long-term effectiveness of PD-1/PD-L1 inhibitors.

### Future RCTs using PD-1/PD-L1 inhibitors in TNBC

Interestingly, the majority of the currently ongoing RCTs are investigating Atezolizumab and Pembrolizumab, both of which were studied the most in nine out of the 12 RCTs included in our NMA. Hoffmann-La Roche, the sponsor of IMpassion130, IMpassion131, and Impassion031, is currently funding three additional phase III RCTs on Atezolizumab. IMpassion132 is a double-blind Phase III RCT on the efficacy and safety of neoadjuvant Atezolizumab for early relapsing TNBC (NCT03371017), while IMpassion030 is planned to be the largest RCT on ICI as it is presently recruiting 2300 patients with operable TNBC to investigate the combination of neoadjuvant Atezolizumab and chemotherapy (NCT03498716). Hoffmann-La Roche’s third RCT is looking into the combination of Atezolizumab, Ipatasertib, and Paclitaxel in patients with advanced or metastatic TNBC (NCT04177108). In another phase III double-blinded RCT, GeparDouze will investigate neoadjuvant Atezolizumab followed by adjuvant Atezolizumab in patients with high-risk TNBC (NCT03281954). The National Cancer Institute (NCI) is also funding a large phase III RCT to assess the efficacy and safety of Pembrolizumab as adjuvant therapy following neoadjuvant chemotherapy (NCT02954874). Additionally, ASCENT-04 and ASCENT-05 are both ongoing phase III RCTs investigating the PFS of Pembrolizumab in combination with Sacituzumab Govitecan versus chemotherapy in either advanced or residual invasive TNBC (NCT05382286, NCT05633654). TROPION-Breast03 is similarly a new phase III RCT looking at Datopotamab Deruxtecan (DatoDXd) with or without Durvalumab in early-stage TNBC (NCT05629585). Finally, Avelumab, another PD-L1 inhibitor, is currently being studied in a phase III RCT on high-risk TNBC patients (A-Brave trial, NCT02926196).

## Limitations

There are some limitations that must be addressed in this NMA. Firstly, only 12 studies were included, in addition to the limited number of reported outcomes of interest. This is primarily due to the fact that we only included phase II and phase III RCTs because our goal was to compare the efficacy of PD-1/PD-L1 inhibitors in clinical settings. With the ongoing development of neoadjuvant ICI clinical trials, there will certainly be more comprehensive data to be analyzed in future NMA. Second, the NMA comparisons were solely based on direct evidence, with no head-to-head comparisons of neoadjuvant ICIs in TNBC. Moreover, the small number of studies has caused the limited network connectivity to produce large confidence intervals for some estimates, even when effect sizes were large. It may have also resulted in an immature investigation of heterogeneity and publication bias. We would also like to point out the differences between the included studies in terms of TNBC stage, chemotherapy backbone, ICI duration, follow-up time, and PD-L1 expression status. Different chemotherapy backbone regimens used in different studies may have influenced the interpretation of the results as they could have been added to separate groups in the NMA if the number of included studies allowed. Given this heterogeneity and the limited RCTs number, further subgroup analysis based on PD-L1 expression status and nodal involvement, as well as advanced vs early-stage, was not deemed feasible. Finally, all data in this study were derived from published literature, and no individual patient data were used. Noteworthily, the meta-analysis results could potentially be biased by two of the included RCTs that were published as abstracts, which may have relatively incomplete data, missing safety data, and unclear research methods.

## Conclusion

Our NMA found variation in efficacy and safety among PD-1/PD-L1 inhibitors used to treat TNBC, as well as significant systematic differences between the RCTs included. To better assess those variations in efficacy, head-to-head trials between those PD-1/PD-L1 inhibitors are needed. In their use as a neoadjuvant to chemotherapy, ICIs demonstrated comparable efficacy in terms of OS, PFS, and pCR. This benefit is offset by an increase in immune-related adverse events, such as hyperthyroidism, hypothyroidism, pneumonitis, and adrenal insufficiency. We also demonstrated that Atezolizumab is safer than Pembrolizumab in the neoadjuvant setting. Only trials evaluating early-stage TNBC showed a significant improvement in pCR, implying that PD-1/PD-L1 inhibitors may be most effective when started early in the disease course. Durvalumab as a maintenance therapy instead of chemotherapy in patients with metastatic TNBC has also shown promising results in terms of survival extension. Future research should focus on PD-L1 expression status and TNBC subtypes, as these parameters may aid in the optimization of personalized treatment regimens for TNBC patients.

## Supplementary Information

**Additional file 1: Table S1: **PRISMA checklist for this network meta-analysis. **Table S2: **Excluded articles at full-text screening. **Table S3: **Overall survival treatment ranking and surface under the cumulative ranking curve. **Figure S1: **Overall survival using generalized pairwise modelling. **Table S4: **Progression free survival treatment ranking and surface under the cumulative ranking curve. **Figure S2: **Progression free survival using generalized pairwise modelling. **Table S5: **Pathologic complete response treatment ranking and surface under the cumulative ranking curve. **Figure S3: **Pathologic complete response using generalized pairwise modelling. **Table S6: **Adverse events grade ≥ 3 treatment ranking and surface under the cumulative Table. **Figure S4:** Adrenal insufficiency odds network meta-analysis results. **A** Schematic diagram showing the network map for the treatments included in the analysis. **B** Rankogram showing the ranking probabilities for the least odds of causing this adverse event for each treatment. **C **Forest plot showing each trial effect size and confidence interval as well as the pooled effect size. **D **Bias-adjusted funnel plot showing each treatment separately. **Figure S5:** Diarrhea odds network meta-analysis results. **A** Schematic diagram showing the network map for the treatments included in the analysis. **B** Rankogram showing the ranking probabilities for the least odds of causing this adverse event for each treatment. **C **Forest plot showing each trial effect size and confidence interval as well as the pooled effect size. **D **Bias-adjusted funnel plot showing each treatment separately. **Figure S6:** Hyperthyroidism odds network meta-analysis results. **A** Schematic diagram showing the network map for the treatments included in the analysis. **B** Rankogram showing the ranking probabilities for the least odds of causing this adverse event for each treatment. **C **Forest plot showing each trial effect size and confidence interval as well as the pooled effect size. **D **Bias-adjusted funnel plot showing each treatment separately. **Figure S7:** Hypothyroidism odds network meta-analysis results. **A** Schematic diagram showing the network map for the treatments included in the analysis. **B** Rankogram showing the ranking probabilities for the least odds of causing this adverse event for each treatment. **C **Forest plot showing each trial effect size and confidence interval as well as the pooled effect size. **D **Bias-adjusted funnel plot showing each treatment separately. **Figure S8:** Infusion reaction odds network meta-analysis results. **A** Schematic diagram showing the network map for the treatments included in the analysis. **B** Rankogram showing the ranking probabilities for the least odds of causing this adverse event for each treatment. **C **Forest plot showing each trial effect size and confidence interval as well as the pooled effect size. **D **Bias-adjusted funnel plot showing each treatment separately. **Figure S9:** Pneumonitis odds network meta-analysis results. **A** Schematic diagram showing the network map for the treatments included in the analysis. **B** Rankogram showing the ranking probabilities for the least odds of causing this adverse event for each treatment. **C **Forest plot showing each trial effect size and confidence interval as well as the pooled effect size. **D **Bias-adjusted funnel plot showing each treatment separately. **Figure S10:** Anemia odds network meta-analysis results. **A** Schematic diagram showing the network map for the treatments included in the analysis. **B** Rankogram showing the ranking probabilities for the least odds of causing this adverse event for each treatment. **C **Forest plot showing each trial effect size and confidence interval as well as the pooled effect size. **D **Bias-adjusted funnel plot showing each treatment separately. **Figure S11:** Colitis odds network meta-analysis results. **A** Schematic diagram showing the network map for the treatments included in the analysis. **B** Rankogram showing the ranking probabilities for the least odds of causing this adverse event for each treatment. **C **Forest plot showing each trial effect size and confidence interval as well as the pooled effect size. **D **Bias-adjusted funnel plot showing each treatment separately. **Figure S12:** Fatigue odds network meta-analysis results. **A** Schematic diagram showing the network map for the treatments included in the analysis. **B** Rankogram showing the ranking probabilities for the least odds of causing this adverse event for each treatment. **C **Forest plot showing each trial effect size and confidence interval as well as the pooled effect size. **D **Bias-adjusted funnel plot showing each treatment separately. **Figure S13:** Nausea odds network meta-analysis results. **A** Schematic diagram showing the network map for the treatments included in the analysis. **B** Rankogram showing the ranking probabilities for the least odds of causing this adverse event for each treatment. **C **Forest plot showing each trial effect size and confidence interval as well as the pooled effect size. **D **Bias-adjusted funnel plot showing each treatment separately. **Figure S14:** Neutropenia odds network meta-analysis results. **A** Schematic diagram showing the network map for the treatments included in the analysis. **B** Rankogram showing the ranking probabilities for the least odds of causing this adverse event for each treatment. **C **Forest plot showing each trial effect size and confidence interval as well as the pooled effect size. **D **Bias-adjusted funnel plot showing each treatment separately. **Figure S15:** Rash odds network meta-analysis results. **A** Schematic diagram showing the network map for the treatments included in the analysis. **B** Rankogram showing the ranking probabilities for the least odds of causing this adverse event for each treatment. **C **Forest plot showing each trial effect size and confidence interval as well as the pooled effect size. **D **Bias-adjusted funnel plot showing each treatment separately. **Figure S16:** Vomiting odds network meta-analysis results. **A** Schematic diagram showing the network map for the treatments included in the analysis. **B** Rankogram showing the ranking probabilities for the least odds of causing this adverse event for each treatment. **C **Forest plot showing each trial effect size and confidence interval as well as the pooled effect size. **D **Bias-adjusted funnel plot showing each treatment separately. **Figure S17: **Adverse events grade ≥ 3 using generalized pairwise modelling. **Table S7: **Extracted data used for the analysis.

## Data Availability

Data used in this study analysis is provided in the Additional file 1: (Table S7). Further analysis data requests and inquiries can be directed to the corresponding author.

## Abbreviations

AEs: Adverse events
CI: Confidence interval
CPS: Combined positive score
FDA: Food and Drug Administration
GPM: Generalized pairwise modelling
HR: Hazard ratio
ICIs: Immune checkpoint inhibitors
ITT: Intention-to-treat
NMA: Network meta-analysis
OR: Odds ratio
OS: Overall survival
pCR: Pathological complete response
PD-1: Programmed cell death protein 1
PD-L1: Programmed cell death ligand 1
PFS: Progression-free survival
PRISMA: Preferred reporting items for systematic reviews and meta-analyses
PROSPERO: International prospective register of systematic reviews
RoB2: Cochrane risk-of-bias tool 2
SUCRA: Surface under the cumulative ranking curve
TILs: Tumor infiltrating lymphocytes
TNBC: Triple-negative breast cancer
