# Supplementary material for: Comparative efficacy and safety of PD-1/PD-L1 inhibitors in triple negative breast cancer: a systematic review and network meta-analysis of randomized controlled trials
Source: Cancer Cell Int. 2023 May 11;23:90. doi: 10.1186/s12935-023-02941-7 (PMC10173590; doi:10.1186/s12935-023-02941-7)
Supplement: Supplementary file 1 — Additional file 1: Table S1: PRISMA checklist for this network meta-analysis. Table S2: Excluded articles at full-text screening. Table S3: Overall survival treatment ranking and surface under the cumulative ranking curve. Figure S1: Overall survival using generalized pairwise modelling. Table S4: Progression free survival treatment ranking and surface under the cumulative ranking curve. Figure S2: Progression free survival using generalized pairwise modelling. Table S5: Pathologic complete response treatment ranking and surface under the cumulative ranking curve. Figure S3: Pathologic complete response using generalized pairwise modelling. Table S6: Adverse events grade ≥ 3 treatment ranking and surface under the cumulative Table. Figure S4: Adrenal insufficiency odds network meta-analysis results. A Schematic diagram showing the network map for the treatments included in the analysis. B Rankogram showing the ranking probabilities for the least odds of causing this adverse event for each treatment. C Forest plot showing each trial effect size and confidence interval as well as the pooled effect size. D Bias-adjusted funnel plot showing each treatment separately. Figure S5: Diarrhea odds network meta-analysis results. A Schematic diagram showing the network map for the treatments included in the analysis. B Rankogram showing the ranking probabilities for the least odds of causing this adverse event for each treatment. C Forest plot showing each trial effect size and confidence interval as well as the pooled effect size. D Bias-adjusted funnel plot showing each treatment separately. Figure S6: Hyperthyroidism odds network meta-analysis results. A Schematic diagram showing the network map for the treatments included in the analysis. B Rankogram showing the ranking probabilities for the least odds of causing this adverse event for each treatment. C Forest plot showing each trial effect size and confidence interval as well as the pooled effect size. D Bias-adjusted funn [file 12935_2023_2941_MOESM1_ESM.docx]

**Comparative Efficacy and Safety of PD-1/PD-L1 Inhibitors in Triple Negative Breast Cancer: A Systematic Review and Network Meta-analysis of Randomized Controlled Trials**

**Ibrahim Elmakaty, Ruba Abdo, Ahmed Elsabagh, Abdelrahman Elsayed, Mohammed Imad Malki**

**Table of Contents**

**Table S1: Prisma checklist 2**

**Search strategy 6**

**Table S2: Excluded articles at full-text screening 12**

**Table S3: Overall survival treatment ranking and surface under the cumulative ranking curve 17**

**Figure S1: Overall survival using generalized pairwise modelling 18**

**Table S4: Progression free survival treatment ranking and surface under the cumulative ranking curve 18**

**Figure S2: Progression free survival using generalized pairwise modelling18**

**Table S5:Pathologic complete response treatment ranking and surface under the cumulative ranking curve18**

**Figure S3: Pathologic complete response using generalized pairwise modelling 19**

**Table S6: Adverse events grade ≥ 3 treatment ranking and surface under the cumulative Table 19**

**Figure S4: Adrenal insufficiency odds network meta-analysis results. 20**

**Figure S5: Diarrhea odds network meta-analysis results. 21**

**Figure S6: Hyperthyroidism odds network meta-analysis results.22**

**Figure S7: Hypothyroidism odds network meta-analysis results.23**

**Figure S8: Infusion reaction odds network meta-analysis results.24**

**Figure S9: Pneumonitis odds network meta-analysis results.25**

**Figure S10: Anemia odds network meta-analysis results.26**

**Figure S11: Colitis odds network meta-analysis results.27**

**Figure S12: Fatigue odds network meta-analysis results.28**

**Figure S13: Nausea odds network meta-analysis results.29**

**Figure S14: Neutropenia odds network meta-analysis results.30**

**Figure S15: Rash odds network meta-analysis results.31**

**Figure S16: Vomiting odds network meta-analysis results.32**

**Figure S17: Adverse events grade ≥ 3 using generalized pairwise modelling33**

**Table S7: Extracted data used for the analysis 34**

**References 45**

**Table S1:** PRISMA checklist for this network meta-analysis

| **Section/Topic** | **Item #** | **Checklist Item** | **Reported on Page #** |
| --- | --- | --- | --- |
| **TITLE** |  |  |  |
| Title | 1 | Identify the report as a systematic review *incorporating a network meta-analysis (or related form of meta-analysis).* | ***Page 1*** |
|  |  |  |  |
| **ABSTRACT** |  |  | ***Page 2-3*** |
| Structured summary | 2 | Provide a structured summary including, as applicable:  **Background:** main objectives  **Methods:** data sources; study eligibility criteria, participants, and interventions; study appraisal; and *synthesis methods, such as network meta-analysis.*  **Results:** number of studies and participants identified; summary estimates with corresponding confidence/credible intervals; *treatment rankings may also be discussed. Authors may choose to summarize pairwise comparisons against a chosen treatment included in their analyses for brevity.*  **Discussion/Conclusions:** limitations; conclusions and implications of findings.  **Other:** primary source of funding; systematic review registration number with registry name. |  |
|  |  |  |  |
| **INTRODUCTION** |  |  |  |
| Rationale | 3 | Describe the rationale for the review in the context of what is already known*, including mention of why a network meta-analysis has been conducted.* | ***5*** |
| Objectives | 4 | Provide an explicit statement of questions being addressed, with reference to participants, interventions, comparisons, outcomes, and study design (PICOS). | 5 |
|  |  |  |  |
| **METHODS** |  |  |  |
| Protocol and registration | 5 | Indicate whether a review protocol exists and if and where it can be accessed (e.g., Web address); and, if available, provide registration information, including registration number. | 5-6 |
| Eligibility criteria | 6 | Specify study characteristics (e.g., PICOS, length of follow-up) and report characteristics (e.g., years considered, language, publication status) used as criteria for eligibility, giving rationale. *Clearly describe eligible treatments included in the treatment network, and note whether any have been clustered or merged into the same node (with justification).* | ***6-7*** |
| Information sources | 7 | Describe all information sources (e.g., databases with dates of coverage, contact with study authors to identify additional studies) in the search and date last searched. | 6 |
| Search | 8 | Present full electronic search strategy for at least one database, including any limits used, such that it could be repeated. | 6 |
| Study selection | 9 | State the process for selecting studies (i.e., screening, eligibility, included in systematic review, and, if applicable, included in the meta-analysis). | 7 |
| Data collection process | 10 | Describe method of data extraction from reports (e.g., piloted forms, independently, in duplicate) and any processes for obtaining and confirming data from investigators. | 7 |
| Data items | 11 | List and define all variables for which data were sought (e.g., PICOS, funding sources) and any assumptions and simplifications made. | 7 |
| **Geometry of the network** | **S1** | Describe methods used to explore the geometry of the treatment network under study and potential biases related to it. This should include how the evidence base has been graphically summarized for presentation, and what characteristics were compiled and used to describe the evidence base to readers. | ***8-9*** |
| Risk of bias within individual studies | 12 | Describe methods used for assessing risk of bias of individual studies (including specification of whether this was done at the study or outcome level), and how this information is to be used in any data synthesis. | 7-8 |
| Summary measures | 13 | State the principal summary measures (e.g., risk ratio, difference in means). *Also describe the use of additional summary measures assessed, such as treatment rankings and surface under the cumulative ranking curve (SUCRA) values, as well as modified approaches used to present summary findings from meta-analyses.* | 8-10 |
| Planned methods of analysis | 14 | Describe the methods of handling data and combining results of studies for each network meta-analysis. This should include, but not be limited to:   - *Handling of multi-arm trials;* - *Selection of variance structure;* - *Selection of prior distributions in Bayesian analyses; and* - *Assessment of model fit.* | 8-10 |
| **Assessment of Inconsistency** | **S2** | Describe the statistical methods used to evaluate the agreement of direct and indirect evidence in the treatment network(s) studied. Describe efforts taken to address its presence when found. | 8-10 |
| Risk of bias across studies | 15 | Specify any assessment of risk of bias that may affect the cumulative evidence (e.g., publication bias, selective reporting within studies). | **7-8** |
| Additional analyses | 16 | Describe methods of additional analyses if done, indicating which were pre-specified. This may include, but not be limited to, the following:   - Sensitivity or subgroup analyses; - Meta-regression analyses; - *Alternative formulations of the treatment network; and* - *Use of alternative prior distributions for Bayesian analyses (if applicable).* | ***9-10*** |
|  |  |  |  |
| **RESULTS†** |  |  |  |
| Study selection | 17 | Give numbers of studies screened, assessed for eligibility, and included in the review, with reasons for exclusions at each stage, ideally with a flow diagram. | 10 |
| **Presentation of network structure** | **S3** | Provide a network graph of the included studies to enable visualization of the geometry of the treatment network. | ***Figure 3-6*** |
| **Summary of network geometry** | **S4** | Provide a brief overview of characteristics of the treatment network. This may include commentary on the abundance of trials and randomized patients for the different interventions and pairwise comparisons in the network, gaps of evidence in the treatment network, and potential biases reflected by the network structure. | ***10-11*** |
| Study characteristics | 18 | For each study, present characteristics for which data were extracted (e.g., study size, PICOS, follow-up period) and provide the citations. | 10-11 |
| Risk of bias within studies | 19 | Present data on risk of bias of each study and, if available, any outcome level assessment. | 11, Figure 2 |
| Results of individual studies | 20 | For all outcomes considered (benefits or harms), present, for each study: 1) simple summary data for each intervention group, and 2) effect estimates and confidence intervals. *Modified approaches may be needed to deal with information from larger networks.* | ***12-13, Figure 3-6*** |
| Synthesis of results | 21 | Present results of each meta-analysis done, including confidence/credible intervals. *In larger networks, authors may focus on comparisons versus a particular comparator (e.g. placebo or standard care), with full findings presented in an appendix. League tables and forest plots may be considered to summarize pairwise comparisons.* If additional summary measures were explored (such as treatment rankings), these should also be presented. | ***12-13, Figure 3-6*** |
| **Exploration for inconsistency** | **S5** | Describe results from investigations of inconsistency. This may include such information as measures of model fit to compare consistency and inconsistency models, *P* values from statistical tests, or summary of inconsistency estimates from different parts of the treatment network. | ***Not done*** |
| Risk of bias across studies | 22 | Present results of any assessment of risk of bias across studies for the evidence base being studied. | 11, Figure 2 |
| Results of additional analyses | 23 | Give results of additional analyses, if done (e.g., sensitivity or subgroup analyses, meta-regression analyses*, alternative network geometries studied, alternative choice of prior distributions for Bayesian analyses,* and so forth). | ***12-14*** |
|  |  |  |  |
| **DISCUSSION** |  |  |  |
| Summary of evidence | 24 | Summarize the main findings, including the strength of evidence for each main outcome; consider their relevance to key groups (e.g., healthcare providers, users, and policy-makers). | 14 |
| Limitations | 25 | Discuss limitations at study and outcome level (e.g., risk of bias), and at review level (e.g., incomplete retrieval of identified research, reporting bias). *Comment on the validity of the assumptions, such as transitivity and consistency. Comment on any concerns regarding network geometry (e.g., avoidance of certain comparisons).* | 19 |
| Conclusions | 26 | Provide a general interpretation of the results in the context of other evidence, and implications for future research. | 19-20 |
|  |  |  |  |
| **FUNDING** |  |  |  |
| Funding | 27 | Describe sources of funding for the systematic review and other support (e.g., supply of data); role of funders for the systematic review. This should also include information regarding whether funding has been received from manufacturers of treatments in the network and/or whether some of the authors are content experts with professional conflicts of interest that could affect use of treatments in the network. | ***22*** |

**Search strategy**

**PUBMED**

Search Date: 2-11-2022

Number of articles resulted: 202

(“durvalumab”[tiab] OR “durvalumab”[Supplementary Concept] OR “imfinzi”[tiab] OR “MEDI-4736”[tiab] OR “atezolizumab” [tiab] OR “atezolizumab” [Supplementary Concept] OR “tecentriq”[tiab] OR “MPDL3280A” [tiab] OR “RG7446” [tiab] OR “pembrolizumab” [tiab] OR “pembrolizumab”[Supplementary Concept] OR “Keytruda”[tiab] OR “MK-3475” [tiab] OR “SCH-900475” [tiab] OR ”lambrolizumab” [tiab] OR “Nivolumab” [tiab] OR "Nivolumab"[Mesh] OR “Opdivo” [tiab] OR “ONO4538” [tiab] OR “MDX1106” [tiab] OR “BMS936558” [tiab] OR “cemiplimab“ [tiab] OR "cemiplimab" [Supplementary Concept] OR “REGN2810” OR “avelumab” [tiab] OR "avelumab" [Supplementary Concept] OR “MSB0010682” OR “Bavencio” OR “MSB0010718C” [tiab] OR "Immune Checkpoint Inhibitors"[Mesh] OR “programmed cell death 1 receptor/antagonists and inhibitors”[MeSH] OR “immune checkpoint inhibitor*”[tiab] OR “anti-PD-1”[tiab] OR “anti-PD-L1”[tiab] OR “anti-PD1”[tiab] OR “anti-PDL1”[tiab] OR “anti-PD 1”[tiab] OR “anti-PD L1”[tiab] OR “pd1 inhibitor*”[tiab] OR “pdl1 inhibitor*”[tiab] OR “pd 1 inhibitor*”[tiab] OR “pd l1 inhibitor*”[tiab] OR “programmed cell death 1 receptor antagonists and inhibitors”[tiab] OR “programmed cell death 1 receptor antagonist*”[tiab] OR “programmed cell death 1 receptor inhibitor*”[tiab])

AND

("Triple Negative Breast Neoplasms"[MeSH] OR ER-Negative PR-Negative HER2-Negative Breast Neoplasms[tiab] OR "Triple-Negative Breast Cancer"[tiab] OR "Breast Cancer, Triple-Negative"[tiab] OR "Breast Cancers, Triple-Negative"[tiab] OR "Breast Cancer, Triple-Negative"[tiab] OR "Triple-Negative Breast Cancers"[tiab] OR "Triple-Negative Breast Neoplasm"[tiab] OR Breast Neoplasm, Triple-Negative[tiab] OR "Breast Neoplasms, Triple-Negative"[tiab] OR "Triple Negative Breast Neoplasm"[tiab] OR "Triple-Negative Breast Neoplasms"[tiab] OR "ER-Negative PR-Negative HER2-Negative Breast Cancer"[tiab] OR "ER Negative PR Negative HER2 Negative Breast Cancer"[tiab] OR "Triple Negative Breast Cancer"[tiab] OR “Breast Neoplasm"[tiab])

AND

(Randomized controlled trial[pt] OR controlled clinical trial[pt] OR randomized[tiab] OR randomised[tiab] OR placebo[tiab] OR randomly[tiab] OR trial[tiab] OR groups[tiab] OR "Random Allocation"[Mesh] OR "Randomized Controlled Trials as Topic"[Mesh] OR "Randomized Controlled Trial" [Publication Type] )

NOT

(Animals [Mesh] NOT (Animals[Mesh] AND Humans[Mesh]))

**Cochrane Central Register of Controlled Trials (CENTRAL)**

Search Date: 2-11-2022

Number of articles resulted: 309

(durvalumab:ti,ab OR durvalumab:kw OR imfinzi:ti,ab OR MEDI-4736:ti,ab OR atezolizumab:ti,ab OR atezolizumab:kw OR tecentriq:ti,ab OR MPDL3280A:ti,ab OR RG7446:ti,ab OR pembrolizumab:ti,ab OR pembrolizumab:kw OR Keytruda:ti,ab OR MK-3475:ti,ab OR SCH-900475:ti,ab OR lambrolizumab:ti,ab OR Nivolumab:ti,ab OR [mh Nivolumab] OR Opdivo:ti,ab OR ONO4538:ti,ab OR MDX1106:ti,ab OR BMS936558:ti,ab OR cemiplimab:ti,ab OR cemiplimab:kw OR REGN2810 OR avelumab:ti,ab OR avelumab:kw OR MSB0010682 OR Bavencio OR MSB0010718C:ti,ab OR [mh "Immune Checkpoint Inhibitors"] OR ("immune checkpoint" NEXT inhibitor*):ti,ab OR anti-PD-1:ti,ab OR anti-PD-L1:ti,ab OR anti-PD1:ti,ab OR anti-PDL1:ti,ab OR "anti-PD 1":ti,ab OR "anti-PD L1":ti,ab OR ("pd1" NEXT inhibitor*):ti,ab OR ("pdl1" NEXT inhibitor*):ti,ab OR ("pd 1" NEXT inhibitor*):ti,ab OR ("pd l1" NEXT inhibitor*):ti,ab OR "programmed cell death 1 receptor antagonists and inhibitors":ti,ab OR ("programmed cell death 1 receptor" NEXT antagonist*):ti,ab OR ("programmed cell death 1 receptor" NEXT inhibitor*):ti,ab)

AND

([mh "Triple Negative Breast Neoplasms"] OR "ER-Negative PR-Negative HER2-Negative Breast Neoplasms":ti,ab OR "Triple-Negative Breast Cancer":ti,ab OR "Breast Cancer, Triple-Negative":ti,ab OR "Breast Cancers, Triple-Negative":ti,ab OR "Breast Cancer, Triple-Negative":ti,ab OR "Triple-Negative Breast Cancers":ti,ab OR "Triple-Negative Breast Neoplasm":ti,ab OR "Breast Neoplasm, Triple-Negative":ti,ab OR "Breast Neoplasms, Triple-Negative":ti,ab OR "Triple Negative Breast Neoplasm":ti,ab OR "Triple-Negative Breast Neoplasms":ti,ab OR "ER-Negative PR-Negative HER2-Negative Breast Cancer":ti,ab OR "ER Negative PR Negative HER2 Negative Breast Cancer":ti,ab OR "Triple Negative Breast Cancer":ti,ab OR "Breast Neoplasm":ti,ab)

AND

("Randomized controlled trial":pt OR "controlled clinical trial":pt OR randomized:ti,ab OR randomised:ti,ab OR placebo:ti,ab OR randomly:ti,ab OR trial:ti,ab OR groups:ti,ab OR [mh "Random Allocation"] OR [mh "Randomized Controlled Trials as Topic"] OR "Randomized Controlled Trial":pt)

NOT

([mh Animals] NOT ([mh Animals] AND [mh Humans]))

**CINAHL Database**

Search Date: 2-11-2022

Number of articles resulted: 96

((TI durvalumab OR AB durvalumab) OR (MW durvalumab) OR (TI imfinzi OR AB imfinzi) OR (TI MEDI-4736 OR AB MEDI-4736) OR (TI atezolizumab OR AB atezolizumab) OR (MW atezolizumab) OR (TI tecentriq OR AB tecentriq) OR (TI MPDL3280A OR AB MPDL3280A) OR (TI RG7446 OR AB RG7446) OR (TI pembrolizumab OR AB pembrolizumab) OR (MW pembrolizumab) OR (TI Keytruda OR AB Keytruda) OR (TI MK-3475 OR AB MK-3475) OR (TI SCH-900475 OR AB SCH-900475) OR (TI lambrolizumab OR AB lambrolizumab) OR (TI Nivolumab OR AB Nivolumab) OR (MH Nivolumab+) OR (TI Opdivo OR AB Opdivo) OR (TI ONO4538 OR AB ONO4538) OR (TI MDX1106 OR AB MDX1106) OR (TI BMS936558 OR AB BMS936558) OR (TI cemiplimab OR AB cemiplimab) OR (MW cemiplimab) OR REGN2810 OR (TI avelumab OR AB avelumab) OR (MW avelumab) OR MSB0010682 OR Bavencio OR (TI MSB0010718C OR AB MSB0010718C) OR (MH "Immune Checkpoint Inhibitors+") OR (MH "programmed cell death 1 receptor/antagonists and inhibitors+") OR (TI "immune checkpoint inhibitor*" OR AB "immune checkpoint inhibitor*") OR (TI anti-PD-1 OR AB anti-PD-1) OR (TI anti-PD-L1 OR AB anti-PD-L1) OR (TI anti-PD1 OR AB anti-PD1) OR (TI anti-PDL1 OR AB anti-PDL1) OR (TI "anti-PD 1" OR AB "anti-PD 1") OR (TI "anti-PD L1" OR AB "anti-PD L1") OR (TI "pd1 inhibitor*" OR AB "pd1 inhibitor*") OR (TI "pdl1 inhibitor*" OR AB "pdl1 inhibitor*") OR (TI "pd 1 inhibitor*" OR AB "pd 1 inhibitor*") OR (TI "pd l1 inhibitor*" OR AB "pd l1 inhibitor*") OR (TI "programmed cell death 1 receptor antagonists and inhibitors" OR AB "programmed cell death 1 receptor antagonists and inhibitors") OR (TI "programmed cell death 1 receptor antagonist*" OR AB "programmed cell death 1 receptor antagonist*") OR (TI "programmed cell death 1 receptor inhibitor*" OR AB "programmed cell death 1 receptor inhibitor*"))
AND
((MH "Triple Negative Breast Neoplasms+") OR (TI "ER-Negative PR-Negative HER2-Negative Breast Neoplasms" OR AB "ER-Negative PR-Negative HER2-Negative Breast Neoplasms") OR (TI "Triple-Negative Breast Cancer" OR AB "Triple-Negative Breast Cancer") OR (TI "Breast Cancer, Triple-Negative" OR AB "Breast Cancer, Triple-Negative") OR (TI "Breast Cancers, Triple-Negative" OR AB "Breast Cancers, Triple-Negative") OR (TI "Breast Cancer, Triple-Negative" OR AB "Breast Cancer, Triple-Negative") OR (TI "Triple-Negative Breast Cancers" OR AB "Triple-Negative Breast Cancers") OR (TI "Triple-Negative Breast Neoplasm" OR AB "Triple-Negative Breast Neoplasm") OR (TI "Breast Neoplasm, Triple-Negative" OR AB "Breast Neoplasm, Triple-Negative") OR (TI "Breast Neoplasms, Triple-Negative" OR AB "Breast Neoplasms, Triple-Negative") OR (TI "Triple Negative Breast Neoplasm" OR AB "Triple Negative Breast Neoplasm") OR (TI "Triple-Negative Breast Neoplasms" OR AB "Triple-Negative Breast Neoplasms") OR (TI "ER-Negative PR-Negative HER2-Negative Breast Cancer" OR AB "ER-Negative PR-Negative HER2-Negative Breast Cancer") OR (TI "ER Negative PR Negative HER2 Negative Breast Cancer" OR AB "ER Negative PR Negative HER2 Negative Breast Cancer") OR (TI "Triple Negative Breast Cancer" OR AB "Triple Negative Breast Cancer") OR (TI "Breast Neoplasm" OR AB "Breast Neoplasm"))
AND
((PT "Randomized controlled trial") OR (PT "controlled clinical trial") OR (TI randomized OR AB randomized) OR (TI randomised OR AB randomised) OR (TI placebo OR AB placebo) OR (TI randomly OR AB randomly) OR (TI trial OR AB trial) OR (TI groups OR AB groups) OR (MH "Random Allocation+") OR (MH "Randomized Controlled Trials as Topic+") OR (PT "Randomized Controlled Trial"))
NOT
((MH Animals+) NOT ((MH Animals+) AND (MH Humans+)))

**Embase**

Search Date: 2-11-2022

Number of articles resulted: 106

(durvalumab:ti,ab OR durvalumab:tn OR imfinzi:ti,ab OR MEDI-4736:ti,ab OR atezolizumab:ti,ab OR atezolizumab:tn OR tecentriq:ti,ab OR MPDL3280A:ti,ab OR RG7446:ti,ab OR pembrolizumab:ti,ab OR pembrolizumab:tn OR Keytruda:ti,ab OR MK-3475:ti,ab OR SCH-900475:ti,ab OR lambrolizumab:ti,ab OR Nivolumab:ti,ab OR nivolumab/exp OR Opdivo:ti,ab OR ONO4538:ti,ab OR MDX1106:ti,ab OR BMS936558:ti,ab OR cemiplimab:ti,ab OR cemiplimab:tn OR REGN2810 OR avelumab:ti,ab OR avelumab:tn OR MSB0010682 OR Bavencio OR MSB0010718C:ti,ab OR immune checkpoint inhibitor/exp  OR 'immune checkpoint inhibitor*':ti,ab OR anti-PD-1:ti,ab OR anti-PD-L1:ti,ab OR anti-PD1:ti,ab OR anti-PDL1:ti,ab OR 'anti-PD 1':ti,ab OR 'anti-PD L1':ti,ab OR 'pd1 inhibitor*':ti,ab OR 'pdl1 inhibitor*':ti,ab OR 'pd 1 inhibitor*':ti,ab OR 'pd l1 inhibitor*':ti,ab OR 'programmed cell death 1 receptor antagonists and inhibitors':ti,ab OR 'programmed cell death 1 receptor antagonist*':ti,ab OR 'programmed cell death 1 receptor inhibitor*':ti,ab)
AND
(triple negative breast cancer/exp OR 'ER-Negative PR-Negative HER2-Negative Breast Neoplasms':ti,ab OR 'Triple-Negative Breast Cancer':ti,ab OR 'Breast Cancer, Triple-Negative':ti,ab OR 'Breast Cancers, Triple-Negative':ti,ab OR 'Breast Cancer, Triple-Negative':ti,ab OR 'Triple-Negative Breast Cancers':ti,ab OR 'Triple-Negative Breast Neoplasm':ti,ab OR 'Breast Neoplasm, Triple-Negative':ti,ab OR 'Breast Neoplasms, Triple-Negative':ti,ab OR 'Triple Negative Breast Neoplasm':ti,ab OR 'Triple-Negative Breast Neoplasms':ti,ab OR 'ER-Negative PR-Negative HER2-Negative Breast Cancer':ti,ab OR 'ER Negative PR Negative HER2 Negative Breast Cancer':ti,ab OR 'Triple Negative Breast Cancer':ti,ab OR 'Breast Neoplasm':ti,ab)
AND
(term:it OR term:it OR randomized:ti,ab OR randomised:ti,ab OR placebo:ti,ab OR randomly:ti,ab OR trial:ti,ab OR groups:ti,ab OR randomization/exp OR randomized controlled trial (topic)/exp OR term:it)
NOT
(animal/exp NOT (animal/exp AND human/exp))

**Scopus**

Search Date: 2-11-2022

Number of articles resulted: 341

(TITLE-ABS(durvalumab) OR CHEM(term) OR TITLE-ABS(imfinzi) OR TITLE-ABS(MEDI-4736) OR TITLE-ABS(atezolizumab) OR CHEM(term) OR TITLE-ABS(tecentriq) OR TITLE-ABS(MPDL3280A) OR TITLE-ABS(RG7446) OR TITLE-ABS(pembrolizumab) OR CHEM(term) OR TITLE-ABS(Keytruda) OR TITLE-ABS(MK-3475) OR TITLE-ABS(SCH-900475) OR TITLE-ABS(lambrolizumab) OR TITLE-ABS(Nivolumab) OR INDEXTERMS(Nivolumab) OR TITLE-ABS(Opdivo) OR TITLE-ABS(ONO4538) OR TITLE-ABS(MDX1106) OR TITLE-ABS(BMS936558) OR TITLE-ABS(cemiplimab) OR CHEM(term) OR REGN2810 OR TITLE-ABS(avelumab) OR CHEM(term) OR MSB0010682 OR Bavencio OR TITLE-ABS(MSB0010718C) OR INDEXTERMS("Immune Checkpoint Inhibitors") OR INDEXTERMS("programmed cell death 1 receptor/antagonists and inhibitors") OR TITLE-ABS("immune checkpoint inhibitor*") OR TITLE-ABS(anti-PD-1) OR TITLE-ABS(anti-PD-L1) OR TITLE-ABS(anti-PD1) OR TITLE-ABS(anti-PDL1) OR TITLE-ABS("anti-PD 1") OR TITLE-ABS("anti-PD L1") OR TITLE-ABS("pd1 inhibitor*") OR TITLE-ABS("pdl1 inhibitor*") OR TITLE-ABS("pd 1 inhibitor*") OR TITLE-ABS("pd l1 inhibitor*") OR TITLE-ABS("programmed cell death 1 receptor antagonists and inhibitors") OR TITLE-ABS("programmed cell death 1 receptor antagonist*") OR TITLE-ABS("programmed cell death 1 receptor inhibitor*"))

AND

(INDEXTERMS("Triple Negative Breast Neoplasms") OR TITLE-ABS("ER-Negative PR-Negative HER2-Negative Breast Neoplasms") OR TITLE-ABS("Triple-Negative Breast Cancer") OR TITLE-ABS("Breast Cancer, Triple-Negative") OR TITLE-ABS("Breast Cancers, Triple-Negative") OR TITLE-ABS("Breast Cancer, Triple-Negative") OR TITLE-ABS("Triple-Negative Breast Cancers") OR TITLE-ABS("Triple-Negative Breast Neoplasm") OR TITLE-ABS("Breast Neoplasm, Triple-Negative") OR TITLE-ABS("Breast Neoplasms, Triple-Negative") OR TITLE-ABS("Triple Negative Breast Neoplasm") OR TITLE-ABS("Triple-Negative Breast Neoplasms") OR TITLE-ABS("ER-Negative PR-Negative HER2-Negative Breast Cancer") OR TITLE-ABS("ER Negative PR Negative HER2 Negative Breast Cancer") OR TITLE-ABS("Triple Negative Breast Cancer") OR TITLE-ABS("Breast Neoplasm"))

AND

(DOCTYPE("Randomized controlled trial") OR DOCTYPE("controlled clinical trial") OR TITLE-ABS(randomized) OR TITLE-ABS(randomised) OR TITLE-ABS(placebo) OR TITLE-ABS(randomly) OR TITLE-ABS(trial) OR TITLE-ABS(groups) OR INDEXTERMS("Random Allocation") OR INDEXTERMS("Randomized Controlled Trials as Topic") OR DOCTYPE("Randomized Controlled Trial"))

AND NOT

(INDEXTERMS(Animals) AND NOT (INDEXTERMS(Animals) AND INDEXTERMS(Humans)))

**Web of science**

Search Date: 2-11-2022

Number of articles resulted: 422

((TI=durvalumab OR AB=durvalumab) OR ALL=durvalumab OR (TI=imfinzi OR AB=imfinzi) OR (TI=MEDI-4736 OR AB=MEDI-4736) OR (TI=atezolizumab OR AB=atezolizumab) OR ALL=atezolizumab OR (TI=tecentriq OR AB=tecentriq) OR (TI=MPDL3280A OR AB=MPDL3280A) OR (TI=RG7446 OR AB=RG7446) OR (TI=pembrolizumab OR AB=pembrolizumab) OR ALL=pembrolizumab OR (TI=Keytruda OR AB=Keytruda) OR (TI=MK-3475 OR AB=MK-3475) OR (TI=SCH-900475 OR AB=SCH-900475) OR (TI=lambrolizumab OR AB=lambrolizumab) OR (TI=Nivolumab OR AB=Nivolumab) OR ALL=Nivolumab OR (TI=Opdivo OR AB=Opdivo) OR (TI=ONO4538 OR AB=ONO4538) OR (TI=MDX1106 OR AB=MDX1106) OR (TI=BMS936558 OR AB=BMS936558) OR (TI=cemiplimab OR AB=cemiplimab) OR ALL=cemiplimab OR ALL=REGN2810 OR (TI=avelumab OR AB=avelumab) OR ALL=avelumab OR ALL=MSB0010682 OR ALL=Bavencio OR (TI=MSB0010718C OR AB=MSB0010718C) OR ALL="Immune Checkpoint Inhibitors" OR ALL="programmed cell death 1 receptor/antagonists and inhibitors" OR (TI="immune checkpoint inhibitor*" OR AB="immune checkpoint inhibitor*") OR (TI=anti-PD-1 OR AB=anti-PD-1) OR (TI=anti-PD-L1 OR AB=anti-PD-L1) OR (TI=anti-PD1 OR AB=anti-PD1) OR (TI=anti-PDL1 OR AB=anti-PDL1) OR (TI="anti-PD 1" OR AB="anti-PD 1") OR (TI="anti-PD L1" OR AB="anti-PD L1") OR (TI="pd1 inhibitor*" OR AB="pd1 inhibitor*") OR (TI="pdl1 inhibitor*" OR AB="pdl1 inhibitor*") OR (TI="pd 1 inhibitor*" OR AB="pd 1 inhibitor*") OR (TI="pd l1 inhibitor*" OR AB="pd l1 inhibitor*") OR (TI="programmed cell death 1 receptor antagonists and inhibitors" OR AB="programmed cell death 1 receptor antagonists and inhibitors") OR (TI="programmed cell death 1 receptor antagonist*" OR AB="programmed cell death 1 receptor antagonist*") OR (TI="programmed cell death 1 receptor inhibitor*" OR AB="programmed cell death 1 receptor inhibitor*"))

AND
(ALL="Triple Negative Breast Neoplasms" OR (TI="ER-Negative PR-Negative HER2-Negative Breast Neoplasms" OR AB="ER-Negative PR-Negative HER2-Negative Breast Neoplasms") OR (TI="Triple-Negative Breast Cancer" OR AB="Triple-Negative Breast Cancer") OR (TI="Breast Cancer, Triple-Negative" OR AB="Breast Cancer, Triple-Negative") OR (TI="Breast Cancers, Triple-Negative" OR AB="Breast Cancers, Triple-Negative") OR (TI="Breast Cancer, Triple-Negative" OR AB="Breast Cancer, Triple-Negative") OR (TI="Triple-Negative Breast Cancers" OR AB="Triple-Negative Breast Cancers") OR (TI="Triple-Negative Breast Neoplasm" OR AB="Triple-Negative Breast Neoplasm") OR (TI="Breast Neoplasm, Triple-Negative" OR AB="Breast Neoplasm, Triple-Negative") OR (TI="Breast Neoplasms, Triple-Negative" OR AB="Breast Neoplasms, Triple-Negative") OR (TI="Triple Negative Breast Neoplasm" OR AB="Triple Negative Breast Neoplasm") OR (TI="Triple-Negative Breast Neoplasms" OR AB="Triple-Negative Breast Neoplasms") OR (TI="ER-Negative PR-Negative HER2-Negative Breast Cancer" OR AB="ER-Negative PR-Negative HER2-Negative Breast Cancer") OR (TI="ER Negative PR Negative HER2 Negative Breast Cancer" OR AB="ER Negative PR Negative HER2 Negative Breast Cancer") OR (TI="Triple Negative Breast Cancer" OR AB="Triple Negative Breast Cancer") OR (TI="Breast Neoplasm" OR AB="Breast Neoplasm"))
AND
(ALL="Randomized controlled trial" OR ALL="controlled clinical trial" OR (TI=randomized OR AB=randomized) OR (TI=randomised OR AB=randomised) OR (TI=placebo OR AB=placebo) OR (TI=randomly OR AB=randomly) OR (TI=trial OR AB=trial) OR (TI=groups OR AB=groups) OR ALL="Random Allocation" OR ALL="Randomized Controlled Trials as Topic" OR ALL="Randomized Controlled Trial")
NOT
(ALL=Animals NOT (ALL=Animals AND ALL=Humans))

**clinicaltrials.gov**

Search Date: 15-11-2022

Number of articles resulted: 25

(pembrolizumab OR nivolumab OR cemiplimab OR atezolizumab OR avelumab OR durvalumab) AND triple negative breast cancer

Note: filtered to studies with results

**EU Clinical Trials Register**

Search Date: 15-11-2022

Number of articles resulted: 15

(pembrolizumab OR nivolumab OR cemiplimab OR atezolizumab OR avelumab OR durvalumab) AND triple negative breast cancer

Note: filtered to studies with results

**ISRCTN**

Search Date: 15-11-2022

Number of articles resulted: 4

(pembrolizumab OR nivolumab OR cemiplimab OR atezolizumab OR avelumab OR durvalumab) AND triple negative breast cancer

**International Clinical Trials Registry Platform (ICTRP)**

Search Date: 15-11-2022

Number of articles resulted: 19

(pembrolizumab OR nivolumab OR cemiplimab OR atezolizumab OR avelumab OR durvalumab) AND triple negative breast cancer

Note: filtered to studies with results

**breastcancertrials.org**

Search date 23/11/2022

Number of articles resulted : 44

pembrolizumab OR nivolumab OR cemiplimab OR atezolizumab OR avelumab OR durvalumab

Note: filtered to triple negative breast cancer studies

**Table S2:** Excluded articles at full-text screening

| **Article reference** | **Title** | **Reason for exclusion** |
| --- | --- | --- |
| NCT02849496 [1] | Veliparib and Atezolizumab Either Alone or in Combination in Treating Patients With Stage III-IV Triple Negative Breast Cancer | No results yet |
| NCT01898117 [2] | Triple-B Study;Carboplatin-cyclophosphamide Versus Paclitaxel With or Without Atezolizumab as First-line Treatment in Advanced Triple Negative Breast Cancer | No results yet |
| EUCTR2014-005490-37 [3] | A trial of MPDL3280A and nab-Paclitaxel in metastatic triple negative breast cancer | Duplicate |
| (Vidula, et al. 2021) [4] | Translational breast cancer researchconsortium 044 trial: randomized phase 2 study of pembrolizumab and carboplatin versus carboplatin alonefor chest wall recurrence of breast cancer | Duplicate |
| (Saji, et al. 2022) [5] | Subgroup analysis of Japanese patients in a phase III randomized, controlled study of neoadjuvant atezolizumab or placebo, combined with nab-paclitaxel and anthracycline-based chemotherapy in early triple-negative breast cancer (IMpassion031) | Duplicate dataset |
| NCT03197935 [6] | A Study to Investigate Atezolizumab and Chemotherapy Compared With Placebo and Chemotherapy in the Neoadjuvant Setting in Participants With Early Stage Triple Negative Breast Cancer | Duplicate dataset |
| (Rpcec 2018) [7] | A Study of the Efficacy and Safety of Atezolizumab Plus Chemotherapy for Patients With Early Relapsing Recurrent Triple-Negative Breast Cancer | Duplicate dataset |
| NCT02555657 [8] | Study of Single Agent Pembrolizumab (MK-3475) Versus Single Agent Chemotherapy for Metastatic Triple Negative Breast Cancer (MK-3475-119/KEYNOTE-119) | Duplicate dataset |
| NCT02819518 [9] | Study of Pembrolizumab (MK-3475) Plus Chemotherapy vs. Placebo Plus Chemotherapy for Previously Untreated Locally Recurrent Inoperable or Metastatic Triple Negative Breast Cancer (MK-3475-355/KEYNOTE-355) | Duplicate dataset |
| NCT03036488 [10] | Study of Pembrolizumab (MK-3475) Plus Chemotherapy vs Placebo Plus Chemotherapy as Neoadjuvant Therapy and Pembrolizumab vs Placebo as Adjuvant Therapy in Participants With Triple Negative Breast Cancer (TNBC) (MK-3475-522/KEYNOTE-522) | Duplicate |
| NCT02322814 [11] | A Study of Cobimetinib Plus Paclitaxel, Cobimetinib Plus Atezolizumab Plus Paclitaxel, or Cobimetinib Plus Atezolizumab Plus Nab-Paclitaxel as Initial Treatment for Participants With Triple-Negative Breast Cancer That Has Spread | Duplicate |
| Euctr2016-004734-22 [12] | A Study of Atezolizumab with Neoadjuvant Anthracycline/Nab-Paclitaxel Chemotherapy Compared with Placebo and Chemotherapy in Patients with Early Triple-Negative Breast Cancer | Duplicate dataset |
| NCT02425891 [13] | A Study of Atezolizumab in Combination With Nab-Paclitaxel Compared With Placebo With Nab-Paclitaxel for Participants With Previously Untreated Metastatic Triple-Negative Breast Cancer (IMpassion130) | Duplicate dataset |
| CTRI/2017/10/010010 [14] | A Study of Atezolizumab and Paclitaxel Versus Placebo and Paclitaxel in Participants With Previously Untreated Locally Advanced or Metastatic Triple Negative Breast Cancer (TNBC) (IMpassion131) | Duplicate dataset |
| NCT03125902 [15] | A Study of Atezolizumab and Paclitaxel Versus Placebo and Paclitaxel in Participants With Previously Untreated Locally Advanced or Metastatic Triple Negative Breast Cancer (TNBC) | Duplicate dataset |
| EUCTR2016-004024-29 [16] | A Study of Atezolizumab (Anti-PD-L1 Antibody) in Combination with Paclitaxel Compared with Placebo with Paclitaxel for Patients with Previously Untreated Inoperable Locally Advanced or Metastatic Triple Negative Breast Cancer | Duplicate dataset |
| NCT04468061 [17] | Sacituzumab Govitecan +/- Pembrolizumab in Metastatic TNBC | No results yet |
| (Park, et al. 2020) [18] | Randomized, phase II trial to evaluate the efficacy and safety of atezolizumab plus capecitabine adjuvant therapy compared to capecitabine monotherapy for triple receptor-negative breast cancer (TNBC) with residual invasive cancer after neoadjuvant chemotherapy (MIRINAE trial, KCSG-BR18-21) | No results yet |
| (Loibl, et al. 2020) [19] | A randomized, double-blind, phase III trial of neoadjuvant chemotherapy (NACT) with atezolizumab/placebo in patients with triple-negative breast cancer (TNBC) followed by adjuvant continuation of atezolizumab/placebo (Gepardouze) | No results yet |
| EUCTR2016-000189-45 [20] | Randomized trial that compares, with the clinical practice monitoring, an adjuvant or neoadjuvant treatment with an anti PD L1 antibody in breast cancer patients with negative receptors | No results yet |
| (Vidula, et al. 2022) [21] | Randomized phase II trial of pembrolizumab/carboplatin vs. carboplatin alone for breast cancer with chest wall recurrence: TBCRC044 | No results yet |
| (Garrido-Castro, et al. 2018) [22] | A randomized phase II trial of carboplatin with or without nivolumab in first- or secondline metastatic TNBC | Duplicate |
| (Loibl, et al. 2016) [23] | A randomized phase II study to investigate the addition of PD-L1 antibody MEDI4673 (durvalumab) to a taxane-anthracycline containing chemotherapy in triple negative breast cancer (GeparNuevo) | Duplicate |
| (McArthur, et al. 2019b) [24] | A randomized phase II study of peri-operative ipilimumab, nivolumab and cryoablation versus standard peri-operative care in women with residual triple negative early stage/resectable breast cancer after standard-of-care neoadjuvant chemotherapy | No results yet |
| (McArthur, et al. 2019a) [25] | A randomized phase II study of peri-operative ipilimumab, nivolumab and cryoablation versus standard care in women with residual, early stage/resectable, triple negative breast cancer after standard-of-care neoadjuvant chemotherapy | Duplicate |
| (Vidula, et al. 2019) [26] | A randomized phase II study of pembrolizumab in combination with carboplatin versus carboplatin alone in breast cancer patients with chest wall disease, with immunologic and genomic correlative studies | Duplicate |
| (Loibl, et al. 2018) [27] | Randomized phase II neoadjuvant study (GeparNuevo) to investigate the addition of durvalumab to a taxane-anthracycline containing chemotherapy in triple negative breast cancer (TNBC) | Duplicate |
| PER-066-15 [28] | A RANDOMIZED OPEN-LABEL PHASE III STUDY OF SINGLE AGENT PEMBROLIZUMAB VERSUS SINGLE AGENT CHEMOTHERAPY PER PHYSICIAN’S CHOICE FOR METASTATIC TRIPLE NEGATIVE BREAST CANCER (MTNBC) – (KEYNOTE-119) | Duplicate dataset |
| (Geyer, et al. 2019b) [29] | A randomized double-blind phase III clinical trial of neoadjuvant chemotherapy (NAC) with atezolizumab or placebo in patients (pts) with triple negative breast cancer (TNBC) followed by adjuvant atezolizumab or placebo: NSABP B-59/GBG 96-GeparDouze | Duplicate dataset |
| (Loibl, et al. 2019b) [30] | A randomised phase II study investigating durvalumab in addition to an anthracycline taxane-based neoadjuvant therapy in early triple-negative breast cancer: Clinical results and biomarker analysis of GeparNuevo study | Duplicate dataset |
| (Takano, et al. 2022a) [31] | PS2-2 KEYNOTE-355 Asian subset: pembrolizumab + chemotherapy vs placebo + chemotherapy for triple-negative breast cancer | Duplicate dataset |
| (Turner, et al. 2022) [32] | Primary results of the cTRAK TN trial: a clinical trial utilising ctDNA mutation tracking to detect minimal residual disease and trigger intervention in patients with moderate and high risk early stage triple negative breast cancer | Wrong outcome |
| (Sinn, et al. 2019) [33] | Pre-therapeutic PD-L1 expression and dynamics of Ki-67 and gene expression during neoadjuvant immune-checkpoint blockade and chemotherapy to predict response within the GeparNuevo trial | Wrong outcome |
| (Emens, et al. 2016b) [34] | A phase Ill randomized trial of atezolizumab in combination with nab-paclitaxel as first line therapy for patients with metastatic triple-negative breast cancer (mTNBC) | Duplicate |
| EUCTR2016-004740-11 [35] | A Phase III, Randomized, Double-blind Study to Evaluate Chemotherapy plus Pembrolizumab vs Chemotherapy plus Placebo as Neoadjuvant Therapy and Pembrolizumab vs Placebo as Adjuvant Therapy for Triple Negative Breast Cancer (TNBC) | No results yet |
| PER-055-18 [36] | A PHASE III, MULTICENTER, RANDOMIZED, OPEN-LABEL STUDY COMPARING ATEZOLIZUMAB (ANTI-PD-L1 ANTIBODY) IN COMBINATION WITH ADJUVANT ANTHRACYCLINE/TAXANE BASED CHEMOTHERAPY VERSUS CHEMOTHERAPY ALONE IN PATIENTS WITH OPERABLE TRIPLE NEGATIVE BREAST CANCER | No results yet |
| PER-018-19 [37] | A PHASE III, DOUBLE-BLIND, PLACEBO-CONTROLLED, RANDOMIZED STUDY OF IPATASERTIB IN COMBINATION WITH ATEZOLIZUMAB AND PACLITAXEL AS A TREATMENT FOR PATIENTS WITH LOCALLY ADVANCED UNRESECTABLE OR METASTATIC TRIPLE-NEGATIVE BREAST CANCER | No results yet |
| EUCTR2016-001432-35 [38] | A Phase III Study to Evaluate Chemotherapy With or Without Pembrolizumab as First Line Treatment for Triple Negative Breast Cancer | No results yet |
| (Conte, et al. 2020) [39] | Phase III randomized study of adjuvant treatment with the ANTIPD-L1 antibody avelumab for high-risk triple negative breast cancer patients: the A-BRAVE trial | No results yet |
| (Yusof, et al. 2020b) [40] | Phase III KEYNOTE-355 study of pembrolizumab (pembro) vs placebo (pbo) plus chemotherapy (chemo) for previously untreated locally recurrent inoperable or metastatic triple-negative breast cancer (TNBC): Results for patients (Pts) enrolled in Asia | Duplicate dataset |
| (Lander, et al. 2020) [41] | A phase II trial of atezolizumab (anti-PD-L1) with carboplatin in patients with metastatic triplenegative breast cancer (mTNBC) | No results yet |
| EUCTR2015-001969-49 [42] | Phase II study with nivolumab (anti-PD1) in patients with triple negative breast cancer after induction treatment | No results yet |
| (Shah, et al. 2020) [43] | Phase II study of pembrolizumab and capecitabine for triple negative and hormone receptor-positive, HER2-negative endocrine-refractory metastatic breast cancer | Single arm |
| (Obeid, et al. 2017) [44] | A Phase II randomized trial of pembrolizumab with carboplatin and gemcitabine for treatment of patients with metastatic triple-negative breast cancer (mTNBC) | No results yet |
| (Brufsky, et al. 2019) [45] | Phase II COLET study: atezolizumab (A) + cobimetinib (C) + paclitaxel (P)/nab-paclitaxel (nP) as first-line (1L) treatment (tx) for patients (pts) with locally advanced or metastatic triple-negative breast cancer (mTNBC) | Duplicate |
| (Yuan, et al. 2018) [46] | A phase II clinical trial of the combination of pembrolizumab and selective androgen receptor modulator GTx-024 in patients with advanced androgen receptor positive triple negative breast cancer | Duplicate |
| (Yuan, et al. 2021) [47] | A Phase II Clinical Trial of Pembrolizumab and Enobosarm in Patients with Androgen Receptor-Positive Metastatic Triple-Negative Breast Cancer | Single arm |
| (Devaux, et al. 2018) [48] | A phase Ib/II study of durvalumab combined with dose-dense EC in neoadjuvant setting for patients with locally advanced luminal B HER2(-) or triple negative breast cancers (B-IMMUNE) | No results yet |
| (Sun, et al. 2022) [49] | A Phase 2 Trial of Enhancing Immune Checkpoint Blockade by Stereotactic Radiation and In Situ Virus Gene Therapy in Metastatic Triple-Negative Breast Cancer | Single arm |
| NCT04373031 [50] | Pembrolizumab, IRX-2, and Chemotherapy in Triple Negative Breast Cancer | No results yet |
| (Kummel, et al. 2022) [51] | Pembrolizumab vs placebo + chemotherapy as neoadjuvant treatment, followed by pembrolizumab vs placebo as adjuvant treatment for early triple-negative breast cancer (TNBC): phase 3 KEYNOTE-522 study | Duplicate dataset |
| (Winer, et al. 2021) [52] | Pembrolizumab versus chemotherapyfor previously treated metastatic triple-negative breastcancer (KEYNOTE-119): efficacy in patients with lung orliver metastases | Duplicate dataset |
| (Isaac 2020) [53] | Pembrolizumab Plus Neoadjuvant Chemotherapy Improves Pathologic Complete Response Rates in Triple-Negative Breast Cancer | Duplicate dataset |
| (Cortes, et al. 2022b) [54] | Pembrolizumab plus Chemotherapy in Advanced Triple-Negative Breast Cancer | Duplicate dataset |
| (Schmid, et al. 2020c) [55] | Pembrolizumab plus chemotherapy as neoadjuvant treatment of high-risk, early-stage triple-negative breast cancer: results from the phase 1b open-label, multicohort KEYNOTE-173 study | Phase I |
| NCT02954874 [56] | Pembrolizumab in Treating Patients With Triple-Negative Breast Cancer | No results yet |
| (Sidaway 2022) [57] | Pembrolizumab improves EFS in TNBC | Duplicate dataset |
| (Rauch, et al. 2021) [58] | Patient-reported outcomes from thePhase III IMpassion031 trial of neoadjuvant atezolizumab+ chemotherapy in early triple-negative breast cancer | Duplicate |
| (Ramalingam, et al. 2021a) [59] | P83.02 Niraparib + Pembrolizumab (Pembro) Versus Placebo + Pembro 1L Maintenance Therapy in Advanced NSCLC: ZEAL-1L Phase III Study | Non TNBC |
| (Geyer, et al. 2019a) [60] | NSABP B-59/GBG 96-GeparDouze: a randomized double-blind phase III clinical trial of neoadjuvant chemotherapy (NAC) with atezolizumab or placebo in patients (pts) with triple-negative breast cancer (TNBC) followed by adjuvant atezolizumab or placebo | Duplicate |
| (Geyer, et al. 2018) [61] | NSABP B-59/GBG 96-GeparDouze: a randomized double-blind phase III clinical trial of neoadjuvant chemotherapy (NAC) with atezolizumab or placebo in Patients (pts) with triple negative breast cancer (TNBC) followed by adjuvant atezolizumab or placebo | No results yet |
| (Khoury, et al. 2019) [62] | Nivolumab or capecitabine or combination therapy as adjuvant therapy for triple negative breast cancer (TNBC) with residual disease following neoadjuvant chemotherapy: the OXEL study | No results yet |
| NCT02499367 [63] | Nivolumab After Induction Treatment in Triple-negative Breast Cancer (TNBC) Patients | No results yet |
| (Sidaway 2020) [64] | Neoadjuvant therapy improves pCR rate | Duplicate |
| American Association for Cancer Research 2019a [65] | Neoadjuvant Pembrolizumab Takes on TNBC | Duplicate |
| (Ray, et al. 2022) [66] | LBA2 Assessment of Ki67 and FOXC1-based response predictor tracking proliferation and plasticity as a complementary diagnostic for neoadjuvant olaparib+paclitaxel+durvalumab in primary triple-negative breast cancer: retrospective analysis of the I-SPY2 trial | Retrospective analysis |
| (Schmid, et al. 2022a) [67] | KEYNOTE-B49: a phase 3, randomized, double-blind, placebo-controlled study of pembrolizumab plus chemotherapy in patients with HR+/HER2-locally recurrent inoperable or metastatic breast cancer | Non TNBC |
| (Schmid, et al. 2019b) [68] | KEYNOTE-522: phase III study of pembrolizumab (pembro) 1 chemotherapy (chemo) vs placebo (pbo) 1 chemo as neoadjuvant treatment, followed by pembro vs pbo as adjuvant treatment for early triple-negative breast cancer (TNBC) | Duplicate dataset |
| (Schmid, et al. 2017) [69] | KEYNOTE-522: phase III study of pembrolizumab (pembro) 1 chemotherapy (chemo) vs placebo 1 chemo as neoadjuvant followed by pembro vs placebo as adjuvant therapy for triple-negative breast cancer (TNBC) | Duplicate dataset |
| (McArthur, et al. 2022) [70] | KEYNOTE-522: neoadjuvant Pembrolizumab + Chemotherapy vs Placebo + Chemotherapy Followed by Adjuvant Pembrolizumab vs Placebo for Early-stage Triple Negative Breast Cancer | Duplicate dataset |
| (Schmid 2020) [71] | KEYNOTE-522 study of pembro + chemo vs. placebo + chemo as neoadjuvant treatment followed by pembro vs. placebo as adjuvant treatment for early TNBC: pathologic complete response in key subgroups | Duplicate dataset |
| (Cortes Castan, et al. 2017) [72] | KEYNOTE-355: randomized, double-blind, phase III study of pembrolizumab (pembro) + chemotherapy (chemo) vs placebo (pbo) + chemo for previously untreated, locally recurrent, inoperable or metastatic triple-negative breast cancer (mTNBC) | Duplicate dataset |
| (Takano, et al. 2022b) [73] | KEYNOTE-355 Asian subset: Pembrolizumab plus chemotherapy vs placebo plus chemotherapy for triple-negative breast cancer | Duplicate dataset |
| (Cortes, et al. 2019) [74] | KEYNOTE-119: phase III study of pembrolizumab (pembro) versus single-agent chemotherapy (chemo) for metastatic triple negative breast cancer (mTNBC) | Duplicate |
| (Winer, et al. 2016) [75] | KEYNOTE-119: a randomized phase III study of single-agent pembrolizumab (MK-3475) vs single-agent chemotherapy per physician's choice for metastatic triple-negative breast cancer (mTNBC) | Duplicate |
| (Dent, et al. 2018) [76] | IMpassion132: a double-blind randomized phase 3 trial evaluating chemotherapy (CT) ± atezolizumab (atezo) for early progressing locally advanced/metastatic triple-negative breast cancer (mTNBC) | No results yet |
| (Cortés, et al. 2019) [77] | IMpassion132 Phase III trial: Atezolizumab and chemotherapy in early relapsing metastatic triple-negative breast cancer | No results yet |
| (Miles, et al. 2018) [78] | IMpassion131: a phase III study comparing 1L atezolizumab with paclitaxel vs placebo with paclitaxel in treatment-naive patients with inoperable locally advanced or metastatic triple negative breast cancer (TNBC) | Duplicate |
| (Schmid, et al. 2019a) [79] | IMpassion130: updated overall survival (OS) from a global, randomized, double-blind, placebo-controlled, Phase III study of atezolizumab (atezo) + nabpaclitaxel (nP) in previously untreated locally advanced or metastatic triple-negative breast cancer (mTNBC) | Duplicate dataset |
| (Schmid, et al. 2018b) [80] | IMpassion130: results from a global, randomised, double-blind, phase III study of atezolizumab (atezo) + nab-paclitaxel (nab-P) vs placebo + nab-P in treatment-naive, locally advanced or metastatic triple-negative breast cancer (mTNBC) | Duplicate dataset |
| (Von Moos, et al. 2019) [81] | IMpassion130: efficacy in immune biomarker subgroups of atezolizumab + nab-paclitaxel in patients with triple-negative BC | Duplicate dataset |
| (Emens, et al. 2016a) [82] | IMpassion130: a Phase III randomized trial of atezolizumab with nab-paclitaxel for first-line treatment of patients with metastatic triple-negative breast cancer (mTNBC) | Duplicate dataset |
| (Mittendorf, et al. 2018) [83] | IMpassion031: a phase III study comparing neoadjuvant atezolizumab vs placebo in combination with nab-paclitaxel-based chemotherapy in early triple-negative breast cancer (TNBC) | Duplicate dataset |
| (Schmid, et al. 2020a) [84] | Impact of pembrolizumab versus chemotherapy on health-related quality of life in patients with metastatic triple negative breast cancer | Duplicate |
| (Loibl, et al. 2019a) [85] | GeparDouze/NSABP B-59: a randomized double-blind phase III clinical trial of neoadjuvant chemotherapy with atezolizumab or placebo in patients with triple negative breast cancer (TNBC) followed by adjuvant atezolizumab or placebo | Duplicate |
| (Emens, et al. 2021) [86] | First-line atezolizumab plus nab-paclitaxel for unresectable, locally advanced, or metastatic triple-negative breast cancer: IMpassion130 final overall survival analysis | Duplicate dataset |
| (Cortes, et al. 2022a) [87] | Final results of KEYNOTE-355: randomized, double-blind, phase 3 study of pembrolizumab + chemotherapy vs placebo + chemotherapy for previously untreated locally recurrent inoperable or metastatic triple-negative breast cancer | Duplicate dataset |
| (Shah, et al. 2022) [88] | FDA Approval Summary: pembrolizumab for Neoadjuvant and Adjuvant Treatment of Patients with High-Risk Early-Stage Triple Negative Breast Cancer | Review |
| (Savas and Loi 2020) [89] | Expanding the Role for Immunotherapy in Triple-Negative Breast Cancer | Review |
| (Schmid, et al. 2022b) [90] | Event-free Survival with Pembrolizumab in Early Triple-Negative Breast Cancer | Duplicate |
| (Pusztai, et al. 2020) [91] | Evaluation of durvalumab in combination with olaparib and paclitaxel in high-risk HER2 negativestage II/III breast cancer: results from the I-SPY 2 TRIAL | Duplicate |
| American Association for Cancer Research 2019b [92] | Early Pembrolizumab Ups TNBC Responses | Duplicate |
| (Loibl, et al. 2021) [93] | Durvalumab improves long-term outcome in TNBC: results from the phase II randomized GeparNUEVO study investigating neodjuvant durvalumab in addition to an anthracycline/taxane based neoadjuvant chemotherapy in early triple-negative breast cancer (TNBC) | Duplicate |
| (Dalenc, et al. 2020) [94] | Durvalumab compared to maintenance chemotherapy in patients with metastatic breast cancer: results from phase II randomized trial SAFIR02-IMMUNO | Duplicate |
| (Sammons, et al. 2019) [95] | Dora: a randomized phase II multicenter maintenance study of olaparib alone or olaparib in combination with durvalumab in platinum responsive advanced triple-negative breast cancer (aTNBC) | Duplicate |
| EUCTR2020-001651-40 [96] | Comparison of neoadjuvant chemotherapy with PDL1-inhibition (Atezolizumab) and Atezolizumab two-week window to chemotherapy with PDL1-inhibition (Atezolizumab) and identifying biomarkers predicting (early) response to or resistance against Atezolizumab (alone and with CTX) allowing patients stratification in future clinical trials | No results yet |
| (Miles, et al. 2017) [97] | COLET: a multistage, phase 2 study evaluating the safety and efficacy of a doublet regimen of cobimetinib (C) in combination with paclitaxel (P) or triplet regimens of C in combination with atezolizumab (atezo) plus either P or nabpaclitaxel (nab-P) in metastatic triple-negative breast cancer (TNBC) | Duplicate |
| NCT03281954 [98] | Clinical Trial of Neoadjuvant Chemotherapy With Atezolizumab or Placebo in Patients With Triple-Negative Breast Cancer Followed After Surgery by Atezolizumab or Placebo | No results yet |
| NCT03206203 [99] | Carboplatin With or Without Atezolizumab in Treating Patients With Stage IV Triple Negative Breast Cancer | No results yet |
| NCT03971409 [100] | Avelumab With Binimetinib, Utomilumab, or Anti-OX40 Antibody PF-04518600 in Treating Triple Negative Breast Cancer | No results yet |
| (Salgado, et al. 2022) [101] | ATRACTIB: A phase II trial of first-line (1L) atezolizumab (A) in combination with paclitaxel (P) and bevacizumab (B) in metastatic triple-negative breast cancer (mTNBC) | Single arm |
| (Schmid, et al. 2020b) [102] | Atezolizumab plus nab-paclitaxel as first-line treatment for unresectable, locally advanced or metastatic triple-negative breast cancer (IMpassion130): updated efficacy results from a randomised, double-blind, placebo-controlled, phase 3 trial | Duplicate dataset |
| (Schmid, et al. 2018a) [103] | Atezolizumab and Nab-Paclitaxel in Advanced Triple-Negative Breast Cancer | Duplicate dataset |
| (Winer, et al. 2020) [104] | Association of tumor mutational burden (TMB) and clinical outcomes with pembrolizumab (pembro) versus chemotherapy (chemo) in patients with metastatic triple-negative breast cancer (mTNBC) from KEYNOTE-119 | Duplicate dataset |
| (Kyte, et al. 2020) [105] | ALICE: A randomized placebo-controlled phase II study evaluating atezolizumab combined with immunogenic chemotherapy in patients with metastatic triple-negative breast cancer | No results yet |
| (Ignatiadis, et al. 2019) [106] | ALEXANDRA/IMpassion030: a phase III study of standard adjuvant chemotherapy with or without atezolizumab in early stage triple negative breast cancer | No results yet |
| (Rugo, et al. 2021) [107] | Additional efficacy endpoints from thephase 3 KEYNOTE-355 study of pembrolizumab pluschemotherapy vs placebo plus chemotherapy as first-linetherapy for locally recurrent inoperable or metastatic triple-negative breast cancer | Duplicate |
| (Kok, et al. 2017) [108] | Adaptive phase II randomized non-comparative trial of nivolumab after induction treatment in triple negative breast cancer: tONIC-trial | Duplicate |
| (Ramalingam, et al. 2021b) [109] | 1360TiP First-line (1L) maintenance therapy with niraparib (nira) + pembrolizumab (pembro) vs placebo + pembro in advanced/metastatic non-small cell lung cancer (NSCLC): phase III ZEAL-1L study | Non TNBC |
| (Iwata, et al. 2020) [110] | 49MO IMpassion130: final OS analysis from the pivotal phase III study of atezolizumab + nab-paclitaxel vs placebo + nab-paclitaxel in previously untreated locally advanced or metastatic triple-negative breast cancer | Duplicate |
| (Im, et al. 2020) [111] | 44O Pembrolizumab (pembro) vs chemotherapy (chemo) for previously treated metastatic triple-negative breast cancer (mTNBC): KEYNOTE-119 Asia-Pacific subpopulation | Duplicate dataset |
| (Yusof, et al. 2020a) [112] | 43O Phase III KEYNOTE-355 study of pembrolizumab (pembro) vs placebo (pbo) + chemotherapy (chemo) for previously untreated locally recurrent inoperable or metastatic triple-negative breast cancer (TNBC): results for patients (Pts) enrolled in Asia | Duplicate dataset |
| (Saji, et al. 2020) [113] | 3MO IMpassion031: results from a phase III study of neoadjuvant (neoadj) atezolizumab + chemo in early triple-negative breast cancer (TNBC) | Duplicate dataset |
| (Dent, et al. 2020) [114] | 1O KEYNOTE-522 Asian subgroup: phase III study of neoadjuvant pembrolizumab (pembro) vs placebo (pbo) + chemotherapy (chemo) followed by adjuvant pembro vs pbo for early triple-negative breast cancer (TNBC) | Duplicate dataset |
| (Brufsky, et al. 2021) [115] | A phase II randomized trial of cobimetinib plus chemotherapy, with or without atezolizumab, as first-line treatment for patients with locally advanced or metastatic triple-negative breast cancer (COLET): primary analysis | Wrong outcome |
| (Hurvitz, et al. 2022) [116] | Phase Ib/II open-label, randomized trial of atezolizumab (atezo) with ipatasertib (ipat) and fulvestrant (fulv) vs control in MORPHEUS-HR+ breast cancer (M-HR+ BC) and atezo with ipat vs control in MORPHEUS triple negative breast cancer (M-TNBC) | Wrong outcome |
| (Kok, et al. 2018) [117] | Adaptive phase II randomized trial of nivolumab after induction treatment in triple negative breast cancer (TONIC trial): final response data stage I and first translational data | No results yet |
| (Yam, et al. 2021) [118] | Neoadjuvant atezolizumab (atezo) and nab-paclitaxel (nab-p) in patients (pts) with triple-negative breast cancer (TNBC) with suboptimal clinical response to doxorubicin and cyclophosphamide (AC) | Single arm |

**Table S3:** Overall survival treatment ranking and surface under the cumulative ranking curve

|  | Treatments | | | | | | |
| --- | --- | --- | --- | --- | --- | --- | --- |
| Rank | Chemo | Pem+Chemo | Niv+Chemo | Dur | Dur+Chemo | Ate+Chemo | Pem |
| Best | 0 | 0 | 0.2 | 0.4 | 99.4 | 0 | 0 |
| 2nd | 0 | 1.3 | 31.8 | 66 | 0.6 | 0 | 0.3 |
| 3rd | 0 | 16.3 | 48.4 | 29.1 | 0 | 2.7 | 3.5 |
| 4th | 0.3 | 50.3 | 11.1 | 3.6 | 0 | 21 | 13.7 |
| 5th | 14.3 | 23.3 | 4.6 | 0.5 | 0 | 35.9 | 21.4 |
| 6th | 46.3 | 6.6 | 1.7 | 0.2 | 0 | 22.4 | 22.8 |
| Worst | 39.1 | 2.2 | 2.2 | 0.2 | 0 | 18 | 38.3 |
| SUCRA | 12.6 | 46 | 66.3 | 76.8 | 99.9 | 28 | 20.4 |

**Figure S1:** Overall survival using generalized pairwise modelling

 Note: Consistency H index = 1

**Table S4:** Progression free survival treatment ranking and surface under the cumulative ranking curve

|  | Treatment | | | | | |
| --- | --- | --- | --- | --- | --- | --- |
| Rank | Chemo | Ate+Chemo | Dur | Pem+Chemo | Nivolumab+Chemo | Pem |
| Best | 0 | 26.5 | 28.1 | 21.3 | 24.1 | 0 |
| 2nd | 0 | 39.8 | 12.3 | 40.7 | 7.2 | 0 |
| 3rd | 12.3 | 26.9 | 21.1 | 28.8 | 10.8 | 0.1 |
| 4th | 50.3 | 6.8 | 20.5 | 9.1 | 13.1 | 0.2 |
| 5th | 37.3 | 0 | 16.9 | 0.1 | 33.2 | 12.5 |
| Worst | 0.1 | 0 | 1.1 | 0 | 11.6 | 87.2 |
| SUCRA | 35.1 | 76.5 | 62 | 76.5 | 47.5 | 2.4 |

**Figure S2:** Progression free survival using generalized pairwise modelling

Note: Consistency H index = 1

**Table S5:** Pathologic complete response treatment ranking and surface under the cumulative ranking curve

|  | Treatment | | | |
| --- | --- | --- | --- | --- |
| Rank | Chemo | Ate+Chemo | Dur+Chemo | Pem+Chemo |
| Best | 0.1 | 22.8 | 17.5 | 59.6 |
| 2nd | 2.1 | 45.9 | 22.9 | 29.1 |
| 3rd | 30.7 | 26.6 | 33.3 | 9.4 |
| Worst | 67.1 | 4.7 | 26.3 | 1.9 |
| SUCRA | 11.7 | 62.3 | 43.9 | 82.1 |

**Figure S3:** Pathologic complete response using generalized pairwise modelling

Note: Consistency H index = 1.264

**Table S6:** Adverse events grade ≥ 3 treatment ranking and surface under the cumulative Table

|  | Treatment | | | |
| --- | --- | --- | --- | --- |
| Rank | Chemo | Pem+Chemo | Ate+Chemo | Pem |
| Best | 0.7 | 0 | 0 | 99.3 |
| 2nd | 93.3 | 0.8 | 5.4 | 0.5 |
| 3rd | 6 | 27 | 66.8 | 0.2 |
| Worst | 0 | 72.2 | 27.8 | 0 |
| SUCRA | 64.3 | 9.3 | 26.7 | 99.7 |

**Figure S4:** Adrenal insufficiency odds network meta-analysis results. **A** Schematic diagram showing the network map for the treatments included in the analysis. **B** Rankogram showing the ranking probabilities for the least odds of causing this adverse event for each treatment. **C** Forest plot showing each trial effect size and confidence interval as well as the pooled effect size. **D** Bias-adjusted funnel plot showing each treatment separately.


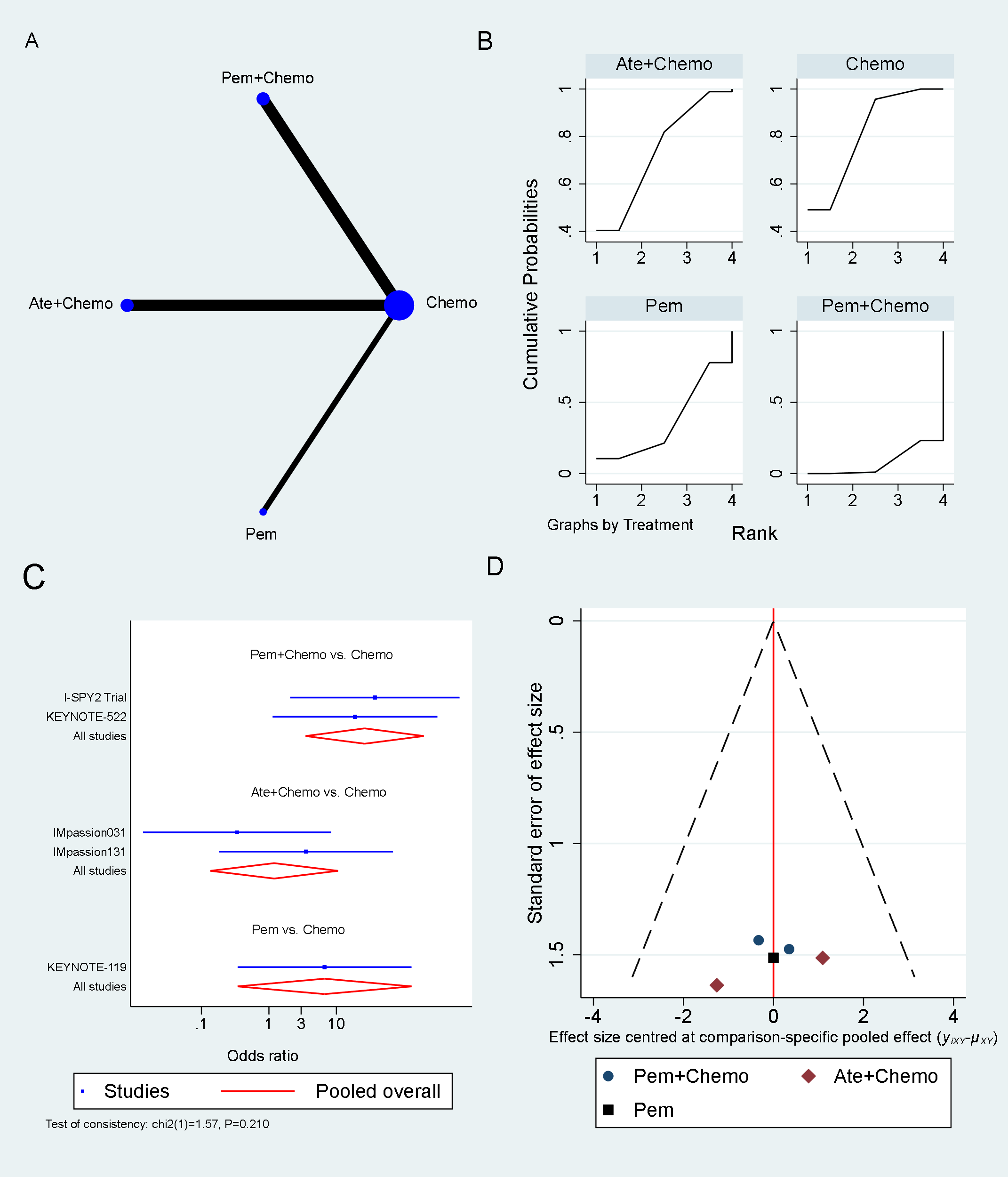


**Figure S5:** Diarrhea odds network meta-analysis results. **A** Schematic diagram showing the network map for the treatments included in the analysis. **B** Rankogram showing the ranking probabilities for the least odds of causing this adverse event for each treatment. **C** Forest plot showing each trial effect size and confidence interval as well as the pooled effect size. **D** Bias-adjusted funnel plot showing each treatment separately.


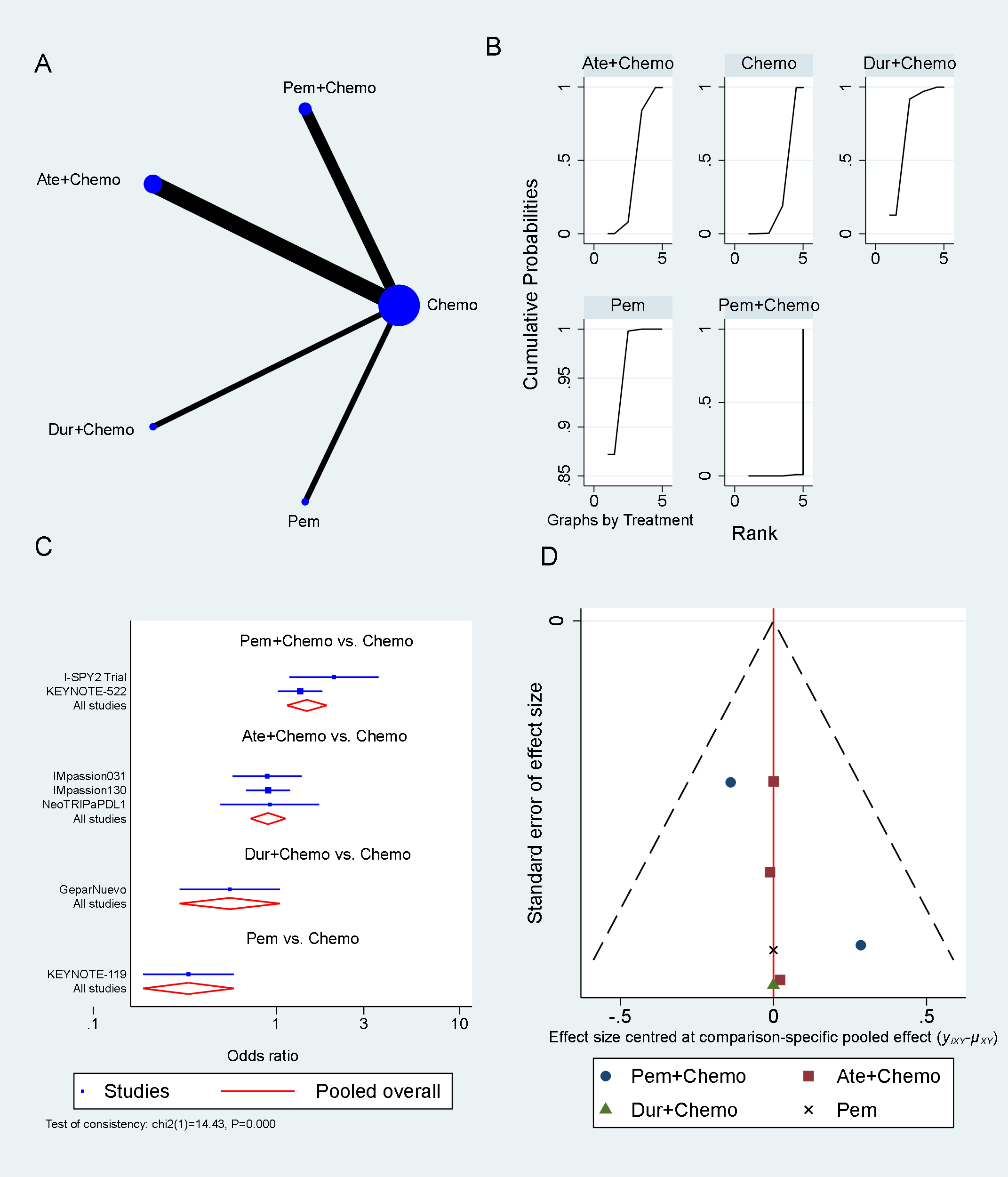


**Figure S6:** Hyperthyroidism odds network meta-analysis results. **A** Schematic diagram showing the network map for the treatments included in the analysis. **B** Rankogram showing the ranking probabilities for the least odds of causing this adverse event for each treatment. **C** Forest plot showing each trial effect size and confidence interval as well as the pooled effect size. **D** Bias-adjusted funnel plot showing each treatment separately.


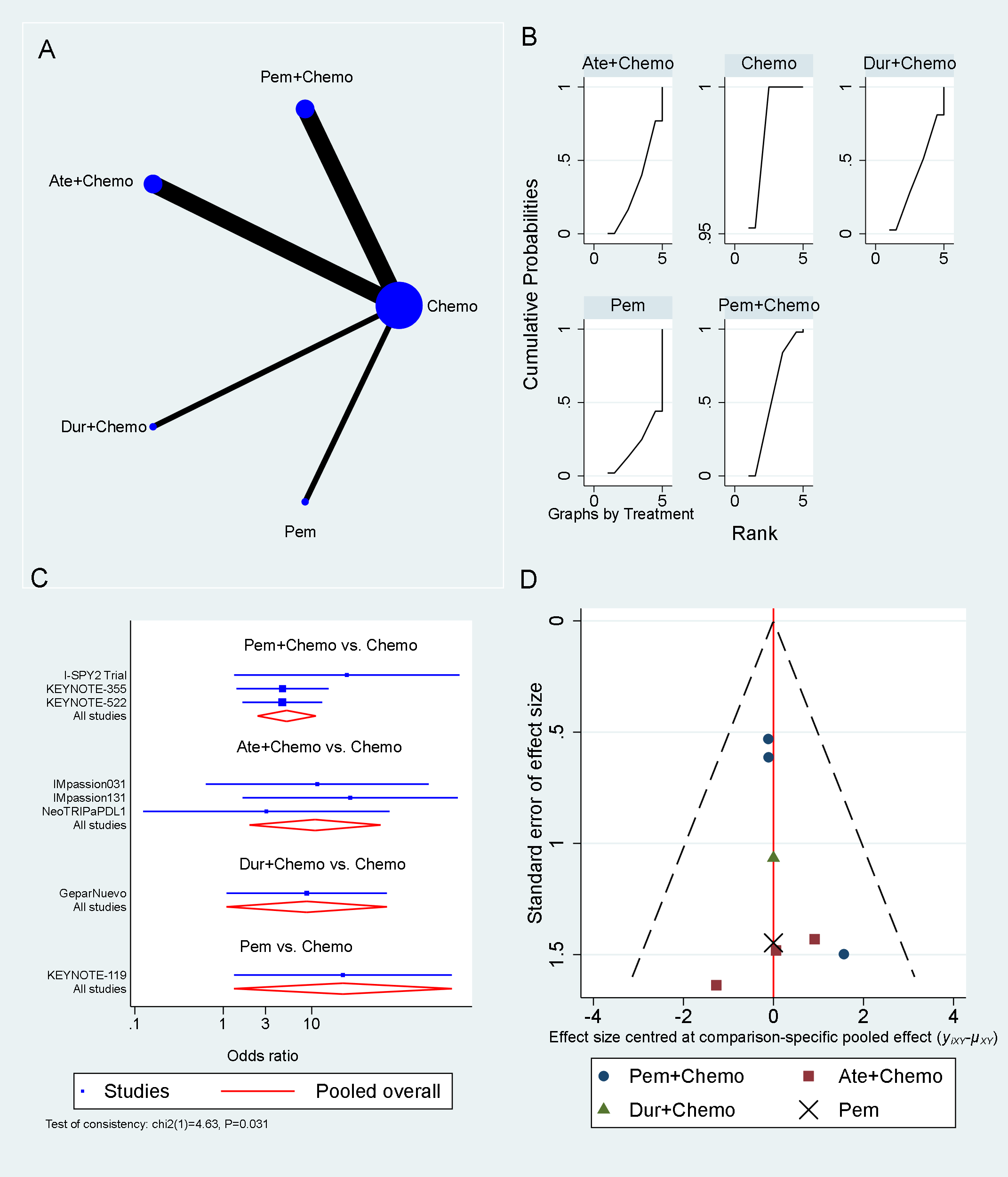


**Figure S7:** Hypothyroidism odds network meta-analysis results. **A** Schematic diagram showing the network map for the treatments included in the analysis. **B** Rankogram showing the ranking probabilities for the least odds of causing this adverse event for each treatment. **C** Forest plot showing each trial effect size and confidence interval as well as the pooled effect size. **D** Bias-adjusted funnel plot showing each treatment separately.


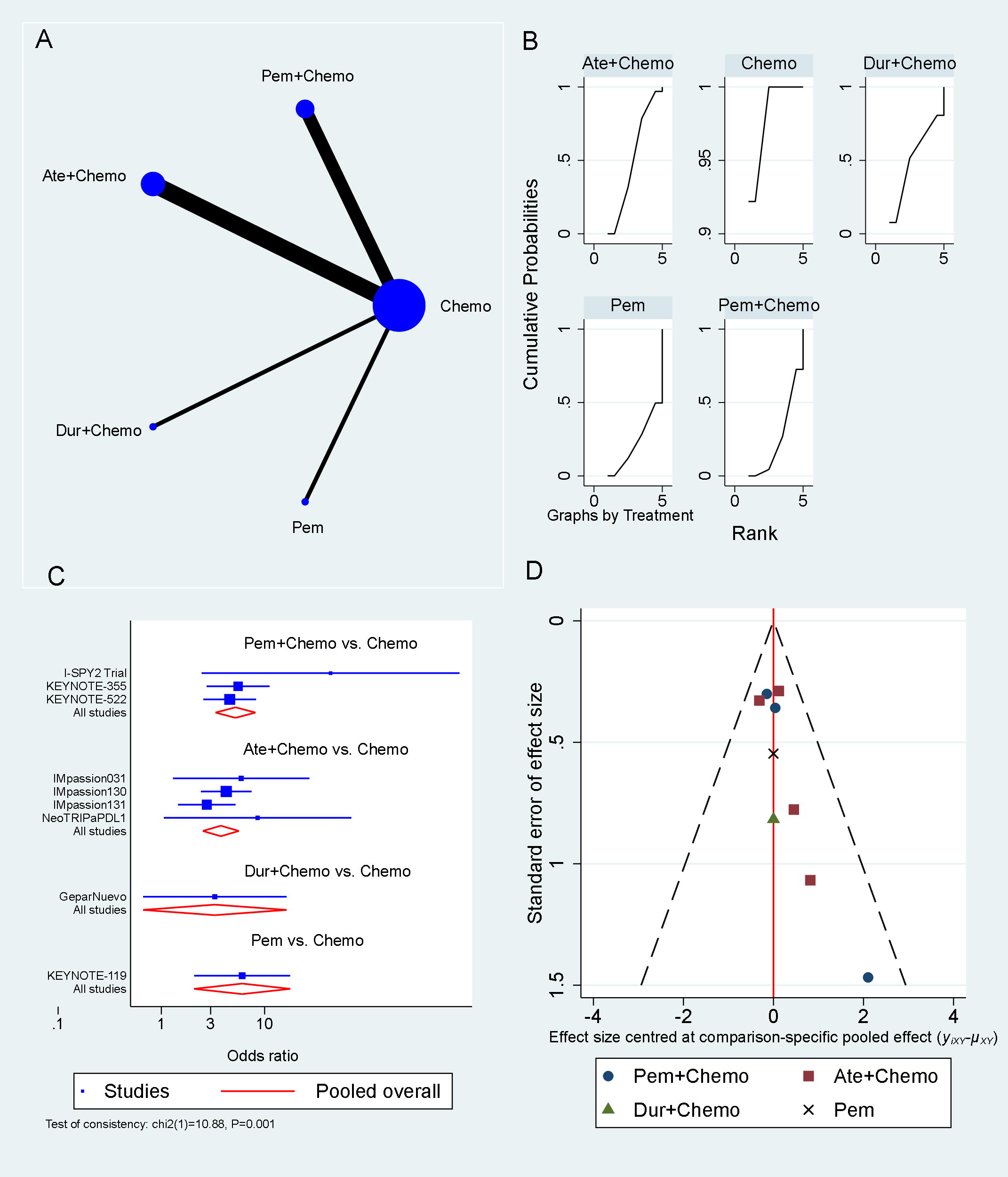


**Figure S8:** Infusion reaction odds network meta-analysis results. **A** Schematic diagram showing the network map for the treatments included in the analysis. **B** Rankogram showing the ranking probabilities for the least odds of causing this adverse event for each treatment. **C** Forest plot showing each trial effect size and confidence interval as well as the pooled effect size. **D** Bias-adjusted funnel plot showing each treatment separately.


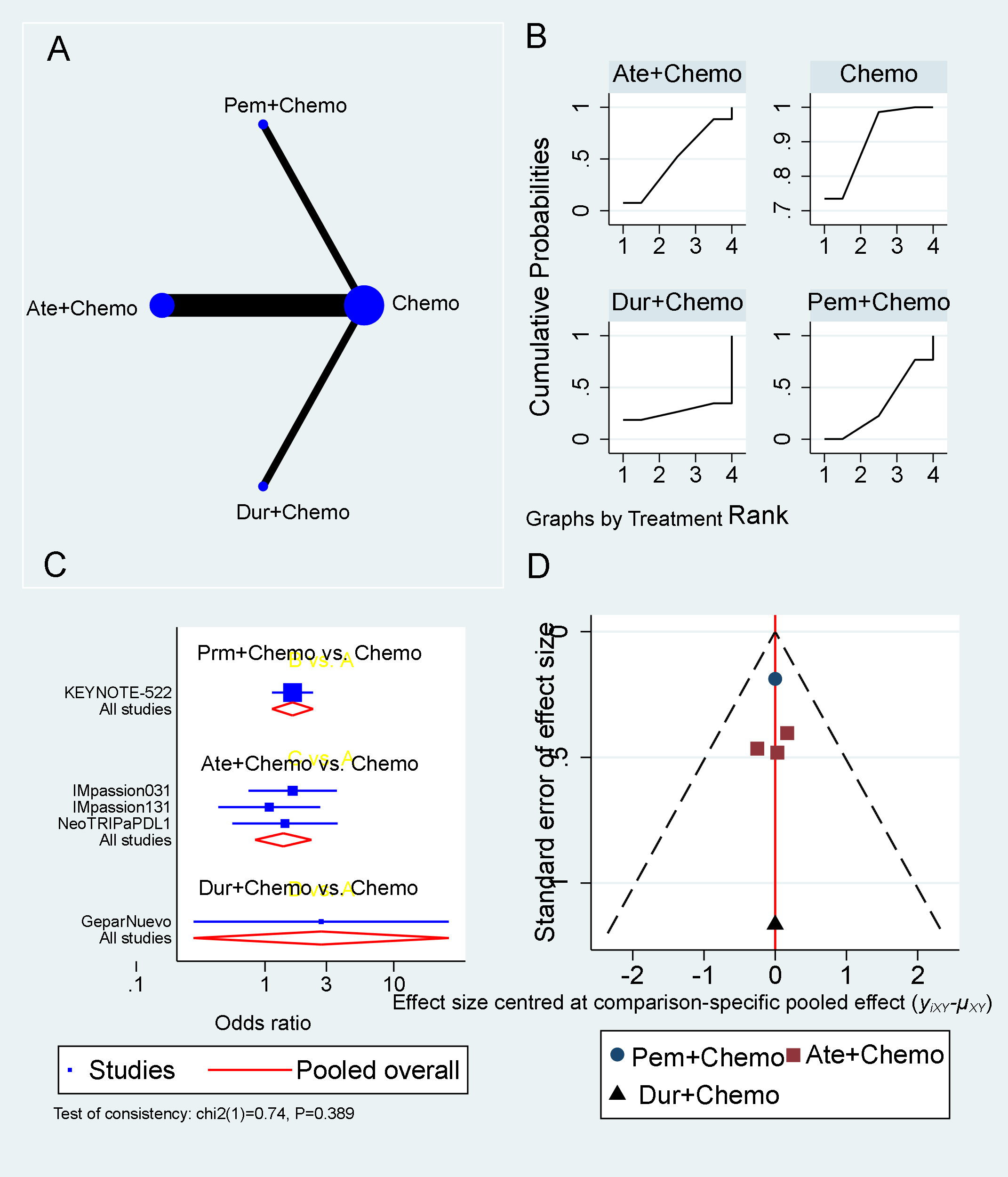


**Figure S9:** Pneumonitis odds network meta-analysis results. **A** Schematic diagram showing the network map for the treatments included in the analysis. **B** Rankogram showing the ranking probabilities for the least odds of causing this adverse event for each treatment. **C** Forest plot showing each trial effect size and confidence interval as well as the pooled effect size. **D** Bias-adjusted funnel plot showing each treatment separately.


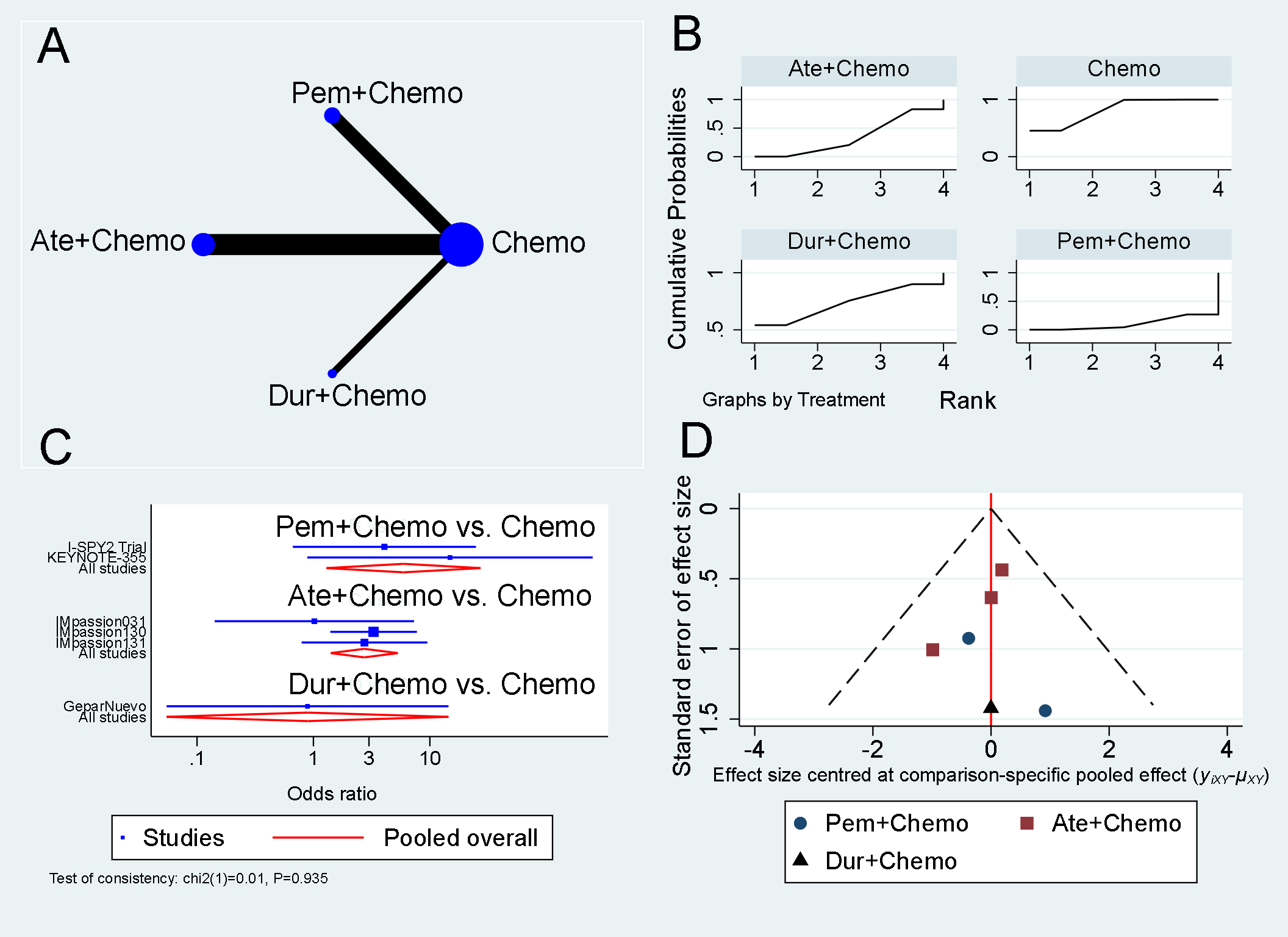


**Figure S10:** Anemia odds network meta-analysis results. **A** Schematic diagram showing the network map for the treatments included in the analysis. **B** Rankogram showing the ranking probabilities for the least odds of causing this adverse event for each treatment. **C** Forest plot showing each trial effect size and confidence interval as well as the pooled effect size. **D** Bias-adjusted funnel plot showing each treatment separately.


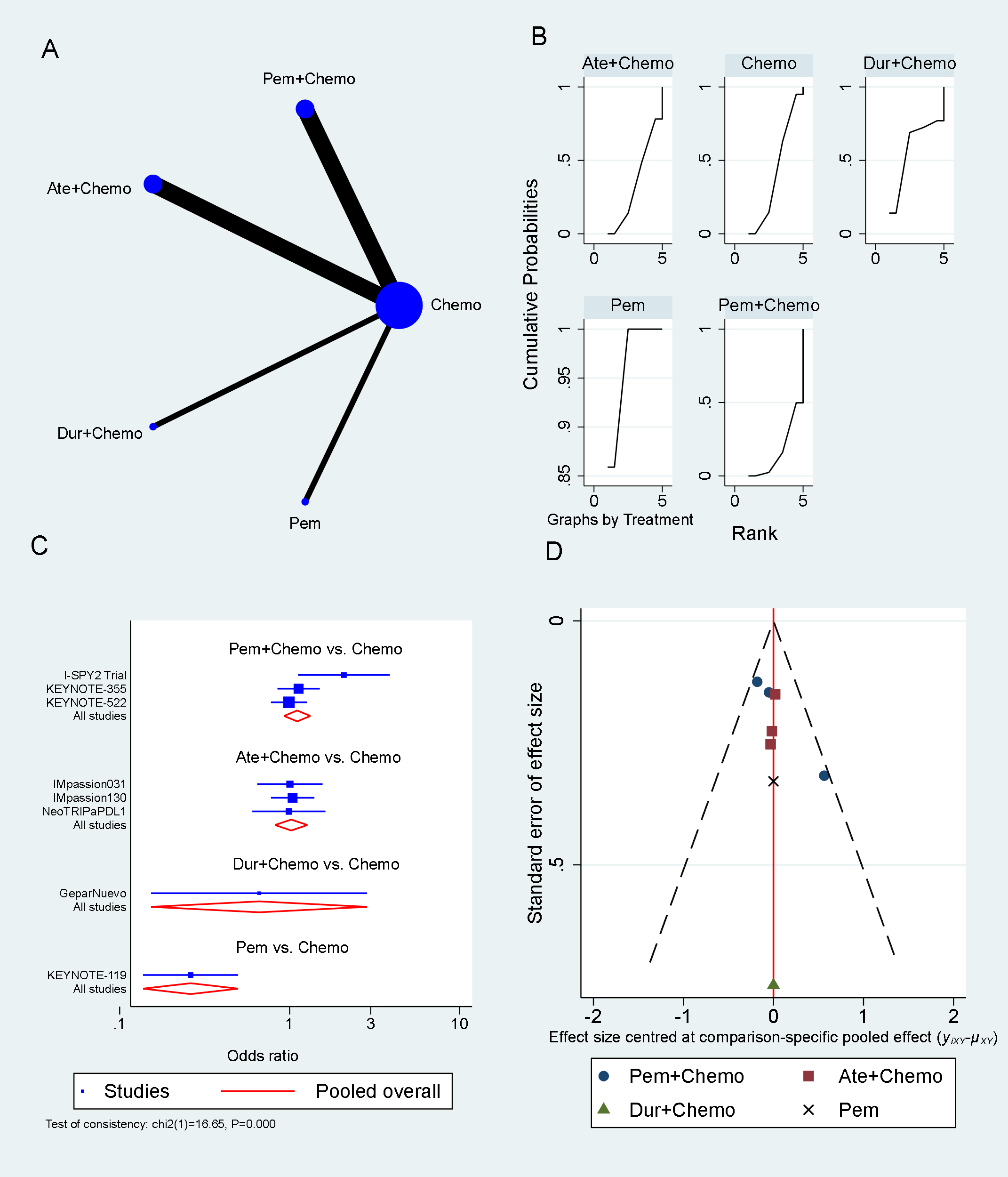


**Figure S11:** Colitis odds network meta-analysis results. **A** Schematic diagram showing the network map for the treatments included in the analysis. **B** Rankogram showing the ranking probabilities for the least odds of causing this adverse event for each treatment. **C** Forest plot showing each trial effect size and confidence interval as well as the pooled effect size. **D** Bias-adjusted funnel plot showing each treatment separately.


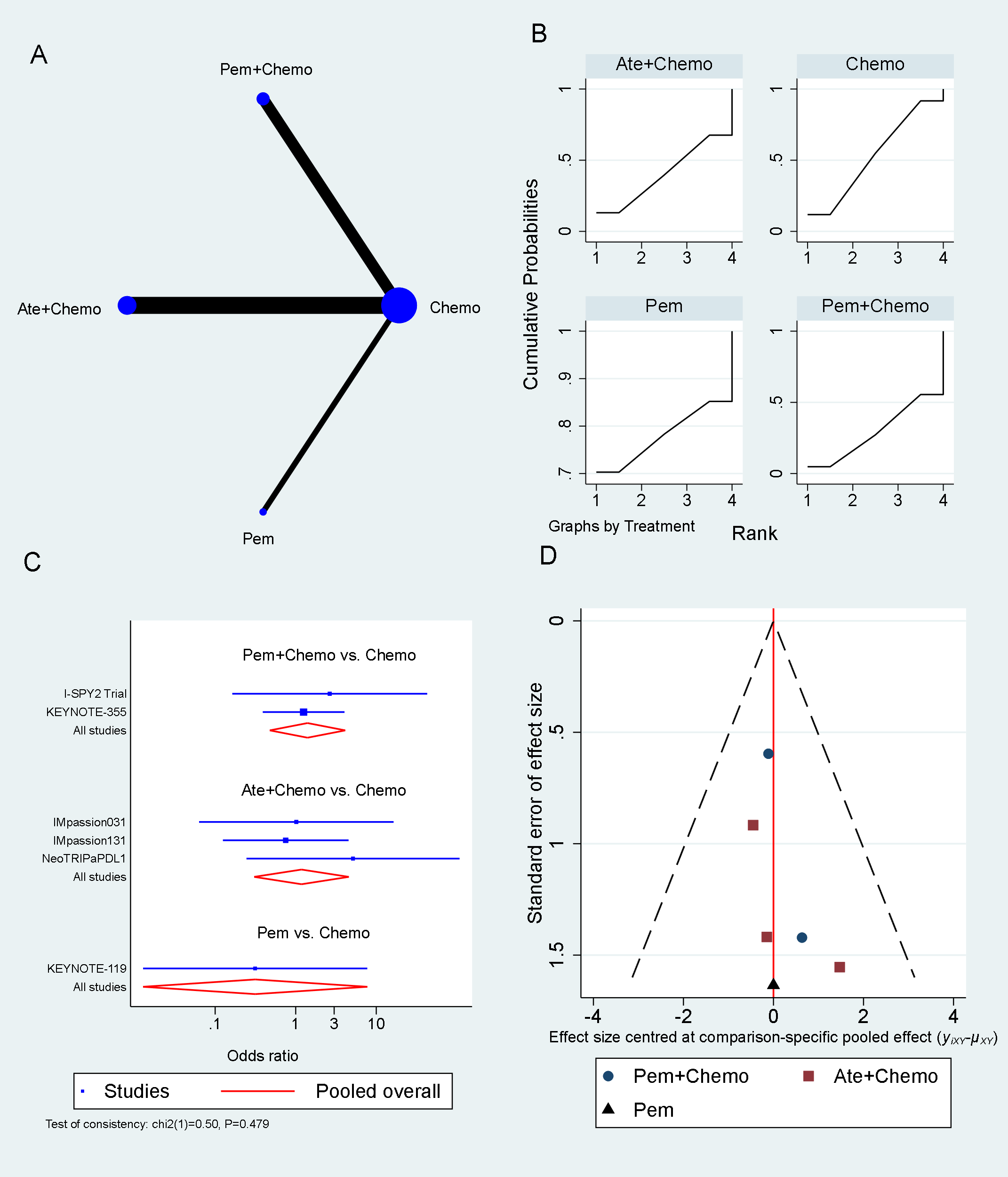


**Figure S12:** Fatigue odds network meta-analysis results. **A** Schematic diagram showing the network map for the treatments included in the analysis. **B** Rankogram showing the ranking probabilities for the least odds of causing this adverse event for each treatment. **C** Forest plot showing each trial effect size and confidence interval as well as the pooled effect size. **D** Bias-adjusted funnel plot showing each treatment separately.


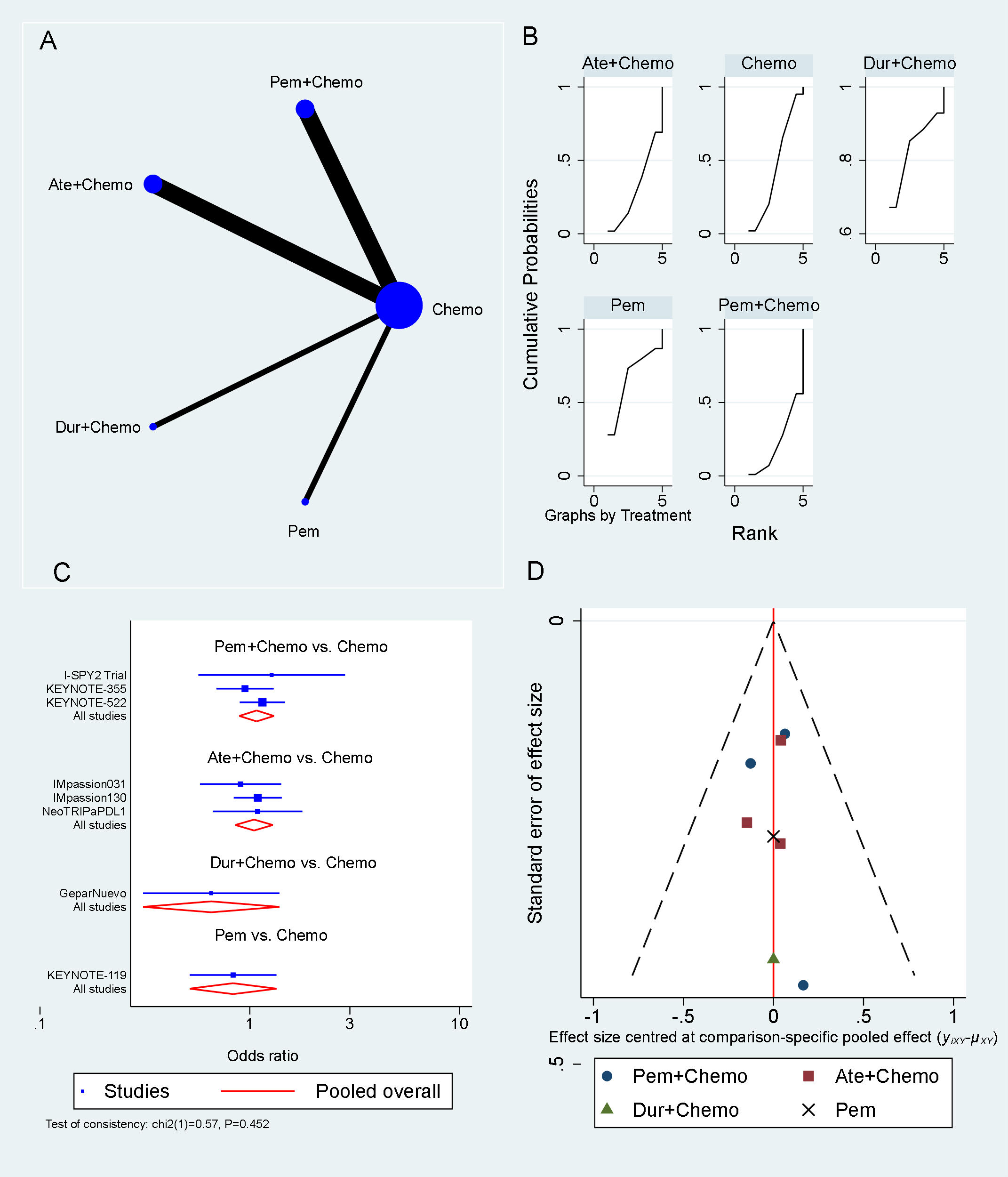


**Figure S13:** Nausea odds network meta-analysis results. **A** Schematic diagram showing the network map for the treatments included in the analysis. **B** Rankogram showing the ranking probabilities for the least odds of causing this adverse event for each treatment. **C** Forest plot showing each trial effect size and confidence interval as well as the pooled effect size. **D** Bias-adjusted funnel plot showing each treatment separately.


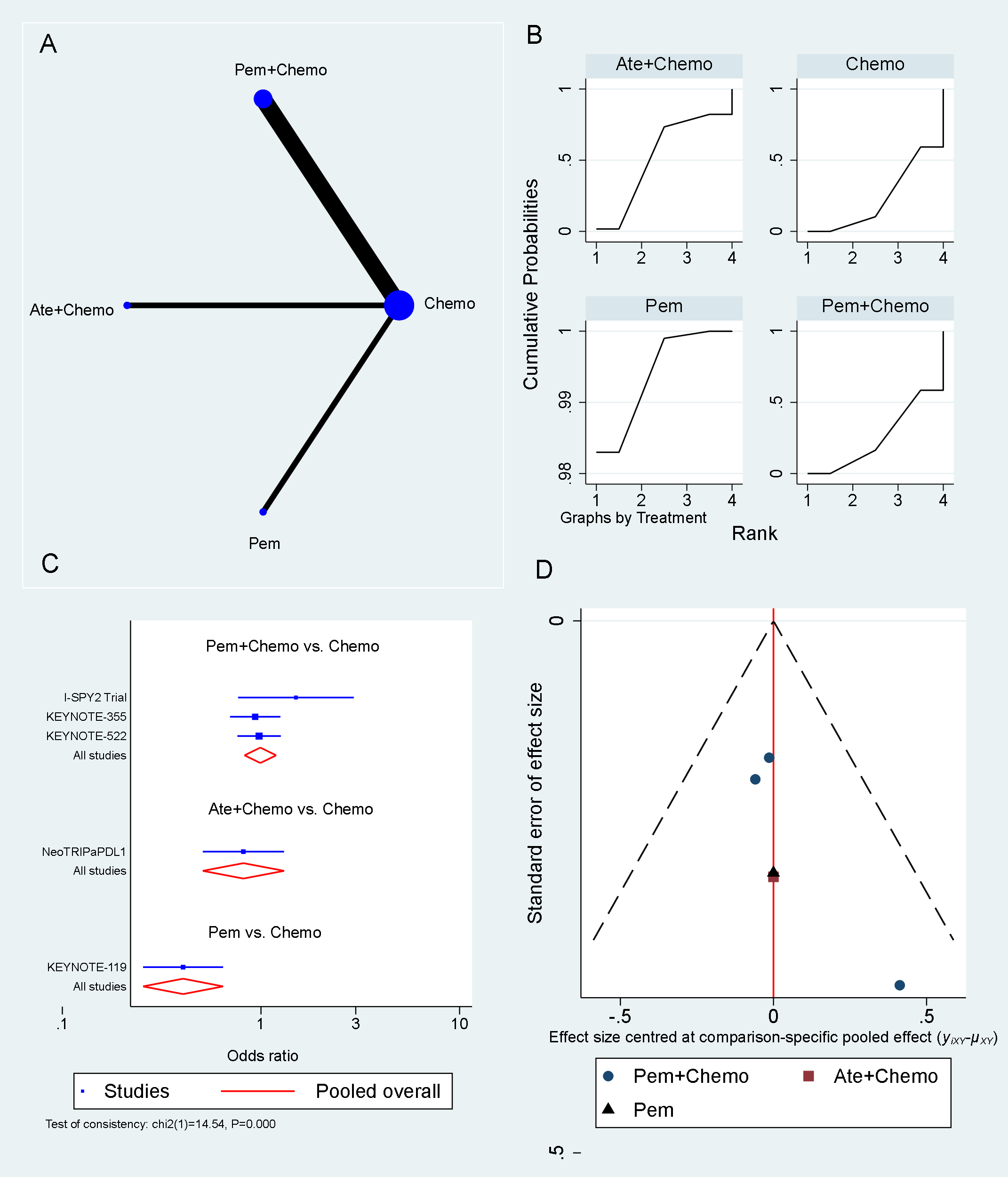


**Figure S14:** Neutropenia odds network meta-analysis results. **A** Schematic diagram showing the network map for the treatments included in the analysis. **B** Rankogram showing the ranking probabilities for the least odds of causing this adverse event for each treatment. **C** Forest plot showing each trial effect size and confidence interval as well as the pooled effect size. **D** Bias-adjusted funnel plot showing each treatment separately.


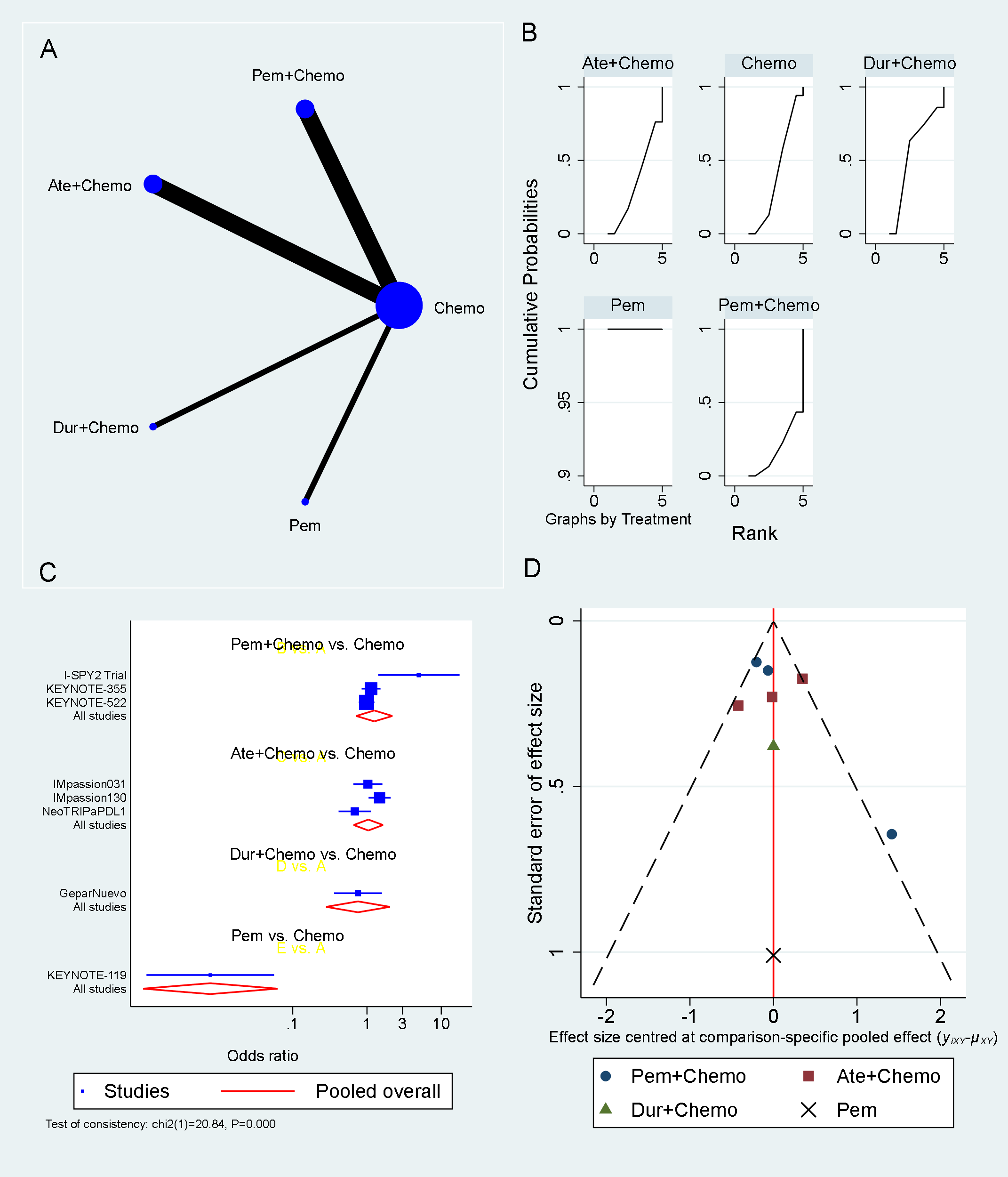


**Figure S15:** Rash odds network meta-analysis results. **A** Schematic diagram showing the network map for the treatments included in the analysis. **B** Rankogram showing the ranking probabilities for the least odds of causing this adverse event for each treatment. **C** Forest plot showing each trial effect size and confidence interval as well as the pooled effect size. **D** Bias-adjusted funnel plot showing each treatment separately.


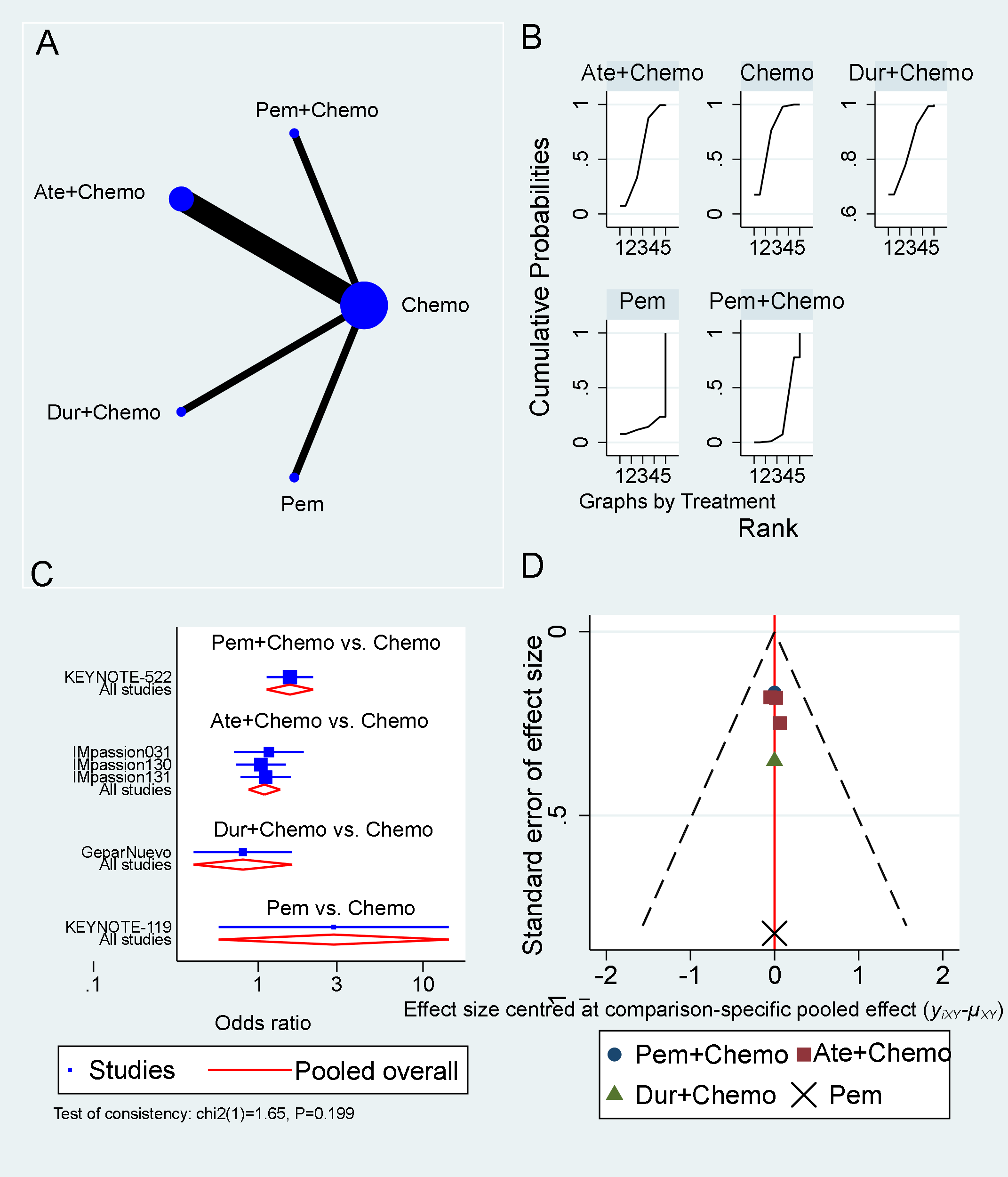


**Figure S16:** Vomiting odds network meta-analysis results. **A** Schematic diagram showing the network map for the treatments included in the analysis. **B** Rankogram showing the ranking probabilities for the least odds of causing this adverse event for each treatment. **C** Forest plot showing each trial effect size and confidence interval as well as the pooled effect size. **D** Bias-adjusted funnel plot showing each treatment separately.


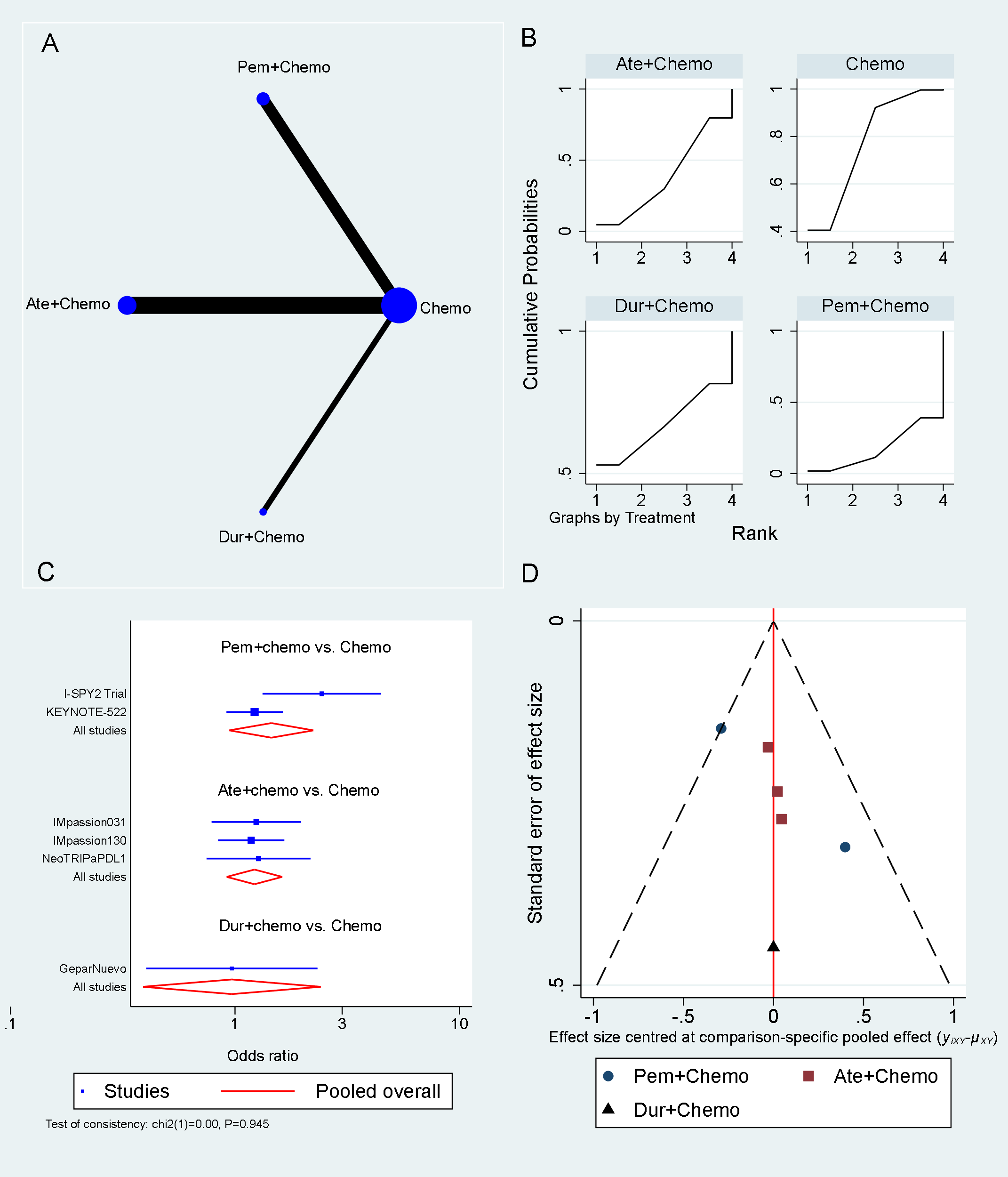


**Figure S17:** Adverse events grade ≥ 3 using generalized pairwise modelling

Note: Consistency H index = 1

**Table S7:** Extracted data used for the analysis

| **Trial** | **Arm A** | **No. Patients Arm A** | **Arm B** | **No. Patients Arm B** | **pCR Arm A** | | **pCR Arm B** | | **OS** | **OS LL** | **OS UP** | **PFS** | **PFS**  **LL** | **PFS UP** | **Adverse events** | **Arm A AEs** | | **Arm B AEs** | |
| --- | --- | --- | --- | --- | --- | --- | --- | --- | --- | --- | --- | --- | --- | --- | --- | --- | --- | --- | --- |
|  |  |  |  |  | **Event** | **Non-event** | **Event** | **Non-event** |  |  |  |  |  |  |  | **Event** | **Non-event** | **Event** | **Non-event** |
| SAFIR02-BREAST | Durvalumab | 47 | Continuation of induction Chemotherapy | 35 |  |  |  |  | 0.54 | 0.3 | 0.97 | 0.87 | 0.54 | 1.42 |  |  |  |  |  |
| KEYNOTE-355 | Pembrolizumab + Investigator’s choice chemotherapy | 566 | Placebo + Investigator’s choice chemotherapy | 281 |  |  |  |  | 0.89 | 0.76 | 1.05 | 0.82 | 0.69 | 0.97 | Any grade | 554 | 8 | 276 | 5 |
|  |  |  |  |  |  |  |  |  |  |  |  |  |  |  | Grade ≥3 | 438 | 124 | 207 | 74 |
|  |  |  |  |  |  |  |  |  |  |  |  |  |  |  | Anemia | 275 | 287 | 129 | 152 |
|  |  |  |  |  |  |  |  |  |  |  |  |  |  |  | Neutropenia | 231 | 331 | 107 | 174 |
|  |  |  |  |  |  |  |  |  |  |  |  |  |  |  | Nausea | 221 | 341 | 115 | 166 |
|  |  |  |  |  |  |  |  |  |  |  |  |  |  |  | Alopecia | 186 | 376 | 94 | 187 |
|  |  |  |  |  |  |  |  |  |  |  |  |  |  |  | Fatigue | 160 | 402 | 83 | 198 |
|  |  |  |  |  |  |  |  |  |  |  |  |  |  |  | ALT increased | 115 | 447 | 46 | 235 |
|  |  |  |  |  |  |  |  |  |  |  |  |  |  |  | Hypothyroidism | 87 | 475 | 9 | 272 |
|  |  |  |  |  |  |  |  |  |  |  |  |  |  |  | Hyperthyroidism | 27 | 535 | 3 | 278 |
|  |  |  |  |  |  |  |  |  |  |  |  |  |  |  | Pneumonitis | 14 | 548 | 0 | 281 |
|  |  |  |  |  |  |  |  |  |  |  |  |  |  |  | Colitis | 10 | 552 | 4 | 277 |
|  |  |  |  |  |  |  |  |  |  |  |  |  |  |  | Severe Skin reactions | 10 | 552 | 1 | 280 |
| Garrido-Castro | Nivolumab + Carboplatin | 32 | Carboplatin | 30 |  |  |  |  | 0.63 | 0.32 | 1.24 | 0.98 | 0.51 | 1.88 |  |  |  |  |  |
| NeoTRIPaPDL1 | Atezolizumab + Carboplatin + Nab-paclitaxel | 138 | Carboplatin + Nab-paclitaxel | 142 | 67 | 71 | 63 | 79 |  |  |  |  |  |  | Grade ≥3 AEs | 107 | 31 | 98 | 42 |
|  |  |  |  |  |  |  |  |  |  |  |  |  |  |  | Any grade AEs | 25 | 113 | 8 | 132 |
|  |  |  |  |  |  |  |  |  |  |  |  |  |  |  | Infusion reactions | 11 | 127 | 8 | 132 |
|  |  |  |  |  |  |  |  |  |  |  |  |  |  |  | Hypothyroidism | 8 | 130 | 1 | 139 |
|  |  |  |  |  |  |  |  |  |  |  |  |  |  |  | Thyroiditis | 2 | 136 | 0 | 140 |
|  |  |  |  |  |  |  |  |  |  |  |  |  |  |  | Hyperthyroidism | 1 | 137 | 0 | 140 |
|  |  |  |  |  |  |  |  |  |  |  |  |  |  |  | Colitis | 2 | 136 | 0 | 140 |
|  |  |  |  |  |  |  |  |  |  |  |  |  |  |  | Pancreatitis | 2 | 136 | 0 | 140 |
|  |  |  |  |  |  |  |  |  |  |  |  |  |  |  | Neutropenia | 86 | 52 | 99 | 41 |
|  |  |  |  |  |  |  |  |  |  |  |  |  |  |  | Nausea | 62 | 76 | 70 | 70 |
|  |  |  |  |  |  |  |  |  |  |  |  |  |  |  | Thrombocytopenia | 57 | 81 | 56 | 84 |
|  |  |  |  |  |  |  |  |  |  |  |  |  |  |  | Liver transaminases | 52 | 86 | 25 | 115 |
|  |  |  |  |  |  |  |  |  |  |  |  |  |  |  | Fatigue | 50 | 88 | 48 | 92 |
|  |  |  |  |  |  |  |  |  |  |  |  |  |  |  | Peripheral neuropathy | 50 | 88 | 48 | 92 |
|  |  |  |  |  |  |  |  |  |  |  |  |  |  |  | Anemia | 47 | 91 | 48 | 92 |
|  |  |  |  |  |  |  |  |  |  |  |  |  |  |  | Vomiting | 40 | 98 | 34 | 106 |
|  |  |  |  |  |  |  |  |  |  |  |  |  |  |  | Leukopenia | 26 | 112 | 34 | 106 |
|  |  |  |  |  |  |  |  |  |  |  |  |  |  |  | Diarrhea | 23 | 115 | 25 | 115 |
|  |  |  |  |  |  |  |  |  |  |  |  |  |  |  | Asthenia | 17 | 121 | 26 | 114 |
| GeparNuevo | Durvalumab + Nab-paclitaxel then epirubicin/cyclophosphamide | 88 | Placebo + Nab-paclitaxel then epirubicin/cyclophosphamide | 86 | 47 | 41 | 38 | 48 | 0.24 | 0.08 | 0.72 |  |  |  | serious AEs | 30 | 62 | 29 | 53 |
|  |  |  |  |  |  |  |  |  |  |  |  |  |  |  | Hypothyroidism | 7 | 85 | 2 | 80 |
|  |  |  |  |  |  |  |  |  |  |  |  |  |  |  | Hyperthyroidism | 9 | 83 | 1 | 81 |
|  |  |  |  |  |  |  |  |  |  |  |  |  |  |  | Anemia | 87 | 5 | 79 | 3 |
|  |  |  |  |  |  |  |  |  |  |  |  |  |  |  | Neutropenia | 71 | 21 | 67 | 15 |
|  |  |  |  |  |  |  |  |  |  |  |  |  |  |  | Vomiting | 12 | 80 | 11 | 71 |
|  |  |  |  |  |  |  |  |  |  |  |  |  |  |  | Infusion reaction | 3 | 89 | 1 | 81 |
|  |  |  |  |  |  |  |  |  |  |  |  |  |  |  | fatigue | 70 | 22 | 68 | 14 |
|  |  |  |  |  |  |  |  |  |  |  |  |  |  |  | hepatitis | 1 | 91 | 0 | 82 |
|  |  |  |  |  |  |  |  |  |  |  |  |  |  |  | stomatitis | 15 | 77 | 14 | 68 |
|  |  |  |  |  |  |  |  |  |  |  |  |  |  |  | skin reaction | 45 | 47 | 39 | 43 |
|  |  |  |  |  |  |  |  |  |  |  |  |  |  |  | sensory neuropathy | 76 | 16 | 69 | 13 |
|  |  |  |  |  |  |  |  |  |  |  |  |  |  |  | rash | 21 | 71 | 22 | 60 |
|  |  |  |  |  |  |  |  |  |  |  |  |  |  |  | pneumonitis | 1 | 91 | 1 | 81 |
|  |  |  |  |  |  |  |  |  |  |  |  |  |  |  | headache | 38 | 54 | 28 | 54 |
|  |  |  |  |  |  |  |  |  |  |  |  |  |  |  | pyrexia | 16 | 76 | 12 | 70 |
|  |  |  |  |  |  |  |  |  |  |  |  |  |  |  | cough | 25 | 67 | 15 | 67 |
|  |  |  |  |  |  |  |  |  |  |  |  |  |  |  | diarrhea | 26 | 66 | 34 | 48 |
| IMpassion131 | Atezolizumab + Paclitaxel | 431 | Placebo + Paclitaxel | 220 |  |  |  |  | 1.12 | 0.88 | 1.43 | 0.86 | 0.7 | 1.05 | Any grade AEs | 268 | 164 | 116 | 217 |
|  |  |  |  |  |  |  |  |  |  |  |  |  |  |  | Grade ≥3 AEs | 49 | 383 | 11 | 206 |
|  |  |  |  |  |  |  |  |  |  |  |  |  |  |  | Hypothyroidism | 60 | 372 | 12 | 205 |
|  |  |  |  |  |  |  |  |  |  |  |  |  |  |  | Hyperthyroidism | 25 | 407 | 0 | 217 |
|  |  |  |  |  |  |  |  |  |  |  |  |  |  |  | hepatitis | 7 | 425 | 2 | 215 |
|  |  |  |  |  |  |  |  |  |  |  |  |  |  |  | Infusion reaction | 15 | 417 | 7 | 210 |
|  |  |  |  |  |  |  |  |  |  |  |  |  |  |  | pneumonitis | 16 | 416 | 3 | 214 |
|  |  |  |  |  |  |  |  |  |  |  |  |  |  |  | Colitis | 3 | 429 | 2 | 215 |
|  |  |  |  |  |  |  |  |  |  |  |  |  |  |  | Rash | 141 | 291 | 66 | 151 |
|  |  |  |  |  |  |  |  |  |  |  |  |  |  |  | Adrenal insufficiency | 3 | 429 | 0 | 217 |
| IMpassion031 | Atezolizumab + Nab-paclitaxel/Doxorubicin/Cyclophosphamide | 165 | Placebo + Nab-paclitaxel/Doxorubicin/Cyclophosphamide | 168 | 95 | 70 | 69 | 99 | 0.69 | 0.25 | 1.87 |  |  |  | Grade ≥3 AEs | 103 | 61 | 101 | 66 |
|  |  |  |  |  |  |  |  |  |  |  |  |  |  |  | serious AEs | 50 | 114 | 30 | 137 |
|  |  |  |  |  |  |  |  |  |  |  |  |  |  |  | Hypothyroidism | 11 | 153 | 2 | 165 |
|  |  |  |  |  |  |  |  |  |  |  |  |  |  |  | Hyperthyroidism | 5 | 159 | 0 | 167 |
|  |  |  |  |  |  |  |  |  |  |  |  |  |  |  | Anemia | 63 | 101 | 64 | 103 |
|  |  |  |  |  |  |  |  |  |  |  |  |  |  |  | Neutropenia | 59 | 105 | 59 | 108 |
|  |  |  |  |  |  |  |  |  |  |  |  |  |  |  | Vomiting | 58 | 106 | 51 | 116 |
|  |  |  |  |  |  |  |  |  |  |  |  |  |  |  | Infusion reaction | 17 | 147 | 11 | 156 |
|  |  |  |  |  |  |  |  |  |  |  |  |  |  |  | Hepatitis | 2 | 162 | 1 | 166 |
|  |  |  |  |  |  |  |  |  |  |  |  |  |  |  | Fatigue | 59 | 105 | 64 | 103 |
|  |  |  |  |  |  |  |  |  |  |  |  |  |  |  | Cough | 35 | 129 | 32 | 135 |
|  |  |  |  |  |  |  |  |  |  |  |  |  |  |  | Rash | 46 | 118 | 42 | 125 |
|  |  |  |  |  |  |  |  |  |  |  |  |  |  |  | Diarrhea | 68 | 96 | 74 | 93 |
|  |  |  |  |  |  |  |  |  |  |  |  |  |  |  | Sensory neuropathy | 54 | 110 | 45 | 122 |
|  |  |  |  |  |  |  |  |  |  |  |  |  |  |  | Stomatitis | 39 | 125 | 29 | 138 |
|  |  |  |  |  |  |  |  |  |  |  |  |  |  |  | pneumonitis | 2 | 162 | 2 | 165 |
|  |  |  |  |  |  |  |  |  |  |  |  |  |  |  | Colitis | 1 | 163 | 1 | 166 |
|  |  |  |  |  |  |  |  |  |  |  |  |  |  |  | Adrenal insufficiency | 0 | 164 | 1 | 166 |
|  |  |  |  |  |  |  |  |  |  |  |  |  |  |  | pyrexia | 34 | 130 | 21 | 146 |
|  |  |  |  |  |  |  |  |  |  |  |  |  |  |  | Elevated transaminase | 33 | 131 | 26 | 141 |
|  |  |  |  |  |  |  |  |  |  |  |  |  |  |  | headache | 41 | 123 | 35 | 132 |
| IMpassion130 | Atezolizumab + Nab-paclitaxel | 451 | Placebo + Nab-paclitaxel | 451 |  |  |  |  | 0.84 | 0.69 | 1.02 | 0.8 | 0.69 | 0.92 | Any grade AEs | 450 | 3 | 428 | 9 |
|  |  |  |  |  |  |  |  |  |  |  |  |  |  |  | Grade ≥3 AEs | 230 | 223 | 190 | 247 |
|  |  |  |  |  |  |  |  |  |  |  |  |  |  |  | Hypothyroidism | 63 | 390 | 16 | 421 |
|  |  |  |  |  |  |  |  |  |  |  |  |  |  |  | serious AEs | 105 | 348 | 81 | 356 |
|  |  |  |  |  |  |  |  |  |  |  |  |  |  |  | Anemia | 126 | 327 | 118 | 319 |
|  |  |  |  |  |  |  |  |  |  |  |  |  |  |  | Neutropenia | 97 | 356 | 68 | 369 |
|  |  |  |  |  |  |  |  |  |  |  |  |  |  |  | Vomiting | 89 | 364 | 75 | 362 |
|  |  |  |  |  |  |  |  |  |  |  |  |  |  |  | fatigue | 212 | 241 | 195 | 242 |
|  |  |  |  |  |  |  |  |  |  |  |  |  |  |  | sensory neuropathy | 72 | 381 | 52 | 385 |
|  |  |  |  |  |  |  |  |  |  |  |  |  |  |  | rash | 78 | 375 | 73 | 364 |
|  |  |  |  |  |  |  |  |  |  |  |  |  |  |  | pneumonitis | 23 | 430 | 7 | 430 |
|  |  |  |  |  |  |  |  |  |  |  |  |  |  |  | headache | 108 | 345 | 95 | 342 |
|  |  |  |  |  |  |  |  |  |  |  |  |  |  |  | pyrexia | 88 | 365 | 48 | 389 |
|  |  |  |  |  |  |  |  |  |  |  |  |  |  |  | cough | 114 | 339 | 85 | 352 |
|  |  |  |  |  |  |  |  |  |  |  |  |  |  |  | diarrhea | 147 | 306 | 152 | 285 |
|  |  |  |  |  |  |  |  |  |  |  |  |  |  |  | Elevated transaminase | 45 | 408 | 43 | 394 |
| Nci 10013 | Carboplatin/Paclitaxel | 16 | Atezolizumab + Carboplatin/Paclitaxel | 45 | 3 | 13 | 25 | 20 |  |  |  |  |  |  | Any grade AEs | 4 | 12 | 10 | 35 |
|  |  |  |  |  |  |  |  |  |  |  |  |  |  |  | Grade ≥3 AEs | 0 | 16 | 2 | 43 |
| KEYNOTE-522 | Pembrolizumab + Paclitaxel/Carboplatin + Doxorubicin OR Epirubicin/Cyclophosphamide | 401 | Placebo + Paclitaxel/Carboplatin + Doxorubicin OR Epirubicin/Cyclophosphamide | 201 | 260 | 141 | 103 | 98 | 0.72 | 0.51 | 1.02 |  |  |  | Grade ≥3 AEs | 633 | 148 | 295 | 94 |
|  |  |  |  |  |  |  |  |  |  |  |  |  |  |  | Any grade AEs | 777 | 4 | 389 | 0 |
|  |  |  |  |  |  |  |  |  |  |  |  |  |  |  | Infusion reactions | 132 | 649 | 43 | 346 |
|  |  |  |  |  |  |  |  |  |  |  |  |  |  |  | Hypothyroidism | 107 | 674 | 13 | 376 |
|  |  |  |  |  |  |  |  |  |  |  |  |  |  |  | Hyperthyroidism | 36 | 745 | 4 | 385 |
|  |  |  |  |  |  |  |  |  |  |  |  |  |  |  | Neutropenia | 365 | 416 | 183 | 206 |
|  |  |  |  |  |  |  |  |  |  |  |  |  |  |  | Nausea | 490 | 291 | 246 | 143 |
|  |  |  |  |  |  |  |  |  |  |  |  |  |  |  | Liver transaminases | 199 | 582 | 96 | 293 |
|  |  |  |  |  |  |  |  |  |  |  |  |  |  |  | Fatigue | 321 | 460 | 147 | 242 |
|  |  |  |  |  |  |  |  |  |  |  |  |  |  |  | Peripheral neuropathy | 154 | 627 | 82 | 307 |
|  |  |  |  |  |  |  |  |  |  |  |  |  |  |  | Anemia | 430 | 351 | 215 | 174 |
|  |  |  |  |  |  |  |  |  |  |  |  |  |  |  | Vomiting | 199 | 582 | 85 | 304 |
|  |  |  |  |  |  |  |  |  |  |  |  |  |  |  | Diarrhea | 230 | 551 | 92 | 297 |
|  |  |  |  |  |  |  |  |  |  |  |  |  |  |  | Alopecia | 471 | 310 | 220 | 169 |
|  |  |  |  |  |  |  |  |  |  |  |  |  |  |  | Asthenia | 191 | 590 | 99 | 290 |
|  |  |  |  |  |  |  |  |  |  |  |  |  |  |  | Constipation | 185 | 596 | 82 | 307 |
|  |  |  |  |  |  |  |  |  |  |  |  |  |  |  | Rash | 170 | 611 | 59 | 330 |
|  |  |  |  |  |  |  |  |  |  |  |  |  |  |  | Severe skin reaction | 34 | 747 | 4 | 385 |
|  |  |  |  |  |  |  |  |  |  |  |  |  |  |  | Adrenal insufficiency | 18 | 763 | 0 | 389 |
| KEYNOTE-119 | Pembrolizumab | 312 | Investigator’s choice mono-chemotherapy | 310 | 49 | 272 | 103 | 207 | 0.97 | 0.82 | 1.15 | 1.6 | 1.33 | 1.92 | Grade ≥3 AEs | 43 | 266 | 105 | 187 |
|  |  |  |  |  |  |  |  |  |  |  |  |  |  |  | Hypothyroidism | 24 | 285 | 4 | 288 |
|  |  |  |  |  |  |  |  |  |  |  |  |  |  |  | Thyroiditis | 1 | 308 | 1 | 291 |
|  |  |  |  |  |  |  |  |  |  |  |  |  |  |  | Hyperthyroidism | 11 | 298 | 0 | 292 |
|  |  |  |  |  |  |  |  |  |  |  |  |  |  |  | Colitis | 0 | 309 | 1 | 291 |
|  |  |  |  |  |  |  |  |  |  |  |  |  |  |  | Neutropenia | 1 | 308 | 86 | 206 |
|  |  |  |  |  |  |  |  |  |  |  |  |  |  |  | Nausea | 31 | 278 | 63 | 229 |
|  |  |  |  |  |  |  |  |  |  |  |  |  |  |  | Thrombocytopenia | 2 | 307 | 7 | 285 |
|  |  |  |  |  |  |  |  |  |  |  |  |  |  |  | Liver transaminases | 33 | 276 | 41 | 251 |
|  |  |  |  |  |  |  |  |  |  |  |  |  |  |  | Fatigue | 37 | 272 | 41 | 251 |
|  |  |  |  |  |  |  |  |  |  |  |  |  |  |  | Peripheral neuropathy | 4 | 305 | 19 | 273 |
|  |  |  |  |  |  |  |  |  |  |  |  |  |  |  | Anemia | 13 | 296 | 42 | 250 |
|  |  |  |  |  |  |  |  |  |  |  |  |  |  |  | Leukopenia | 3 | 306 | 7 | 285 |
|  |  |  |  |  |  |  |  |  |  |  |  |  |  |  | Diarrhea | 18 | 291 | 46 | 246 |
|  |  |  |  |  |  |  |  |  |  |  |  |  |  |  | Alopecia | 2 | 307 | 39 | 253 |
|  |  |  |  |  |  |  |  |  |  |  |  |  |  |  | Asthenia | 19 | 290 | 25 | 267 |
|  |  |  |  |  |  |  |  |  |  |  |  |  |  |  | Constipation | 12 | 297 | 29 | 263 |
|  |  |  |  |  |  |  |  |  |  |  |  |  |  |  | Rash | 6 | 303 | 2 | 290 |
|  |  |  |  |  |  |  |  |  |  |  |  |  |  |  | Severe skin reaction | 6 | 303 | 1 | 291 |
|  |  |  |  |  |  |  |  |  |  |  |  |  |  |  | Adrenal insufficiency | 3 | 306 | 0 | 292 |
| I-SPY2 Trial | Pembrolizumab + Paclitaxel + Doxorubicin/Cyclophosphamide | 28 | Paclitaxel + Doxorubicin/Cyclophosphamide | 79 | 17 | 11 | 17 | 62 |  |  |  |  |  |  | Grade ≥3 AEs | 38 | 31 | 36 | 145 |
|  |  |  |  |  |  |  |  |  |  |  |  |  |  |  | Neutropenia | 7 | 62 | 4 | 177 |
|  |  |  |  |  |  |  |  |  |  |  |  |  |  |  | Anemia | 23 | 46 | 35 | 146 |
|  |  |  |  |  |  |  |  |  |  |  |  |  |  |  | Fatigue | 60 | 9 | 152 | 29 |
|  |  |  |  |  |  |  |  |  |  |  |  |  |  |  | Nausea | 55 | 14 | 131 | 50 |
|  |  |  |  |  |  |  |  |  |  |  |  |  |  |  | Vomiting | 26 | 43 | 36 | 145 |
|  |  |  |  |  |  |  |  |  |  |  |  |  |  |  | diarrhea | 39 | 30 | 70 | 111 |
|  |  |  |  |  |  |  |  |  |  |  |  |  |  |  | Peripheral neuropathy | 48 | 21 | 124 | 57 |
|  |  |  |  |  |  |  |  |  |  |  |  |  |  |  | Hypothyroidism | 7 | 62 | 0 | 181 |
|  |  |  |  |  |  |  |  |  |  |  |  |  |  |  | Hyperthyroidism | 4 | 65 | 0 | 181 |
|  |  |  |  |  |  |  |  |  |  |  |  |  |  |  | Adrenal insufficiency | 6 | 63 | 0 | 181 |
|  |  |  |  |  |  |  |  |  |  |  |  |  |  |  | hepatitis | 2 | 67 | 0 | 181 |
|  |  |  |  |  |  |  |  |  |  |  |  |  |  |  | pneumonitis | 3 | 66 | 2 | 179 |
|  |  |  |  |  |  |  |  |  |  |  |  |  |  |  | Colitis | 1 | 68 | 1 | 180 |
|  |  |  |  |  |  |  |  |  |  |  |  |  |  |  | Pruritus | 22 | 47 | 22 | 159 |

Abbreviations: pCR, pathologic Complete Response; OS, Overall Survival; LL, Lower Limit; UP, Upper Limit; PFS, Progression Free Survival; AE, Adverse Event; ALT, Alanine Transaminase.

**References**

1. Nct: **Veliparib and Atezolizumab Either Alone or in Combination in Treating Patients With Stage III-IV Triple Negative Breast Cancer.** [*https://clinicaltrialsgov/show/NCT02849496*](https://clinicaltrialsgov/show/NCT02849496) 2016.

2. Nct: **Triple-B Study;Carboplatin-cyclophosphamide Versus Paclitaxel With or Without Atezolizumab as First-line Treatment in Advanced Triple Negative Breast Cancer.** [*https://clinicaltrialsgov/show/NCT01898117*](https://clinicaltrialsgov/show/NCT01898117) 2013.

3. Euctr AT: **A trial of MPDL3280A and nab-Paclitaxel in metastatic triple negative breast cancer.** [*https://trialsearchwhoint/Trial2aspx?TrialID=EUCTR2014-005490-37-AT*](https://trialsearchwhoint/Trial2aspx?TrialID=EUCTR2014-005490-37-AT) 2016.

4. Vidula N, Nanda R, Miller K, Pohlmann P, Abramson V, Emens LA, Park BH, Liu MC, Goga A, Rugo HS: **Translational breast cancer researchconsortium 044 trial: randomized phase 2 study of pembrolizumab and carboplatin versus carboplatin alonefor chest wall recurrence of breast cancer.** *Cancer research* 2021, **81**.

5. Saji S, Ohsumi S, Ito M, Hayashi N, Kobayashi K, Masuda N, Niikura N, Yamashita T, Kiyama K, Hasegawa A, et al.: **Subgroup analysis of Japanese patients in a phase III randomized, controlled study of neoadjuvant atezolizumab or placebo, combined with nab-paclitaxel and anthracycline-based chemotherapy in early triple-negative breast cancer (IMpassion031).** *Japanese journal of clinical oncology* 2022**:**1124‐1133.

6. Nct: **A Study to Investigate Atezolizumab and Chemotherapy Compared With Placebo and Chemotherapy in the Neoadjuvant Setting in Participants With Early Stage Triple Negative Breast Cancer.** [*https://clinicaltrialsgov/show/NCT03197935*](https://clinicaltrialsgov/show/NCT03197935) 2017.

7. Rpcec: **A Study of the Efficacy and Safety of Atezolizumab Plus Chemotherapy for Patients With Early Relapsing Recurrent Triple-Negative Breast Cancer.** [*https://trialsearchwhoint/Trial2aspx?TrialID=RPCEC00000280*](https://trialsearchwhoint/Trial2aspx?TrialID=RPCEC00000280) 2018.

8. Nct: **Study of Single Agent Pembrolizumab (MK-3475) Versus Single Agent Chemotherapy for Metastatic Triple Negative Breast Cancer (MK-3475-119/KEYNOTE-119).** [*https://clinicaltrialsgov/show/NCT02555657*](https://clinicaltrialsgov/show/NCT02555657) 2015.

9. Nct: **Study of Pembrolizumab (MK-3475) Plus Chemotherapy vs. Placebo Plus Chemotherapy for Previously Untreated Locally Recurrent Inoperable or Metastatic Triple Negative Breast Cancer (MK-3475-355/KEYNOTE-355).** [*https://clinicaltrialsgov/show/NCT02819518*](https://clinicaltrialsgov/show/NCT02819518) 2016.

10. Nct: **Study of Pembrolizumab (MK-3475) Plus Chemotherapy vs Placebo Plus Chemotherapy as Neoadjuvant Therapy and Pembrolizumab vs Placebo as Adjuvant Therapy in Participants With Triple Negative Breast Cancer (TNBC) (MK-3475-522/KEYNOTE-522).** [*https://clinicaltrialsgov/show/NCT03036488*](https://clinicaltrialsgov/show/NCT03036488) 2017.

11. Nct: **A Study of Cobimetinib Plus Paclitaxel, Cobimetinib Plus Atezolizumab Plus Paclitaxel, or Cobimetinib Plus Atezolizumab Plus Nab-Paclitaxel as Initial Treatment for Participants With Triple-Negative Breast Cancer That Has Spread.** [*https://clinicaltrialsgov/show/NCT02322814*](https://clinicaltrialsgov/show/NCT02322814) 2014.

12. Euctr DE: **A Study of Atezolizumab with Neoadjuvant Anthracycline/Nab-Paclitaxel Chemotherapy Compared with Placebo and Chemotherapy in Patients with Early Triple-Negative Breast Cancer.** [*https://trialsearchwhoint/Trial2aspx?TrialID=EUCTR2016-004734-22-DE*](https://trialsearchwhoint/Trial2aspx?TrialID=EUCTR2016-004734-22-DE) 2017.

13. Nct: **A Study of Atezolizumab in Combination With Nab-Paclitaxel Compared With Placebo With Nab-Paclitaxel for Participants With Previously Untreated Metastatic Triple-Negative Breast Cancer (IMpassion130).** [*https://clinicaltrialsgov/show/NCT02425891*](https://clinicaltrialsgov/show/NCT02425891) 2015.

14. Ctri: **A Study of Atezolizumab and Paclitaxel Versus Placebo and Paclitaxel in Participants With Previously Untreated Locally Advanced or Metastatic Triple Negative Breast Cancer (TNBC) (IMpassion131).** [*https://trialsearchwhoint/Trial2aspx?TrialID=CTRI/2017/10/010010*](https://trialsearchwhoint/Trial2aspx?TrialID=CTRI/2017/10/010010) 2017.

15. Nct: **A Study of Atezolizumab and Paclitaxel Versus Placebo and Paclitaxel in Participants With Previously Untreated Locally Advanced or Metastatic Triple Negative Breast Cancer (TNBC).** [*https://clinicaltrialsgov/show/NCT03125902*](https://clinicaltrialsgov/show/NCT03125902) 2017.

16. Euctr DE: **A Study of Atezolizumab (Anti-PD-L1 Antibody) in Combination with Paclitaxel Compared with Placebo with Paclitaxel for Patients with Previously Untreated Inoperable Locally Advanced or Metastatic Triple Negative Breast Cancer.** [*https://trialsearchwhoint/Trial2aspx?TrialID=EUCTR2016-004024-29-DE*](https://trialsearchwhoint/Trial2aspx?TrialID=EUCTR2016-004024-29-DE) 2017.

17. Nct: **Sacituzumab Govitecan +/- Pembrolizumab in Metastatic TNBC.** [*https://clinicaltrialsgov/show/NCT04468061*](https://clinicaltrialsgov/show/NCT04468061) 2020.

18. Park IH, Kim GM, Kim JH, Kim H, Park KH, Park YH, Baek SK, Sim SH, Ahn HK, Lee GW, et al.: **Randomized, phase II trial to evaluate the efficacy and safety of atezolizumab plus capecitabine adjuvant therapy compared to capecitabine monotherapy for triple receptor-negative breast cancer (TNBC) with residual invasive cancer after neoadjuvant chemotherapy (MIRINAE trial, KCSG-BR18-21).** *Journal of clinical oncology* 2020, **38**.

19. Loibl S, Jackisch C, Seiler S, Rastogi P, Huober J, Denkert C, Schneeweiss A, Hanusch C, Fasching PA, Blohmer JU, et al.: **A randomized, double-blind, phase III trial of neoadjuvant chemotherapy (NACT) with atezolizumab/placebo in patients with triple-negative breast cancer (TNBC) followed by adjuvant continuation of atezolizumab/placebo (Gepardouze).** *Oncology research and treatment* 2020, **43:**24‐25.

20. Euctr IT: **Randomized trial that compares, with the clinical practice monitoring, an adjuvant or neoadjuvant treatment with an anti PD L1 antibody in breast cancer patients with negative receptors.** [*https://trialsearchwhoint/Trial2aspx?TrialID=EUCTR2016-000189-45-IT*](https://trialsearchwhoint/Trial2aspx?TrialID=EUCTR2016-000189-45-IT) 2021.

21. Vidula N, Nanda R, Miller K, Emens L, Abramson V, Park B, Liu MC, Goga A, Rugo H: **Randomized phase II trial of pembrolizumab/carboplatin vs. carboplatin alone for breast cancer with chest wall recurrence: TBCRC044.** *Cancer research* 2022, **82**.

22. Garrido-Castro AC, Barry WT, Traina TA, Wesolowski R, Tung NM, Keenan T, Van Allen EM, Lin NU, Winer EP, Krop IE, et al.: **A randomized phase II trial of carboplatin with or without nivolumab in first- or secondline metastatic TNBC.** *Journal of clinical oncology* 2018, **36**.

23. Loibl S, Schneeweiss A, Burchardi N, Blohmer JU, Hanusch C, Costa SD, Huober J, Jackisch C, Von Minckwitz G, Kummel S, et al.: **A randomized phase II study to investigate the addition of PD-L1 antibody MEDI4673 (durvalumab) to a taxane-anthracycline containing chemotherapy in triple negative breast cancer (GeparNuevo).** *Annals of oncology* 2016, **27**.

24. McArthur HL, Comen EA, Solomon S, Rodine M, DiLauro Abaya C, Leal JHS, Patil S, Norton L: **A randomized phase II study of peri-operative ipilimumab, nivolumab and cryoablation versus standard peri-operative care in women with residual triple negative early stage/resectable breast cancer after standard-of-care neoadjuvant chemotherapy.** *Cancer research* 2019, **79**.

25. McArthur HL, Comen EA, Bryce Y, Solomon SB, Leal JHS, Rodine M, Abaya CD, Patil S, Page DB, Norton L: **A randomized phase II study of peri-operative ipilimumab, nivolumab and cryoablation versus standard care in women with residual, early stage/resectable, triple negative breast cancer after standard-of-care neoadjuvant chemotherapy.** *Annals of oncology* 2019, **30:**v97‐.

26. Vidula N, Goga A, Hwang J, Liu MC, Park BH, Nanda R, Pohlmann PR, Storniolo AM, Brufsky A, Abramson V, et al.: **A randomized phase II study of pembrolizumab in combination with carboplatin versus carboplatin alone in breast cancer patients with chest wall disease, with immunologic and genomic correlative studies.** *Cancer research* 2019, **79**.

27. Loibl S, Untch M, Burchardi N, Bodo Huober J, Blohmer JU, Grischke EM, Furlanetto J, Tesch H, Hanusch C, Rezai M, et al.: **Randomized phase II neoadjuvant study (GeparNuevo) to investigate the addition of durvalumab to a taxane-anthracycline containing chemotherapy in triple negative breast cancer (TNBC).** *Journal of clinical oncology* 2018, **36**.

28. Per: **A RANDOMIZED OPEN-LABEL PHASE III STUDY OF SINGLE AGENT PEMBROLIZUMAB VERSUS SINGLE AGENT CHEMOTHERAPY PER PHYSICIAN’S CHOICE FOR METASTATIC TRIPLE NEGATIVE BREAST CANCER (MTNBC) – (KEYNOTE-119).** [*https://trialsearchwhoint/Trial2aspx?TrialID=PER-066-15*](https://trialsearchwhoint/Trial2aspx?TrialID=PER-066-15) 2016.

29. Geyer CE, Loibl S, Rastogi P, Seiler S, Costantino JP, Nekljudova VN, Cortazar P, Lucas PC, Denkert C, Mamounas EP, et al.: **A randomized double-blind phase III clinical trial of neoadjuvant chemotherapy (NAC) with atezolizumab or placebo in patients (pts) with triple negative breast cancer (TNBC) followed by adjuvant atezolizumab or placebo: NSABP B-59/GBG 96-GeparDouze.** *Cancer research* 2019, **79**.

30. Loibl S, Untch M, Burchardi N, Huober J, Sinn BV, Blohmer JU, Grischke EM, Furlanetto J, Tesch H, Hanusch C, et al: **A randomised phase II study investigating durvalumab in addition to an anthracycline taxane-based neoadjuvant therapy in early triple-negative breast cancer: Clinical results and biomarker analysis of GeparNuevo study.** *Annals of Oncology* 2019, **30:**1279-1288.

31. Takano T, Cortes J, Cescon DW, Im SA, Yusof MM, Iwata H, Masuda N, Huang CS, Chung CF, Tsugawa K, et al.: **PS2-2 KEYNOTE-355 Asian subset: pembrolizumab + chemotherapy vs placebo + chemotherapy for triple-negative breast cancer.** *Annals of oncology* 2022, **33:**S465‐.

32. Turner N, Swift C, Jenkins B, Kilburn L, Coakley M, Beaney M, Fox L, Goddard K, Garcia-Murillas I, Hall P, et al.: **Primary results of the cTRAK TN trial: a clinical trial utilising ctDNA mutation tracking to detect minimal residual disease and trigger intervention in patients with moderate and high risk early stage triple negative breast cancer.** *Cancer research* 2022, **82**.

33. Sinn BV, Loibl S, Karn T, Untch M, Kunze CA, Weber KE, Treue D, Wagner K, Hanusch CA, Klauschen F, et al.: **Pre-therapeutic PD-L1 expression and dynamics of Ki-67 and gene expression during neoadjuvant immune-checkpoint blockade and chemotherapy to predict response within the GeparNuevo trial.** *Cancer research* 2019, **79**.

34. Emens L, Adams S, Loi S, Schmid P, Schneeweiss A, Rugo H, Chui S, Winer E: **A phase Ill randomized trial of atezolizumab in combination with nab-paclitaxel as first line therapy for patients with metastatic triple-negative breast cancer (mTNBC).** *Cancer Research* 2016, **76**.

35. Euctr ES: **A Phase III, Randomized, Double-blind Study to Evaluate Chemotherapy plus Pembrolizumab vs Chemotherapy plus Placebo as Neoadjuvant Therapy and Pembrolizumab vs Placebo as Adjuvant Therapy for Triple Negative Breast Cancer (TNBC).** [*https://trialsearchwhoint/Trial2aspx?TrialID=EUCTR2016-004740-11-ES*](https://trialsearchwhoint/Trial2aspx?TrialID=EUCTR2016-004740-11-ES) 2017.

36. Per: **A PHASE III, MULTICENTER, RANDOMIZED, OPEN-LABEL STUDY COMPARING ATEZOLIZUMAB (ANTI-PD-L1 ANTIBODY) IN COMBINATION WITH ADJUVANT ANTHRACYCLINE/TAXANE BASED CHEMOTHERAPY VERSUS CHEMOTHERAPY ALONE IN PATIENTS WITH OPERABLE TRIPLE NEGATIVE BREAST CANCER.** [*https://trialsearchwhoint/Trial2aspx?TrialID=PER-055-18*](https://trialsearchwhoint/Trial2aspx?TrialID=PER-055-18) 2019.

37. Per: **A PHASE III, DOUBLE-BLIND, PLACEBO-CONTROLLED, RANDOMIZED STUDY OF IPATASERTIB IN COMBINATION WITH ATEZOLIZUMAB AND PACLITAXEL AS A TREATMENT FOR PATIENTS WITH LOCALLY ADVANCED UNRESECTABLE OR METASTATIC TRIPLE-NEGATIVE BREAST CANCER.** [*https://trialsearchwhoint/Trial2aspx?TrialID=PER-018-19*](https://trialsearchwhoint/Trial2aspx?TrialID=PER-018-19) 2019.

38. Euctr HU: **A Phase III Study to Evaluate Chemotherapy With or Without Pembrolizumab as First Line Treatment for Triple Negative Breast Cancer.** [*https://trialsearchwhoint/Trial2aspx?TrialID=EUCTR2016-001432-35-HU*](https://trialsearchwhoint/Trial2aspx?TrialID=EUCTR2016-001432-35-HU) 2016.

39. Conte PF, Dieci MV, Bisagni G, De Laurentiis M, Tondini CA, Schmid P, De Salvo GL, Moratello G, Guarneri V: **Phase III randomized study of adjuvant treatment with the ANTIPD-L1 antibody avelumab for high-risk triple negative breast cancer patients: the A-BRAVE trial.** *Journal of clinical oncology* 2020, **38**.

40. Yusof MM, Cescon DW, Rugo HS, Im SA, Gallardo C, Lipatov O, Barrios CH, Holgado E, Iwata H, Masuda N, et al: **Phase III KEYNOTE-355 study of pembrolizumab (pembro) vs placebo (pbo) plus chemotherapy (chemo) for previously untreated locally recurrent inoperable or metastatic triple-negative breast cancer (TNBC): Results for patients (Pts) enrolled in Asia.** *Annals of Oncology* 2020, **31:**S1257-S1257.

41. Lander EM, Lehmann BD, Shah PD, Dees EC, Ballinger TJ, Pohlmann PR, Santa-Maria CA, Shyr Y, Mayer IA, Park BH, et al.: **A phase II trial of atezolizumab (anti-PD-L1) with carboplatin in patients with metastatic triplenegative breast cancer (mTNBC).** *Journal of clinical oncology* 2020, **38**.

42. Euctr NL: **Phase II study with nivolumab (anti-PD1) in patients with triple negative breast cancer after induction treatment.** [*https://trialsearchwhoint/Trial2aspx?TrialID=EUCTR2015-001969-49-NL*](https://trialsearchwhoint/Trial2aspx?TrialID=EUCTR2015-001969-49-NL) 2015.

43. Shah AN, Flaum L, Helenowski I, Santa-Maria CA, Jain S, Rademaker A, Nelson V, Tsarwhas D, Cristofanilli M, Gradishar W: **Phase II study of pembrolizumab and capecitabine for triple negative and hormone receptor-positive, HER2-negative endocrine-refractory metastatic breast cancer.** *J Immunother Cancer* 2020, **8**.

44. Obeid E, Miller KD, Sparano JA, Blackwell K, Goldstein LJ: **A Phase II randomized trial of pembrolizumab with carboplatin and gemcitabine for treatment of patients with metastatic triple-negative breast cancer (mTNBC).** *Cancer research* 2017, **77**.

45. Brufsky A, Kim SB, Zvirbule Z, Dirix LY, Eniu AE, Carabantes F, Izarzugaza Y, Mebis J, Sohn J, Wongchenko M, et al.: **Phase II COLET study: atezolizumab (A) + cobimetinib (C) + paclitaxel (P)/nab-paclitaxel (nP) as first-line (1L) treatment (tx) for patients (pts) with locally advanced or metastatic triple-negative breast cancer (mTNBC).** *Journal of clinical oncology* 2019, **37**.

46. Yuan Y, Frankel P, Synold T, Lee P, Yost S, Martinez N, Tang A, Mendez B, Schmolze D, Apple S, et al: **A phase II clinical trial of the combination of pembrolizumab and selective androgen receptor modulator GTx-024 in patients with advanced androgen receptor positive triple negative breast cancer.** *Cancer Research* 2018, **78**.

47. Yuan Y, Lee JS, Yost SE, Frankel PH, Ruel C, Egelston CA, Guo W, Gillece JD, Folkerts M, Reining L, et al: **A Phase II Clinical Trial of Pembrolizumab and Enobosarm in Patients with Androgen Receptor-Positive Metastatic Triple-Negative Breast Cancer.** *Oncologist* 2021, **26:**99-e217.

48. Devaux A, Canon JL, Duhoux FP, Delree P, Galant C, Coulie PG, Bar I, Constant M, Haussy S, Bricard O, et al.: **A phase Ib/II study of durvalumab combined with dose-dense EC in neoadjuvant setting for patients with locally advanced luminal B HER2(-) or triple negative breast cancers (B-IMMUNE).** *Annals of oncology* 2018, **29:**viii89‐.

49. Sun K, Xu Y, Zhang L, Niravath P, Darcourt J, Patel T, Teh BS, Farach AM, Guerrero C, Mathur S, et al: **A Phase 2 Trial of Enhancing Immune Checkpoint Blockade by Stereotactic Radiation and In Situ Virus Gene Therapy in Metastatic Triple-Negative Breast Cancer.** *Clinical cancer research : an official journal of the American Association for Cancer Research* 2022, **28:**4392-4401.

50. Nct: **Pembrolizumab, IRX-2, and Chemotherapy in Triple Negative Breast Cancer.** [*https://clinicaltrialsgov/show/NCT04373031*](https://clinicaltrialsgov/show/NCT04373031) 2020.

51. Kummel S, Cortes J, Dent R, Pusztai L, McArthur H, Bergh J, Denkert C, Park YH, Hui R, Harbeck N, et al.: **Pembrolizumab vs placebo + chemotherapy as neoadjuvant treatment, followed by pembrolizumab vs placebo as adjuvant treatment for early triple-negative breast cancer (TNBC): phase 3 KEYNOTE-522 study.** *European journal of surgical oncology* 2022, **48:**e30‐e31.

52. Winer E, Lipatov O, Im SA, Goncalves A, Lee KS, Schmid P, Testa L, Witzel I, Ohtani S, Turner N, et al.: **Pembrolizumab versus chemotherapyfor previously treated metastatic triple-negative breastcancer (KEYNOTE-119): efficacy in patients with lung orliver metastases.** *Cancer research* 2021, **81**.

53. Isaac D: **Pembrolizumab Plus Neoadjuvant Chemotherapy Improves Pathologic Complete Response Rates in Triple-Negative Breast Cancer.** *Journal of Clinical Outcomes Management* 2020, **27:**53-55.

54. Cortes J, Rugo HS, Cescon DW, Im SA, Yusof MM, Gallardo C, Lipatov O, Barrios CH, Perez-Garcia J, Iwata H, et al: **Pembrolizumab plus Chemotherapy in Advanced Triple-Negative Breast Cancer.** *New England Journal of Medicine* 2022, **387:**217-226.

55. Schmid P, Salgado R, Park YH, Muñoz-Couselo E, Kim SB, Sohn J, Im SA, Foukakis T, Kuemmel S, Dent R, et al: **Pembrolizumab plus chemotherapy as neoadjuvant treatment of high-risk, early-stage triple-negative breast cancer: results from the phase 1b open-label, multicohort KEYNOTE-173 study.** *Annals of Oncology* 2020, **31:**569-581.

56. Nct: **Pembrolizumab in Treating Patients With Triple-Negative Breast Cancer.** [*https://clinicaltrialsgov/show/NCT02954874*](https://clinicaltrialsgov/show/NCT02954874) 2016.

57. Sidaway P: **Pembrolizumab improves EFS in TNBC.** *Nature Reviews Clinical Oncology* 2022, **19:**220-220.

58. Rauch GM, Beatriz AE, Candelaria RP, Elshafeey N, Abdelhafez AH, Musall BC, Sun J, Boge M, Mohamed RMM, Son JB, et al.: **Patient-reported outcomes from thePhase III IMpassion031 trial of neoadjuvant atezolizumab+ chemotherapy in early triple-negative breast cancer.** *Cancer research* 2021, **81**.

59. Ramalingam S, Arora S, Whipple Neibauer M, Zhou J, Hazard S, Frenkl T, Stojadinovic A, Peters S: **P83.02 Niraparib + Pembrolizumab (Pembro) Versus Placebo + Pembro 1L Maintenance Therapy in Advanced NSCLC: ZEAL-1L Phase III Study.** *Journal of thoracic oncology* 2021, **16:**S653‐S654.

60. Geyer CE, Loibl S, Rastogi P, Seiler S, Costantino JP, Nekljudova V, Cortazar P, Lucas PC, Denkert C, Mamounas EP, et al.: **NSABP B-59/GBG 96-GeparDouze: a randomized double-blind phase III clinical trial of neoadjuvant chemotherapy (NAC) with atezolizumab or placebo in patients (pts) with triple-negative breast cancer (TNBC) followed by adjuvant atezolizumab or placebo.** *Journal of clinical oncology* 2019, **37**.

61. Geyer CE, Loibl S, Rastogi P, Seiler S, Costantino JP, Vijayvergia N, Cortazar P, Lucas PC, Denkert C, Mamounas EP, et al.: **NSABP B-59/GBG 96-GeparDouze: a randomized double-blind phase III clinical trial of neoadjuvant chemotherapy (NAC) with atezolizumab or placebo in Patients (pts) with triple negative breast cancer (TNBC) followed by adjuvant atezolizumab or placebo.** *Journal of clinical oncology* 2018, **36**.

62. Khoury K, Isaacs C, Gatti-Mays ME, Donahue RN, Schlom J, Wang H, Gallagher C, Graham D, Warren R, Dilawari A, et al.: **Nivolumab or capecitabine or combination therapy as adjuvant therapy for triple negative breast cancer (TNBC) with residual disease following neoadjuvant chemotherapy: the OXEL study.** *Cancer research* 2019, **79**.

63. Nct: **Nivolumab After Induction Treatment in Triple-negative Breast Cancer (TNBC) Patients.** [*https://clinicaltrialsgov/show/NCT02499367*](https://clinicaltrialsgov/show/NCT02499367) 2015.

64. Sidaway P: **Neoadjuvant therapy improves pCR rate.** *Nature Reviews Clinical Oncology* 2020, **17:**718-718.

65. **Neoadjuvant Pembrolizumab Takes on TNBC.** *Cancer discovery* 2019, **9:**OF4.

66. Ray PS, Ray T, Hussa R: **LBA2 Assessment of Ki67 and FOXC1-based response predictor tracking proliferation and plasticity as a complementary diagnostic for neoadjuvant olaparib+paclitaxel+durvalumab in primary triple-negative breast cancer: retrospective analysis of the I-SPY2 trial.** *Annals of oncology* 2022, **33:**S123‐.

67. Schmid P, Baccan C, Guo Z, Tryfonidis K, Rugo HS: **KEYNOTE-B49: a phase 3, randomized, double-blind, placebo-controlled study of pembrolizumab plus chemotherapy in patients with HR+/HER2-locally recurrent inoperable or metastatic breast cancer.** *Cancer research* 2022, **82**.

68. Schmid P, Cortes J, Dent R, Pusztai L, McArthur HL, Kuemmel S, Bergh J, Denkert C, Park YH, Hui R, et al.: **KEYNOTE-522: phase III study of pembrolizumab (pembro) 1 chemotherapy (chemo) vs placebo (pbo) 1 chemo as neoadjuvant treatment, followed by pembro vs pbo as adjuvant treatment for early triple-negative breast cancer (TNBC).** *Annals of oncology* 2019, **30:**v853‐v854.

69. Schmid P, Cortes Castan J, Bergh J, Pusztai L, Denkert C, Verma S, McArthur HL, Zhao J, Aktan G, Dang T, et al.: **KEYNOTE-522: phase III study of pembrolizumab (pembro) 1 chemotherapy (chemo) vs placebo 1 chemo as neoadjuvant followed by pembro vs placebo as adjuvant therapy for triple-negative breast cancer (TNBC).** *Annals of oncology* 2017, **28:**v72‐.

70. McArthur H, Cortes J, Dent R, Pusztai L, Kummel S, Bergh J, Denkert C, Park YH, Hui R, Harbeck N, et al.: **KEYNOTE-522: neoadjuvant Pembrolizumab + Chemotherapy vs Placebo + Chemotherapy Followed by Adjuvant Pembrolizumab vs Placebo for Early-stage Triple Negative Breast Cancer.** *Annals of surgical oncology* 2022, **29:**S338‐.

71. Schmid P: **KEYNOTE-522 study of pembro + chemo vs. placebo + chemo as neoadjuvant treatment followed by pembro vs. placebo as adjuvant treatment for early TNBC: pathologic complete response in key subgroups.** *Annals of surgical oncology* 2020, **27:**S129‐.

72. Cortes Castan J, Guo Z, Karantza V, Aktan G: **KEYNOTE-355: randomized, double-blind, phase III study of pembrolizumab (pembro) + chemotherapy (chemo) vs placebo (pbo) + chemo for previously untreated, locally recurrent, inoperable or metastatic triple-negative breast cancer (mTNBC).** *Annals of oncology* 2017, **28:**x25‐.

73. Takano T, Cortes J, Cescon DW, Im SA, Yusof MM, Iwata H, Masuda N, Huang CS, Chung CF, Tsugawa K, et al: **KEYNOTE-355 Asian subset: Pembrolizumab plus chemotherapy vs placebo plus chemotherapy for triple-negative breast cancer.** *Annals of Oncology* 2022, **33:**S465-S465.

74. Cortes J, Lipatov O, Im SA, Goncalves A, Lee KS, Schmid P, Tamura K, Testa L, Witzel I, Ohtani S, et al.: **KEYNOTE-119: phase III study of pembrolizumab (pembro) versus single-agent chemotherapy (chemo) for metastatic triple negative breast cancer (mTNBC).** *Annals of oncology* 2019, **30:**v859‐v860.

75. Winer EP, Dang T, Karantza V, Su SC: **KEYNOTE-119: a randomized phase III study of single-agent pembrolizumab (MK-3475) vs single-agent chemotherapy per physician's choice for metastatic triple-negative breast cancer (mTNBC).** *Journal of clinical oncology* 2016, **34**.

76. Dent R, Andre F, Goncalves A, Kummel S, Martin M, Schmid P, Schuetz F, Swain SM, Easton V, Pollex E, et al.: **IMpassion132: a double-blind randomized phase 3 trial evaluating chemotherapy (CT) ± atezolizumab (atezo) for early progressing locally advanced/metastatic triple-negative breast cancer (mTNBC).** *Journal of clinical oncology* 2018, **36**.

77. Cortés J, André F, Gonçalves A, Kümmel S, Martín M, Schmid P, Schuetz F, Swain SM, Easton V, Pollex E, et al: **IMpassion132 Phase III trial: Atezolizumab and chemotherapy in early relapsing metastatic triple-negative breast cancer.** *Future Oncology* 2019, **15:**1951-1961.

78. Miles D, Andre F, Gligorov J, Verma S, Xu B, Cameron D, Barrios CH, Schneeweiss A, Easton V, Ghazi Y, et al.: **IMpassion131: a phase III study comparing 1L atezolizumab with paclitaxel vs placebo with paclitaxel in treatment-naive patients with inoperable locally advanced or metastatic triple negative breast cancer (TNBC).** *Cancer research* 2018, **78**.

79. Schmid P, Adams S, Rugo HS, Schneeweiss A, Barrios CH, Iwata H, Dieras V, Henschel V, Molinero L, Chui SY, et al.: **IMpassion130: updated overall survival (OS) from a global, randomized, double-blind, placebo-controlled, Phase III study of atezolizumab (atezo) + nabpaclitaxel (nP) in previously untreated locally advanced or metastatic triple-negative breast cancer (mTNBC).** *Journal of clinical oncology* 2019, **37**.

80. Schmid P, Adams S, Rugo HS, Schneeweiss A, Barrios CH, Iwata H, Dieras V, Hegg R, Im SA, Wright GS, et al.: **IMpassion130: results from a global, randomised, double-blind, phase III study of atezolizumab (atezo) + nab-paclitaxel (nab-P) vs placebo + nab-P in treatment-naive, locally advanced or metastatic triple-negative breast cancer (mTNBC).** *Annals of oncology : official journal of the european society for medical oncology* 2018, **29:**viii707‐viii708.

81. Von Moos R, Emens LA, Loi S, Rugo HS, Schneeweiss A, Dieras V, Iwata H, Barrios CH, Nechaeva M, Molinero L, et al.: **IMpassion130: efficacy in immune biomarker subgroups of atezolizumab + nab-paclitaxel in patients with triple-negative BC.** *Swiss medical weekly* 2019, **149:**13S‐14S.

82. Emens LA, Adams S, Loi S, Schneeweiss A, Rugo HS, Winer EP, Barrios CH, Dieras V, De La Haba-Rodriguez J, Gianni L, et al.: **IMpassion130: a Phase III randomized trial of atezolizumab with nab-paclitaxel for first-line treatment of patients with metastatic triple-negative breast cancer (mTNBC).** *Journal of clinical oncology* 2016, **34**.

83. Mittendorf E, Barrios CH, Harbeck N, Miles D, Saji S, Zhang H, Duc AN, Rafii S, Lai C: **IMpassion031: a phase III study comparing neoadjuvant atezolizumab vs placebo in combination with nab-paclitaxel-based chemotherapy in early triple-negative breast cancer (TNBC).** *Cancer research* 2018, **78**.

84. Schmid P, Haiderali A, Mejia J, Guo Z, Zhou X, Martin-Nguyen A, Cortes J, Winer E: **Impact of pembrolizumab versus chemotherapy on health-related quality of life in patients with metastatic triple negative breast cancer.** *Annals of oncology* 2020, **31:**S65‐S66.

85. Loibl S, Jackisch C, Rastogi P, Seiler S, Lucas PC, Denkert C, Costantino J, Nekljudova V, Wolmark N, Geyer C: **GeparDouze/NSABP B-59: a randomized double-blind phase III clinical trial of neoadjuvant chemotherapy with atezolizumab or placebo in patients with triple negative breast cancer (TNBC) followed by adjuvant atezolizumab or placebo.** *Annals of oncology* 2019, **30:**iii38‐.

86. Emens LA, Adams S, Barrios CH, Diéras V, Iwata H, Loi S, Rugo HS, Schneeweiss A, Winer EP, Patel S, et al: **First-line atezolizumab plus nab-paclitaxel for unresectable, locally advanced, or metastatic triple-negative breast cancer: IMpassion130 final overall survival analysis.** *Annals of Oncology* 2021, **32:**983-993.

87. Cortes J, Cescon DW, Rugo HS, Nowecki Z, Im SA, Yusof MM, Gallardo C, Lipatov O, Barrios CH, Perez-Garcia J, et al.: **Final results of KEYNOTE-355: randomized, double-blind, phase 3 study of pembrolizumab + chemotherapy vs placebo + chemotherapy for previously untreated locally recurrent inoperable or metastatic triple-negative breast cancer.** *Cancer research* 2022, **82**.

88. Shah M, Osgood CL, Amatya AK, Fiero MH, Pierce WF, Nair A, Herz J, Robertson KJ, Mixter BD, Tang S, et al.: **FDA Approval Summary: pembrolizumab for Neoadjuvant and Adjuvant Treatment of Patients with High-Risk Early-Stage Triple Negative Breast Cancer.** *Clinical cancer research* 2022.

89. Savas P, Loi S: **Expanding the Role for Immunotherapy in Triple-Negative Breast Cancer.** *Cancer Cell* 2020, **37:**623-624.

90. Schmid P, Cortes J, Dent R, Pusztai L, McArthur H, Kümmel S, Bergh J, Denkert C, Park YH, Hui R, et al: **Event-free Survival with Pembrolizumab in Early Triple-Negative Breast Cancer.** *New England Journal of Medicine* 2022, **386:**556-567.

91. Pusztai L, Han HS, Yau C, Wolf D, Wallace AM, Shatsky R, Helsten T, Boughey JC, Haddad T, Stringer-Reasor E, et al.: **Evaluation of durvalumab in combination with olaparib and paclitaxel in high-risk HER2 negativestage II/III breast cancer: results from the I-SPY 2 TRIAL.** *Cancer research* 2020, **80**.

92. **Early Pembrolizumab Ups TNBC Responses.** *Cancer discovery* 2019, **9:**1638.

93. Loibl S, Schneeweiss A, Bodo Huober J, Braun M, Rey J, Blohmer JU, Furlanetto J, Zahm DM, Hanusch C, Thomalla J, et al.: **Durvalumab improves long-term outcome in TNBC: results from the phase II randomized GeparNUEVO study investigating neodjuvant durvalumab in addition to an anthracycline/taxane based neoadjuvant chemotherapy in early triple-negative breast cancer (TNBC).** *Journal of clinical oncology* 2021, **39**.

94. Dalenc F, Garberis I, Filleron T, Lusque A, Bachelot T, Arnedos M, Campone M, Sablin MP, Bonnefoi H, Jimenez M, et al.: **Durvalumab compared to maintenance chemotherapy in patients with metastatic breast cancer: results from phase II randomized trial SAFIR02-IMMUNO.** *Cancer research* 2020, **80**.

95. Sammons S, Tan TJY, Traina TA, Kim SB, Im YH, Bachelder C, Marcom PK, Dent RA: **Dora: a randomized phase II multicenter maintenance study of olaparib alone or olaparib in combination with durvalumab in platinum responsive advanced triple-negative breast cancer (aTNBC).** *Journal of clinical oncology* 2019, **37**.

96. Euctr DE: **Comparison of neoadjuvant chemotherapy with PDL1-inhibition (Atezolizumab) and Atezolizumab two-week window to chemotherapy with PDL1-inhibition (Atezolizumab) and identifying biomarkers predicting (early) response to or resistance against Atezolizumab (alone and with CTX) allowing patients stratification in future clinical trials.** [*https://trialsearchwhoint/Trial2aspx?TrialID=EUCTR2020-001651-40-DE*](https://trialsearchwhoint/Trial2aspx?TrialID=EUCTR2020-001651-40-DE) 2020.

97. Miles D, Kim SB, McNally V, Simmons B, Wongchenko M, Xu N, Brufsky A: **COLET: a multistage, phase 2 study evaluating the safety and efficacy of a doublet regimen of cobimetinib (C) in combination with paclitaxel (P) or triplet regimens of C in combination with atezolizumab (atezo) plus either P or nabpaclitaxel (nab-P) in metastatic triple-negative breast cancer (TNBC).** *Cancer research* 2017, **77**.

98. Nct: **Clinical Trial of Neoadjuvant Chemotherapy With Atezolizumab or Placebo in Patients With Triple-Negative Breast Cancer Followed After Surgery by Atezolizumab or Placebo.** [*https://clinicaltrialsgov/show/NCT03281954*](https://clinicaltrialsgov/show/NCT03281954) 2017.

99. Nct: **Carboplatin With or Without Atezolizumab in Treating Patients With Stage IV Triple Negative Breast Cancer.** [*https://clinicaltrialsgov/show/NCT03206203*](https://clinicaltrialsgov/show/NCT03206203) 2017.

100. Nct: **Avelumab With Binimetinib, Utomilumab, or Anti-OX40 Antibody PF-04518600 in Treating Triple Negative Breast Cancer.** [*https://clinicaltrialsgov/show/NCT03971409*](https://clinicaltrialsgov/show/NCT03971409) 2019.

101. Salgado AC, Garcia JMP, Castedo SPC, Cortes MG, Murillo SM, Lopez-Barajas IB, Blanch S, Plaza IC, Fernandez ND, Marme F, et al: **ATRACTIB: A phase II trial of first-line (1L) atezolizumab (A) in combination with paclitaxel (P) and bevacizumab (B) in metastatic triple-negative breast cancer (mTNBC).** *Annals of Oncology* 2022, **33:**S221-S221.

102. Schmid P, Rugo HS, Adams S, Schneeweiss A, Barrios CH, Iwata H, Diéras V, Henschel V, Molinero L, Chui SY, et al: **Atezolizumab plus nab-paclitaxel as first-line treatment for unresectable, locally advanced or metastatic triple-negative breast cancer (IMpassion130): updated efficacy results from a randomised, double-blind, placebo-controlled, phase 3 trial.** *The Lancet Oncology* 2020, **21:**44-59.

103. Schmid P, Adams S, Rugo HS, Schneeweiss A, Barrios CH, Iwata H, Diéras V, Hegg R, Im SA, Shaw Wright G, et al: **Atezolizumab and Nab-Paclitaxel in Advanced Triple-Negative Breast Cancer.** *N Engl J Med* 2018, **379:**2108-2121.

104. Winer EP, Lipatov O, Im SA, Goncalves A, Munoz-Couselo E, Seok Lee K, Schmid P, Testa L, Witzel I, Ohtani S, et al.: **Association of tumor mutational burden (TMB) and clinical outcomes with pembrolizumab (pembro) versus chemotherapy (chemo) in patients with metastatic triple-negative breast cancer (mTNBC) from KEYNOTE-119.** *Journal of clinical oncology* 2020, **38**.

105. Kyte JA, Røssevold A, Falk RS, Naume B: **ALICE: A randomized placebo-controlled phase II study evaluating atezolizumab combined with immunogenic chemotherapy in patients with metastatic triple-negative breast cancer.** *Journal of Translational Medicine* 2020, **18**.

106. Ignatiadis M, McArthur HL, Bailey A, Martinez J, De Azambuja E, Metzger O, Lai C, Franzoi MA, Goulioti T, Daly F, et al.: **ALEXANDRA/IMpassion030: a phase III study of standard adjuvant chemotherapy with or without atezolizumab in early stage triple negative breast cancer.** *Annals of oncology* 2019, **30:**v97‐.

107. Rugo HS, Schmid P, Cescon DW, Nowecki Z, Im SA, Yusof MMD, Gallardo C, Lipatov O, Barrios CH, Perez-Garcia J, et al.: **Additional efficacy endpoints from thephase 3 KEYNOTE-355 study of pembrolizumab pluschemotherapy vs placebo plus chemotherapy as first-linetherapy for locally recurrent inoperable or metastatic triple-negative breast cancer.** *Cancer research* 2021, **81**.

108. Kok M, Horlings HM, Van De Vijver K, Wiersma T, Russell N, Voorwerk L, Sikorska K, Van Werkhoven E, Mandjes IA, Kemper I, et al.: **Adaptive phase II randomized non-comparative trial of nivolumab after induction treatment in triple negative breast cancer: tONIC-trial.** *Annals of oncology* 2017, **28:**v608‐.

109. Ramalingam SS, de Castro G, Garassino MCC, Mazieres J, Sanborn RE, Smit EFF, Spigel DR, Thomas M, Velcheti V, Zhi E, et al.: **1360TiP First-line (1L) maintenance therapy with niraparib (nira) + pembrolizumab (pembro) vs placebo + pembro in advanced/metastatic non-small cell lung cancer (NSCLC): phase III ZEAL-1L study.** *Annals of oncology* 2021, **32:**S1030‐S1032.

110. Iwata H, Emens L, Adams S, Barrios CH, Dieras V, Loi S, Rugo HS, Schneeweiss A, Winer EP, Patel S, et al.: **49MO IMpassion130: final OS analysis from the pivotal phase III study of atezolizumab + nab-paclitaxel vs placebo + nab-paclitaxel in previously untreated locally advanced or metastatic triple-negative breast cancer.** *Annals of oncology* 2020, **31:**S1261‐S1262.

111. Im SA, Cortes J, Lipatov O, Goncalves A, Lee KS, Schmid P, Tamura K, Testa L, Witzel I, Ohtani S, et al.: **44O Pembrolizumab (pembro) vs chemotherapy (chemo) for previously treated metastatic triple-negative breast cancer (mTNBC): KEYNOTE-119 Asia-Pacific subpopulation.** *Annals of oncology* 2020, **31:**S1258‐.

112. Yusof MM, Cescon DW, Rugo HS, Im SA, Gallardo C, Lipatov O, Barrios CH, Holgado E, Iwata H, Masuda N, et al.: **43O Phase III KEYNOTE-355 study of pembrolizumab (pembro) vs placebo (pbo) + chemotherapy (chemo) for previously untreated locally recurrent inoperable or metastatic triple-negative breast cancer (TNBC): results for patients (Pts) enrolled in Asia.** *Annals of oncology* 2020, **31:**S1257‐.

113. Saji S, Mittendorf E, Harbeck N, Zhang H, Barrios CH, Hegg R, Koehler A, Sohn J, Iwata H, Telli ML, et al.: **3MO IMpassion031: results from a phase III study of neoadjuvant (neoadj) atezolizumab + chemo in early triple-negative breast cancer (TNBC).** *Annals of oncology* 2020, **31:**S1243‐.

114. Dent R, Cortes J, Pusztai L, McArthur HL, Kuemmel S, Bergh J, Denkert C, Park YH, Hui R, Harbeck N, et al.: **1O KEYNOTE-522 Asian subgroup: phase III study of neoadjuvant pembrolizumab (pembro) vs placebo (pbo) + chemotherapy (chemo) followed by adjuvant pembro vs pbo for early triple-negative breast cancer (TNBC).** *Annals of oncology* 2020, **31:**S1241‐S1242.

115. Brufsky A, Kim SB, Zvirbule Ž, Eniu A, Mebis J, Sohn JH, Wongchenko M, Chohan S, Amin R, Yan Y, et al: **A phase II randomized trial of cobimetinib plus chemotherapy, with or without atezolizumab, as first-line treatment for patients with locally advanced or metastatic triple-negative breast cancer (COLET): primary analysis.** *Annals of Oncology* 2021, **32:**652-660.

116. Hurvitz SA, Boni V, Comen E, Im SA, Jung KH, Kim SB, Lee KS, Loi S, Rugo HS, Sonnenblick A, et al.: **Phase Ib/II open-label, randomized trial of atezolizumab (atezo) with ipatasertib (ipat) and fulvestrant (fulv) vs control in MORPHEUS-HR+ breast cancer (M-HR+ BC) and atezo with ipat vs control in MORPHEUS triple negative breast cancer (M-TNBC).** *Cancer research* 2022, **82**.

117. Kok M, Voorwerk L, Horlings H, Sikorska K, Van der Vijver K, Slagter M, Warren S, Ong S, Wiersma T, Russell N, et al.: **Adaptive phase II randomized trial of nivolumab after induction treatment in triple negative breast cancer (TONIC trial): final response data stage I and first translational data.** *Journal of clinical oncology* 2018, **36**.

118. Yam C, Mittendorf EA, Sun R, Huo L, Damodaran S, Rauch GM, Candelaria RP, Adrada BE, Seth S, Symmans WF, et al.: **Neoadjuvant atezolizumab (atezo) and nab-paclitaxel (nab-p) in patients (pts) with triple-negative breast cancer (TNBC) with suboptimal clinical response to doxorubicin and cyclophosphamide (AC).** *Journal of clinical oncology* 2021, **39**.
